# Supplementary material for: HIV self-testing and partner notification strategies for key populations in low- to upper-middle-income countries: A mixed-methods systematic review
Source: PLoS One. 2025 Dec 29;20(12):e0338639. doi: 10.1371/journal.pone.0338639 (PMC12747344; doi:10.1371/journal.pone.0338639)
Supplement: S2 Table — (DOCX) [file pone.0338639.s002.docx]

| **S2 Table. Search strategy** | | |
| --- | --- | --- |
| **Databases** | **Search terms** | **# articles** |
| **Web Science** | All= ("HIV" OR "Human Immunodeficiency Virus") OR All= ("AIDS" OR "acquired immunodeficiency syndrome")  AND  All="Test*" OR All="Self-test*" OR All="Self-administered test*" OR All="Private test*" OR All="At-home test*” OR All= “Personal test*” OR All= “Autonomy test*” OR All= “Self-conducted test*”  AND  All=“Partner notification" OR All=“contact trac*" OR All="partner disclosure" OR All="Index test*" OR All="partner delivered" OR All="partner test*" OR All="Test* partner" OR All="Partner service" OR All=“partner referral” OR All=“partner elicitation” OR All=“sexual contacts” OR All=“tracing sexual contacts” OR All=“identification of contacts” OR All=“tracing exposed partners” | From 2016-2023.  561 articles |
| **Embase (Ovid)** | HIV.mp. or exp Human immunodeficiency virus or AIDS.mp. or exp acquired immune deficiency syndrome.  **AND**  exp rapid test/ or exp home diagnostic test/ or exp HIV test/ or exp self-administration test/ or exp prick test/ or exp screening test/ or exp infectious disease test kit/ or exp HIV rapid test/  **AND**  exp self-testing/ or "self-test*".mp. or "home-based test*".mp. or "self-initiated test*".mp. or "self-screen".mp. or "self-administer* near/5 test*".mp. or "private test*".mp. or "at-home test*".mp. or "personal test*".mp. or "autonomy test*".mp. or "self-conducted test*".mp.  **AND**  "Partner notification".mp. or exp contact examination/ or "contact trac*".mp or "partner disclosure".mp. or "index test*".mp. or "partner delivered".mp. or "partner test*".mp. or "test* partner".mp. or "partner service".mp. or "partner referral".mp. or "partner elicitation".mp. or "sexual contact*".mp. or "identification of contact*".mp. or "tracing exposed partner*".mp. | From 2016-2023.  1064 articles |
| **Global Health (Ovid)** | (("HIV" or "Human Immunodeficiency Virus") and ("AIDS" or "acquired immunodeficiency syndrome")  **AND**  ("Test*" or "Self-test*" or "Home-based test*" or “self-initiated test*” or “self-screen*” or “self-administered” or “private test*” or “at-home test*”, or “personal test*” or “autonomy test*” or “self-conducted test*”)  **AND**  ("Partner notification" or "contact trace*" or "partner disclosure" or "Index test*" or "partner delivered" or "partner test*" or "Test* partner" or “Partner service” or “partner referral” or “partner elicitation” or “sexual contacts” or “identification of contacts” or “tracing exposed partners”)).af. | From 2016-2023.  1530 articles |
| **Medline (Ovid)** | exp HIV/ or HIV.mp. or AIDS.mp. or exp acquired immunodeficiency syndrome/  **AND**  "Test*".mp. or exp Self-Testing/ or "Self-test*".mp. or "Home-based test*".mp. or "Self-initiated test*".mp. or "Self-screen test".mp. or "Self-administer*".mp. or "Private test*".mp. or "At-home test*".mp. or "Personal test*".mp. or "Autonomy test*".mp. or "self-conducted test*".mp.  **AND**  "Partner notification".mp. or exp Contact Tracing/ or "contact trac*".mp. or "Partner disclosure".mp. or "Index test*".mp.or "Partner delivered".mp. or "Partner test*".mp. or "Test* partner".mp. or "Partner service".mp. or "Partner referral".mp. or "Partner elicitation".mp. or "Sexual contact*".mp. | From 2016-2023.  621 articles |
| **Google Scholar** | HIV/AIDS AND Test AND “Partner Notification” | 300 |
