# Supplementary material for: HIV self-testing and partner notification strategies for key populations in low- to upper-middle-income countries: A mixed-methods systematic review
Source: PLoS One. 2025 Dec 29;20(12):e0338639. doi: 10.1371/journal.pone.0338639 (PMC12747344; doi:10.1371/journal.pone.0338639)
Supplement: S3 Table — (DOCX) [file pone.0338639.s003.docx]

| **Table S3. Qualitative meta-aggregation synthesized findings** | | | |
| --- | --- | --- | --- |
| **Synthesized findings** | **Category** | **Key findings** | **Illustrations** |
| **HIVST gains high acceptance and feasibility, primarily, and cost effectiveness, especially for partner notification and testing. Its success is supported by optimal timing, user competence, and targeted training for community health workers.** | 1.1. HIVST is highly accepted for its convenience, privacy, and cost-efficiency in testing its primary partners. | Index clients have demonstrated a high level of willingness to deliver HIV self-testing (HIVST) to their sexual partners, particularly among married and stable couples. (Unequivocal) | *“I have welcomed this tool [HIVST kit] with both hands. I have been trying to tell her [my wife] to get tested so that she should know her HIV status just like I have known mine.”* Male/52/Married, p6 (Offorjebe et al., 2020). |
|  |  | Female clients have shown an interest in a way to test their male partners who had previously refused facility-based testing due to lack of privacy and time constrain. (Unequivocal) | *“I like it [HIVST] because most of the time I am hesitant since men think differently from women. . . When women come here and test HIV positive, they explain to their husbands, but men are not open. I am very happy because maybe this is one way that my partner should know his HIV status since he has been refusing to come here [health facility] to get tested.”* Female/47/Married, p6 (Offorjebe et al., 2020). |
|  |  | Nearly all clients believed their partner would be happy to receive an HIVST kit, with the majority stating that bringing home a kit could strengthen their relationship. (Unequivocal) | *“Yes, it [the relationship] can even get stronger, and not get complicated. . . because I have been open and explained to him what to do so that his future will be somewhere.”* Female/32/Married, p7 (Offorjebe et al., 2020). |
|  |  | Home-based HIVST was favoured due to accessibility, convenience, cost-saving benefits, and privacy. (Unequivocal) | *“I have loved this kit because he [my partner] will self-test at home; he is too shy to come to the hospital, which is a public place.”* Female/32/Married, p8 (Offorjebe et al., 2020). |
|  |  | Most healthcare providers and policymakers found that home-based testing was acceptable owing to its accessibility and suits convenience. (Unequivocal) | *“Home visits are good, because when calling someone, if you give someone a letter or call him by phone, that they will come, they won’t come. But if you follow them, they will be forced to ‘just test me’ because you have followed him.”* Healthcare worker, Zambia, p60 (Hershow et al., 2019) |
|  |  | Female sex workers were to distribute HIVST kits to their primary partners due to feelings of intimacy, love, financial support, and commitment. (Unequivocal) | *“Because he is the person I love, and I trust him. No wonder I decided to start with him first. I wanted him to know his status. . .I wanted him to know.”* 22-year-old woman, p5 (Maman et al.,2017) |
|  | 1.2. Perceived benefits of HIVST include convenience, cost savings, ease of use, partner involvement, and the need for CHW training. | Most of them believed that HIVST would save time and cost of travelling to the hospital to receive testing (Unequivocal) | *“I welcome it [HIVST] and feel good because my partner wouldn’t travel the distance to the hospital for an HIV test, but he will just test himself [with HIVST].”* Female/32/Married, p7 (Offorjebe et al., 2020). |
|  |  | Both the male and female index clients said HIVST will be convenient to use and can overcome their challenges to undergoing testing from traditional facility-based testing centres. (Unequivocal) | *“I welcome it [HIVST] and feel good because my partner wouldn’t travel the distance to the hospital for an HIV test, but he will just test himself [with HIVST].”* Female/32/Married, p7 (Offorjebe et al., 2020).  *“It will be easy for one to test herself, unlike going to the hospital frequently. So, it is better to have HIVST which would make people realize their status in good time [to not delay testing*].” Male/61/Married, p7 (Offorjebe et al., 2020). |
|  |  | Women believed that they could demonstrate the HIVST kits to their partners, and their partners could use and interpret the HIVST kit. Very few index clients believed that additional counselling was needed. (Unequivocal) | Narrative evidence from the study report on p7 (Offorjebe et al., 2020). |
|  |  | The CHWs expressed the need for training to facilitate partner notification using HIVST among MSM. (Unequivocal) | Narrative evidence from the study report on p97 (Tih et al., 2019). |
| **HIVST facilitates strategic communication and planning between partner testing, enhancing health safety, mutual disclosure, and supportive discussions on sexual health risks and prevention.** | 2.1. Strategic communication and planning are key to successfully initiating HIVST among partners, with tactics ranging from prior discussion to insisting on testing before sex to ensure health safety. | FSW participants found to it easier convince their primary partner to undergo HIVST when initiating the process together or through discussion about its importance, sometimes even resorting to withholding sex. (Unequivocal) | *“At first, he told me that, I was, what, I was playing around with him at first. He asked me how I had such things [self-test kits], and how come that I was very eager to know his status. So, I told him this is me and this is you. . .. okay, this man, I gave him this kit because he is always loving, to that boy of mine, so this is the man that I trusted, and more so, I always meet with him, no wonder I gave him the kits. And I told him, I had some kits. The first question he asked was, where did I get the kit from? So, I explained. At first, he did not want me to test him with the kit, because he thought, I was only going to test him and not me. So, I told him that we were free. I used the kit, and we tested each other, and it turned negative.”* 26-year-old Women, p6 (Maman et al., 2017) |
|  |  | Conversely, with their commercial clients, FSWs employed a strategy of insisting on HIVST before unprotected sex, despite the client testing negative, demonstrating their deliberate avoidance of unprotected sex to safeguard their health. (Unequivocal) | Narrative evidence from the study report on p10 (Maman et al., 2017). |
|  |  | Women’s strategic approach to initiating HIVST discussions with their partners, emphasises careful planning and communication to avoid confrontation. (Unequivocal) | *“He asked me “Where did this come from.” I told him “When I took the baby to the clinic I was referred to some researchers. I was asked questions which I answered and accepted that I would enter (join) the study ...I was told how to enter, how I could test myself with them (self-test kits), how long to wait and see two lines on the kit, one line means there is no virus and if it has two lines there is HIV. Then he asked how that thing is used then I told him. He did that (tested) at night.”* Anyango, 28, p4 (Agot et al., 2020). |
|  |  | Stakeholders also stressed the importance of prior communication by women with their partners for home-based HIVST to prevent surprises. (Unequivocal) | Narrative evidence from the study report on p59 (Hershow et al., 2019). |
|  |  | Most of the male and female index clients suggested evening during bedtime as the suitable time to discuss HIVST with their partners. (Unequivocal) | *“In the evening is a good time when we are in bed and we are chatting, so I can be open to explaining to him about this way [HIVST].”* Female/32/Married, p7 (Offorjebe et al., 2020).  *“Evening is the best time . . . During the day there are many things to be preoccupied with. . . but during the evening, we are just the two of us and we are there to make decisions.”* Male/52/Married, p7 (Offorjebe et al., 2020). |
|  |  | Most of the women and partners thought that partner notification was acceptable, and they shared that sending an invitation letter or phone call by a healthcare provider was an effective strategy to overcome partner refusal. (Unequivocal) | *“There needs to be someone to shake them [men] up. ‘You need to know your status!’ So, the phone method is good, you send letters to them…they cannot refuse, they can accept.”* (Male partner of HIV‐negative woman, Malawi, p59 (Hershow et al., 2019). |
|  | 2.2. HIVST for partner testing will promote discussion about risk and facilitate mutual disclosure and support. | The positive experience reported by FSWs in distributing HIVST to their primary partners provided an opportunity to discuss sexual risk and health. (Unequivocal) | *"The feeling was good... I felt like he trusted me, and he was free with me. The results came out negative... I felt good when I was clean [HIV-free], and our baby was safe too. He felt happy, he was just smiling and then he said I told you to trust me, I was fine, why bring the kit around? Next, I told him... yes this is a new kit, you just have to believe in it, let us just go and test it in the hospital again... then he told me it is fine, I will just do it because I am now sure of myself so much, yes."* 20 years old, FSW, p6 (Maman et al., 2017) |
|  |  | Women emphasized the importance of support from their partners after testing to reduce fear of rejection or violence and to enhance compliance with safer sex practices and treatment adherence (Unequivocal) | Narrative evidence from the study report on p7 (Agot., 2020) |
| **Effective HIV index testing and partner notification are facilitated by streamlined policies and supportive environments but are often hindered by significant relational and societal barriers such as mistrust, stigma, gender norms, and safety concerns.** | 3.1. HIVST partner notification faces some relational and societal barriers including mistrust, stigma, gender norms, and safety concerns, complicating partner testing and effective implementation. | FSWs faced various challenges with HIVST partner testing, including sexual and verbal abuse, conflict in the relationship caused by suspicion of promiscuous behaviour, the client’s reluctance to test, and uncertainty about the client’s HIV status. (Unequivocal) | *"He took it negatively... he changed and told me to keep quiet, and he pushed me immediately on the bed... he has to sleep with me without a condom... I was just crying as I didn’t know his status... slept with me without a CD [meaning condom] ... if he has given me [infected me with HIV] then ‘whose visitor was I going to be’ [meaning who was going to welcome her]."* 22 years old FSW. (p8 (Maman et al., 2017) |
|  |  | Challenges in HIVST distribution when index clients didn't disclose their HIV status to partners, hindering couples counselling and secondary distribution implementation. health professionals' reluctance, PLHIV's hesitation, and limited disclosure support strategies. (Unequivocal) | *“…..Health professional: Did you share your status with him?..Woman: No. I am very afraid. Health professional: Okay. But are you going to tell him one day?.. Woman: No (while lowering her head)...Health professional: Why? And yet you’ve been followed here for 14 years...Woman: I’m very scared. I would like him to find out from me one day, but I am very afraid....”* p7 (Boye et al., 2021) |
|  |  | Most of the index clients who are currently distrusting their relationship shared that their partner may question relationship trust if they bring HIVST home to test their partners. (Unequivocal). | *“If she doesn’t love her life that means she won’t accept the kit, she will say, ‘You don’t trust me’."* Male/19/Non-Married Partner, p8 (Offorjebe et al., 2020). |
|  |  | Women perceived their partner’s reluctance to get tested as stemming from the belief that they might have already contracted HIV, or that they did not care if they were to get infected. (Unequivocal) | *“… [my husband] never got tested. He doesn’t want to. He knows about my status, but he said he doesn’t care if he gets it too*.” Bintang, 25, WLWH, p9 (Rahmalia et al., 2022). |
|  |  | Male and female index clients are not confident of administering HIVST and some pregnant women explained that secondary shared that the act may lead to relationship conflict. (Unequivocal) | *“...According to my understanding, I think it [secondary distribution of self-test kits] can bring disagreements because a man is a difficult person. You find that we test, and she finds that I’m not well. Instead of me accepting, you find that I’ll start accusing...Meaning that it will be difficult for peace to prevail in homes.”* Male partner of HIV-positive woman, Zambia, p61 (Hershow et al., 2019) |
|  |  | Women fear disclosing their own HIV status due to concerns about fear of rejection and conflicting desires for childbearing. (Unequivocal) | *“We got married four months ago … I first met him as a client [at a massage parlour]. I have stopped working since we got married and moved back to [her hometown] while my husband works in Jakarta … he doesn’t know my status and I don’t know how to tell him … I wish we could have children, but I would have to tell him about this disease … So I don’t know what to do … ”* Intan, 31, WLWH, p7 (Rahmalia et al., 2022) |
|  |  | Incomplete information about index partners and safety concerns such as verbal abuse during phone tracing, threats of physical harm, and suspicion of being sexual partners of the index partners. (Unequivocal) | Narrative evidence from the study report on p78 (Wamuti et al., 2023) |
|  |  | Stigma and fear prevent HIV testing; non-disclosure to partners stems from fears of discord and shame, leading to avoidance of nearby ART centres and resistance to testing until illness occurs. (Unequivocal) | *“If I tell my wife she will leave and go to her mother’s home then what will happen to my children; who will take care of the children?* Male ART Counsellor, p52 (Selvaraj et al., 2017) |
|  |  | MSM fear disclosing HIV status due to potential outing of sexual orientation; migrant spouses, especially females, lack awareness and decision-making power to access testing. (Unequivocal) | *“They will feel very afraid that their family members will come to know his behaviour of MSM, and his wife will leave him and go. They have one more distress of their MSM behaviour also will be disclosed and if they got the infection from their friend they also will be caught.”* Female ART counsellor, p52 (Selvaraj et al., 2017) |
|  |  | Most index clients believed that gender norms may not affect the distribution of HIVST for partner testing, but some believed that power imbalance within the gender could hinder women from delivering HIVST to their male partners. (Unequivocal) | *“Problems can be there. I have seen that sometimes the woman goes and gets tested alone, and she goes home and explains [to her husband] that she went to the hospital and got tested. If a woman is to bring back a self-test kit as well, I believe the woman can get 2 or 3 questions about why she took the kit. The man would ask, “What were you thinking by getting this thing!’ So, most of the time a woman can be scared to explain, so at the end, the kit will not be given.”* Female/32/Married, p9, (Offorjebe et al., 2020). |
|  |  | Some HIV-positive pregnant mentioned that distributing HIVST by health workers at home may bridge their HIV confidentiality, noting that neighbours might assume they are HIV-positive, leading to stigma and discrimination. (Unequivocal) | *“When you start following us home, you find that maybe a neighbour had been followed before and so will know there’s nothing else, they are here for HIV testing”* HIV-positive woman, Zambia, p60 (Hershow et al., 2019) |
|  |  | Health professionals' reluctance, PLHIV's hesitation, and limited disclosure support strategies. (Unequivocal) | Narrative evidence from the study report on p8 (Boye et al., 2021) |
|  | 3.2. Streamlined policies, supportive environments, and coordinated services enhance the effectiveness of HIV index and partner testing. | Only a few clients believed that HIV-positive women may be at risk of IPV upon delivering HIVST. (Unequivocal) | *“It is as though the wife has undermined you, but on the contrary, your wife wants your life to be well just like she is. But because of short-temperedness, that’s when one [man] can beat his wife.”* Male/52/Married, p8 (Offorjebe et al., 2020). |
|  |  | Decentralizing HIV services and engaging peer educators, travel reimbursement and promoting positive attitudes among service providers can help in tackling index testing. (Unequivocal) | Narrative evidence from the study report on p7 (Selvaraj et al., 2017) |
|  |  | Couple testing on the same day among pregnant women facilitated partner testing. Husbands who are HIV-negative were supportive of their wife’s diagnosis. (Unequivocal) | Narrative evidence from the study report on p9 (Rahmalia et al., 2022) |
|  |  | Health workers expressed the importance of a uniform policy of index testing and partner notification and legal power to execute the interventions effectively. (Equivocal) | *“… the labour did not progress, so they referred me to [a public hospital] … Then the doctor came and told me I was HIV-positive … He said I had to be referred to [a provincial referral hospital] for caesarean section and he asked who I wanted to disclose to … My aunt was there too, and I preferred to tell her than to tell my husband … However, when we got to the [referral] hospital, another doctor asked my husband to sign [the consent form] before the surgery and told him my status … ”* Mita, 24, WLWH who stayed with the same partner, p9 (Rahmalia et al., 2022). |
|  |  | Participants shared that disclosing their status to their primary partner is one of their goals after diagnosis. (Unequivocal) | *“…If I hide (my HIV status) …I will not have the freedom to use medicine in her presence…someone can even dare to go toilet to drink medicine (laughter).”* [FGD – both genders], p957 (Sanga et al., 2023) |
|  |  | Most of the index clients believed that distributing HIVST to partners would not increase IPV. (Unequivocal). | *“There can’t be violence because it will depend on how you explain the thing [HIVST] to him. If you explain everything well there can’t be any violence.”* Female/32/Married, p8 (Offorjebe et al., 2020) |
| **Partner notification strategies for index testing to partner notification in the HIVST program should provide various options. Provider/assisted referrals are preferred by index clients and other key populations for safety and effectiveness. However, health workers recommend choice-based and modified passive referrals to balance feasibility, safety, and cost-effectiveness.** | 4.1. Partner notification preferences vary, with a preference for assisted referrals due to safety and effectiveness, despite concerns with passive methods | HIV- positive women index clients mentioned that they prefer partner notification and HIVST kit distribution, while HIV-negative women preferred home-based HIVST for partner testing. The other strategy implemented, which was found to be effective was invitation letters or phone calls to overcome partner refusal. (Unequivocal) | *Table 3. Pregnant/postpartum women’s and male partners’ perception of the most preferred male partner HIV testing modality.* p62 (Hershow et al., 2019). |
|  |  | Some healthcare providers and high-risk groups like sex workers and fishermen believe that the anonymous nature of provider referrals could undermine trust due to perceived untruthfulness and encourage scepticism and rumours. (Unequivocal) | *“Supposing you come to notify me, I might think that you intend to start a relationship with my wife. If you start delivering the message to me that some of your sexual partners may have been exposed to HIV, I might think that you are trying to influence me to drop my sexual partners such that you start a relationship with them.”* Fisherman, fishing community, p8 (Quinn et al., 2018) |
|  |  | Assisted partner referral was the most preferred method for index testing to partner notification, followed by contact and then passive referral. (Unequivocal) | *“I would not like to wait for the time I negotiated with the health worker to elapse without having disclosed to my partner and have him go test for HIV. I have to work on that myself before the set period elapses. On the other hand, if the healthcare provider contacts him and advises him to pass by the clinic and test for HIV if he is a clever man, he can easily suspect what is going on and then come home and cause chaos, making the situation very bad before even getting to know his HIV status.”* Sex worker, fishing community, p6 (Quinn et al., 2019) |
|  |  | Most participants cited passive referral as a risky approach due to intimate partner violence, relationship separation, and self-harm post-disclosure without the healthcare provider’s mediation. (Unequivocal) | *“It is like the one who tested first is the one who brought the infection. Many women have come here complaining that, “All the time my husband points fingers at me that I infected him with HIV.”* Healthcare provider, fishing community, p5 (Quinn et al., 2019) |
|  |  | Most participants also felt that contact referral could be beneficial to those index clients needing more time to process their HIV-positive result, especially married couples notifying primary partners to reduce abandonment risk. (Unequivocal) | *“But there is one challenge you find with [contract referral], for instance, if I come to you [healthcare provider] and then we agree on a set period, I can decide to keep my HIV-positive status confidential without even asking my wife to go for HIV testing. I keep silent because I am aware that any time you contact my wife. Do you see how I use this chance for the set period to ensure that she also gets infected with HIV? She might have been HIV negative; unfortunately, do you know that she might get infected with HIV just within the set period?”* Fisherman, fishing community, p9 (Quinn et al., 2019) |
|  |  | Participants indicated that passive referral could strengthen a couple’s relationship, including allowing a couple to support each other with adherence to antiretroviral treatment. (Unequivocal) | *“The positive aspect of this program is that it helps…you in adherence. This is because when you are taking your treatment, you will not fear being seen by your partner, and you will fear nothing. Even going to the hospital to get medicine, you will not fear.”* Healthcare provider, fishing community, p5 (Quinn et al., 2018) |
|  | 4.2. Health workers recommend choice-based and modified passive referrals for partner notification, balancing feasibility with safety and cost concerns | A modified passive referral method was proposed, where index clients initiate couple testing before disclosing their status to partners at health centres. (Unequivocal) | Narrative evidence from the study report on p6 (Quinn et al., 2018) |
|  |  | Health workers preferred passive referral for ease and cost-effectiveness but recommended assisted or contact referral for safety reasons. (Unequivocal) | Narrative evidence from the study report on p6 (Quinn et al., 2018) |
|  |  | Most health workers shared that a choice-based approach for partner notifications was the most feasible method but the only concerns they shared were follow-up tracing effectiveness, time lost in partner elicitation tracing and the high cost associated with the intervention. (Unequivocal) | *“But in actual sense, it’s the woman who knows the husband better. So, we should give them the choices”,* Healthcare worker, Zambia, p61 (Hershow et al., 2019) |
