# Supplementary material for: HIV self-testing and partner notification strategies for key populations in low- to upper-middle-income countries: A mixed-methods systematic review
Source: PLoS One. 2025 Dec 29;20(12):e0338639. doi: 10.1371/journal.pone.0338639 (PMC12747344; doi:10.1371/journal.pone.0338639)
Supplement: S4 Table — (DOCX) [file pone.0338639.s004.docx]

**Table S4. Full-text studies with inclusion-exclusion reasons**

| **S/No** | **Author** | **Study Aim** | **Date of extraction** | **Included/Excluded** | **Inclusion/Exclusion Reason** |
| --- | --- | --- | --- | --- | --- |
| 1 | Myers RS et al., 2016, Mozambique | To assess the acceptability, effectiveness, and safety of APS in a large, urban clinic in Maputo. | 1/1/2024 | Included | APN among new HIV cases. |
| 2 | Pintye J et al., 2019, Kenya | The objective of this study was to assess the acceptability and feasibility of providing HIV self-tests by women to their male partners at maternal and child health (MCH) and family planning (FP) clinics in Nairobi, Kenya. | 1/1/2024 | Excluded | Study Conducted in 2014; pre-2016. |
| 3 | Offorjebe OA et al., 2020, Malawi | To assess the perceived feasibility and acceptability of index partner HIVST by HIV-positive clients versus partner referral slips (standard of care) among HIV-positive clients in Malawi. | 1/1/2024 | Included | Index partner HIV self-testing |
| 4 | Kitenge MK et al., 2022, South Africa | This study aimed to describe the acceptability of unsupervised peer distribution of HIV oral self-tests (HIVST) as a method to scale up HIV testing among hard-to-reach populations in rural KwaZulu Natal, South Africa. | 2/1/2024 | Excluded | Wrong population-targeted the general population |
| 5 | Madsen T et al., 2020, Guinea-Bissau | To investigate the acceptance, preferred choice of method, and outcome of Partner Notification (PN) among HIV-infected patients in Guinea-Bissau, West Africa. Additionally, the study aimed to identify challenges in the implementation of PN in this setting. | 2/1/2024 | Included | Partner Notification |
| 6 | Edosa M et al., 2022, Ethiopia | To assess the magnitude and factors associated with index case HIV testing (ICHT) among HIV patients attending ART in Nekemte town public health facilities. | 2/1/2024 | Included | Index Case HIV Testing |
| 7 | Wango GN et al., 2023, Kenya | We assessed whether providing oral fluid-based HIV self-tests to adolescent girls and young women (AGYW) in Kenya for secondary distribution to their male partners would increase male partner HIV testing and couples testing. | 3/1/2024 | Excluded | Wrong population-targeted HIV-negative adolescent girls |
| 8 | Ajayi AI et al., 2019, South Africa | This study assessed how HIV risk perceptions, knowledge of one’s partners status, and discussion of HIV/sexually transmitted infections (STIs) with one’s sexual partner influence uptake of HIV testing. | 3/1/2024 | Excluded | Wrong population-targeted HIV-negative young university students, |
| 9 | Remera E et al., 2022, Rwanda | To assess the relative effectiveness of voluntary assisted partner notification (VAPN) modalities in identifying undiagnosed HIV infections. | 3/1/2024 | Included | Partner notification |
| 10 | Wondimu T et al., 2022, Ethiopia | Implementing effective and efficient case finding strategies is crucial to increasing pediatric antiretroviral therapy coverage; in Ethiopia, universal HIV testing is conducted for children presenting at high-risk entry points but yields low positivity at many sites, so we reassessed current case finding strategies by developing and evaluating a risk screening tool for children presenting at high-risk entry points. | 4/1/2024 | Excluded | Wrong outcome-development and evaluation of a risk screening tool |
| 11 | Mutale W et al., 2021, Zambia | To investigate a combination approach, using evidence-based strategies, to increase HIV testing in male partners of HIV-positive and HIV-negative pregnant women. | 4/1/2024 | Included | HIV self-test kits to partner notification |
| 12 | Dziva Chikwari C et al., 2020, Zimbabwe | We investigated the challenges and relational aspects of index linked HIV testing for children and adolescents in Zimbabwe to inform strategies to improve uptake and linkage to care. | 4/1/2024 | Excluded | Wrong population-children and adolescents as index contacts, and their caregivers |
| 13 | Ewuoso C, 2020, South African | This study evaluates the conflict between patient confidentiality and partner notification in Sero discordant relationships and argues that, based on a theoretical formulation of Ubuntu, health providers are obliged to facilitate communal friendship by notifying partners of HIV seroconversion to ensure appropriate care. | 5/1/2024 | Excluded | Wrong outcome-ethical dilemmas (confidentiality vs. partner notification) |
| 14 | Kahabuka C et al., 2017, Tanzania | To describe and evaluate a partner notification model that aims to reach previously undiagnosed sexual partners of people living with HIV in Tanzania, and to assess its effectiveness in closing the first '90' target. | 5/1/2024 | Excluded | Double Duplicate |
| 15 | Wango GN et al., 2021, Kenya | To assess adolescent girls perceptions of their ability to safely distribute HIV self-test kits to their sexual partners in Siaya County, Kenya. | 5/1/2024 | Excluded | Wrong population targeted on HIV-negative adolescent girls |
| 16 | Qiao S et al., 2016, China | To examine interpersonal factors associated with HIV partner disclosure among HIV infected people in China. | 6/1/2024 | Excluded | Wrong outcome; disclosure focus, pre-2016. |
| 17 | La-cuo Z et al., 2020, China | To analyze differences in spousal notification patterns among HIV discordant couples in two key AIDS epidemic areas of Sichuan Province, China. | 6/1/2024 | Excluded | Wrong outcome; spousal notification, not APN & not HIVST. |
| 18 | Selvaraj K et al., 2017, India | To assess the status of partner testing and factors influencing it among PLHIV registered in selected ART centres in Gujarat, India, from 2011-2015. | 6/1/2024 | Included | Partner testing of diagnosed PLHIV, |
| 19 | Sircar NR et al., 2020, Kenya | To examine how Kenya policies and practices implement a human rights-based approach to HIV testing and partner notification, using qualitative methods. | 7/1/2024 | Excluded | Wrong outcome; policy focus, not HIVST/index testing. |
| 20 | Teka Haile U et al., 2023, Ethiopia | To assess the proportion of partner and family-based index case HIV testing and identify associated factors among adult clients on ART follow-up in Woliso Town, Oromia, Ethiopia. | 7/1/2024 | Excluded | No full-text article; Conference abstract only |
| 21 | Tih PM et al., 2019, Cameroon | To describe the large-scale implementation of assisted partner notification services (aPNS) and overall programmatic achievements in a resource-limited setting. | 7/1/2024 | Included | Assisted HIV partner notification |
| 22 | Han H et al., 2019, Sub-Saharan Africa | To explore facilitators and barriers to sustainable implementation of assisted HIV partner notification training among providers in three sub-Saharan African countries. | 8/1/2024 | Excluded | Wrong outcome; focused on aPN training, not HIVST or index testing. |
| 23 | Hu QH et al., 2021, China | To compare the effect of assisted vs. passive partner notification (PN) on uptake of HIV testing among sexual partners of newly HIV-diagnosed MSM. | 8/1/2024 | Included | RCT with MSM index clients; evaluates assisted PN with HIVST, |
| 24 | Sharma M et al., 2018, Kenya | The study aimed to evaluate the cost-effectiveness and health impact of implementing Assisted Partner Services (aPS) for HIV testing and linkage to care in western Kenya. | 8/1/2024 | Included | APN; cost effectiveness |
| 25 | Golden MR et al., 2023, Mozambique | To assess the safety and effectiveness of assisted partner notification services during scale-up in Mozambique. | 9/1/2024 | Included | PN among newly diagnosed HIV cases |
| 26 | Aliza MW et al., 2019, Kenya | To describe barriers encountered and potential opportunities to providing aPNS to established patients living with HIV. | 9/1/2024 | Included | APN among PLHIV receiving HIV care, |
| 27 | Namimbi F et al., 2020, Uganda | To evaluate the implementation of assisted partner notification services in Kampala, Uganda, including uptake, effectiveness, and linkage to care. | 9/1/2024 | Excluded | No full article; conference abstract only. |
| 28 | Manguro G et al., 2019, Kenya | To assess whether integrating assisted partner notification into a key population program improves HIV case identification and linkage to antiretroviral treatment among sexual contacts of male and female sex workers in a low-resource setting in Kenya. | 10/1/2024 | Excluded | No full article; conference abstract only. |
| 29 | Kinera I, 2023, Uganda | To determine the utilization of assisted partner notification and associated factors among sexually active HIV positive adolescents aged 15-19 years at Baylor Clinic, Mulago, Kampala. | 10/1/2024 | Excluded | Wrong Publication; thesis, not peer-reviewed. |
| 30 | Hogben M., 2017, Kenya | To evaluate the effectiveness of assisted partner services for HIV case-finding among newly diagnosed individuals in Kenya. | 10/1/2024 | Excluded | Wrong publication type; commentary, not original research. |
| 31 | Cherutich P et al., 2017, Kenya | To establish whether or not assisted partner services increase HIV testing, diagnoses, and linkage to care among sex partners of people with HIV infections in Kenya. | 11/1/2024 | Included | APN among newly/recently diagnosed HIV-positive adult |
| 32 | Marcus U et al., 2023, Multiple | To assess the association between internalised homonegativity and partner notification practices following a syphilis or gonorrhoea diagnosis among men who have sex with men in 49 countries across four continents. | 11/1/2024 | Excluded | Wrong population; STI context, not HIV-positive individuals. |
| 33 | Yee LM et al., 2020, US | To understand attitudes toward male partner HIV testing among low-income, minority pregnant women and their partners in an urban, high HIV prevalence setting. | 11/1/2024 | Excluded | Wrong population; HIV-negative women and partners, not PLHIV. |
| 34 | Adeniyi OV et al., 2021, South Africa | Our study fills this gap by using a follow-up survey of postpartum women with HIV to examine if disclosure prevalence has improved compared to the proportion recorded at childbirth. We further assessed the reasons for non-disclosure and correlates of serostatus disclosure to sexual partners. | 12/1/2024 | Excluded | Wrong outcome; focused on disclosure, not HIVST or index testing. |
| 35 | Chelogoi E et al., 2021, Kenya | To investigate factors that obstruct assisted partner notification services in this setting. | 12/1/2024 | Included | APN among HIV clients; |
| 36 | Katamba C, 2013, Zambia | The specific objectives are to understand the perceived facilitators and barriers to HIV partner testing from the perspective of the health-care provider; to propose interventions necessary for improved HIV case finding; and to reach high risk but hard to reach populations in HIV programs such as middle-aged men and adolescent girls and young women (AGYW). | 12/1/2024 | Excluded | Wrong outcome; research protocol with no empirical results. |
| 37 | Kalichman S et al., 2021, South Africa | The objective was to evaluate the effects of enhanced STI partner notification counselling and provider-assisted partner services on partner referral and the incidence of STI diagnosis in Cape Town, South Africa. | 13/1/2024 | Excluded | Wrong outcome; disclosure focus, not HIVST/index testing. |
| 38 | Goyette MS et al., 2018, Kenya | To investigate whether the history of intimate partner violence (IPV) modified APS effectiveness and risk of relationship dissolution. | 13/1/2024 | Included | PLHIV index cases; assesses aPN effectiveness and safety. |
| 39 | Agot K et al., 2018, Kenya | To assess the occurrence of intimate partner violence (IPV) among women who accept an intervention involving the receipt of multiple self-tests for distribution to their sexual partners. Further, the study aims to compare IPV levels following the intervention to baseline levels of IPV. | 13/1/2024 | Included | HIVST used; pilot study on secondary distribution |
| 40 | Wamuti BM, 2017, Cameroon | To assess the effectiveness of contact tracing and referral methods for HIV testing among sexual partners, spouses, and children of newly diagnosed HIV patients in Cameroon through the referral method. | 14/1/2024 | Excluded | Wrong Publication; thesis, not peer-reviewed. |
| 41 | Opeyemi A et al., 2021, Nigeria | To evaluate HIV partner notification services at the University of Abuja Teaching Hospital, comparing their effectiveness in identifying positive cases to other testing methods at the facility. | 14/1/2024 | Included | APN among HIV clients |
| 42 | Yeganeh N et al., 2019, Brazil | To explore challenges and motivators for male partner involvement in prenatal care for HIV testing in a tertiary care setting in Brazil. | 14/1/2024 | Excluded | Wrong population; HIV-negative partners, not PLHIV. |
| 43 | Thopola MK, 2020, South Africa | To identify challenges encountered by males regarding partner notification of sexually transmitted infections in rural clinics in Limpopo Province, South Africa. | 15/1/2024 | Excluded | Wrong outcome; STI focus, not HIVST/index testing. |
| 44 | Boye S et al., 2021, Mali | To improve our understanding of the practices, limitations and issues related to the distribution of HIV self-tests to PLHIV so that they can offer the tests to their sexual partners. | 15/1/2024 | Included | HIVST; focuses on index testing |
| 45 | Maierhofer CN et al., 2023, Malawi | To characterize heterogeneity in effectiveness of network-based HIV testing interventions vs passive PN among persons with HIV. | 15/1/2024 | Included | APN included via network-based partner notification |
| 46 | Ganju SA & Kanga AK, 2018, India | To describe a client partner notification and management approach aimed at reducing new HIV infections. | 16/1/2024 | Excluded | No full-text article; Conference abstract only |
| 47 | Odhiambo F et al., 2020, Kenya | To evaluate the impact of assisted partner notification services on closing the HIV identification gap for men in a real-world setting in Kenya. | 16/1/2024 | Excluded | No full-text article; Conference abstract only |
| 48 | Thirumurthy H et al., 2020, Kenya | To determine whether an HIV self-testing intervention promotes partner testing and safer sexual behavior among women at high risk of HIV infection in a cluster randomized trial in Kenya. | 16/1/2024 | Excluded | Double Duplicate |
| 49 | Krishnan K et al., 2020, India | To assess whether combining an enhanced peer outreach approach with index testing is an effective strategy for reaching key populations at high risk in HIV concentrated settings in India. | 17/1/2024 | Excluded | No full-text article; Conference abstract only |
| 50 | Nguyen V et al., 2019, Vietnam | To explore the feasibility and effectiveness of implementing assisted partner notification (aPN) as part of community testing services for key populations. | 17/1/2024 | Included | APN used among key populations (FSW, MSM, PWID); |
| 51 | Onovo A et al., 2021, Nigeria | To describe feasibility and effectiveness of community-led index case testing for HIV diagnosis & linkage among key population (KP) partners. | 17/1/2024 | Included | APN used among key populations (MSM, FSW, PWID)-Nigeria |
| 52 | Nguyen V et al., 2019, Vietnam | To document lessons from a pilot study on community-led HIV testing services, including HIV self-testing and assisted partner notification services, in a concentrated epidemic setting in Vietnam. | 18/1/2024 | Excluded | Double Duplicate |
| 53 | Mokgatle M et al., 2021, South Africa | To compare risky sexual behaviors, self-reported STIs, knowledge of symptoms, and partner notification practices among male and female university students in Pretoria, South Africa. | 18/1/2024 | Excluded | Wrong outcome; STI behavior focus, not HIVST or index testing. |
| 54 | Obionu I et al., 2021, Nigeria | To compare patterns of HIV status disclosure among people living with HIV in peer support versus non-support groups in Enugu, Nigeria. | 18/1/2024 | Excluded | Wrong outcome; disclosure patterns, not HIVST or index testing. |
| 55 | Shamu S et al., 2019, South Africa | To compare index client tracing modality’s outcomes with other community-based HIV counseling and testing (CBCT) recruitment modalities (mobile, workplace, home-based) during 2015-2017. | 19/1/2024 | Included | APN via index client tracing; setting: South Africa |
| 56 | Dvora L. et al., 2022, South Africa. | To examine how HIVST can be incorporated as part of index partner testing in the rural South African context. | 19/1/2024 | Included | APN using client, provider, and contract referral in Rwanda |
| 57 | Owoso K et al., 2019, Nigeria | To evaluate contact tracing of HIV by assessing sexual partners, spouses, and children of newly diagnosed patients through a referral method in Nigeria. | 19/1/2024 | Excluded | No full-text article; Conference abstract only |
| 58 | Wamuti B et al., 2022, Kenya | We estimated the costs of integrating assisted partner services (aPS) into routine HIV testing services (HTS) within an ongoing aPS scale-up project in western Kenya. | 20/1/2024 | Included | APN among newly diagnosed HIV cases in Kenya |
| 59 | Offorjebe O et al., 2019, Malawi | To assess the cost-effectiveness and national impact of index HIV self-testing in Malawi. | 20/1/2024 | Excluded | No full-text article; Conference abstract only |
| 60 | Sharma M et al., 2016, Kenya | To evaluate the cost-effectiveness of implementing assisted partner notification for HIV in Kenya using a mathematical modeling approach. | 20/1/2024 | Excluded | Wrong outcome, wrong publication type |
| 61 | Mogaka J et al., 2023, Kenya | To describe the feasibility of integrating hypertension screening into an HIV assisted partner notification services model in Kenya. | 21/1/2024 | Excluded | Wrong outcome; wrong publication type; modelling only; thesis |
| 62 | Andriyanto A et al., 2023, Indonesia | To analyze the determinants of notification of spouses of people with HIV/AIDS (PLWHA) in Care, Support, and Treatment Services in Mojokerto City. | 21/1/2024 | Included | APN among PLHIV in care in Indonesia |
| 63 | Vermandere H et al., 2021, Mexico | To explore the awareness of and need for HIV partner notification, as well as to outline potential strategies for APNS based on identified barriers and facilitators. | 21/1/2024 | Included | APN development study focused on key populations in Mexico |
| 64 | Onovo A et al., 2022, Nigeria | To describe the implementation of index partner testing as part of the national KP program and to present the first results. | 22/1/2024 | Included | Community-based APN among key populations in Nigeria |
| 65 | Katbi M et al., 2018, Nigeria | To design and evaluate the impact of interventions aimed at identifying previously undiagnosed cases of HIV infections among the sexual partners of index persons living with HIV (PLHIV). | 22/1/2024 | Included | Index testing among newly diagnosed HIV clients in Nigeria |
| 66 | Oldenburg CE et al., 2018, Zambia | To assess the effect of two health system approaches to distribute HIV self-tests on the number of female sex workers client and nonclient sexual partners. | 22/1/2024 | Included | HIVST secondary distribution FSW in Zambia |
| 67 | Dovel K et al., 2023, Malawi | To assess the impact of index HIV self-testing (HIVST) on testing uptake among ART clients primary sexual partners as compared to the standard of care partner referral slips (PRS) and describe ART initiation among diagnosed individuals. | 23/1/2024 | Included | Index HIVST among ART clients in Malawi |
| 68 | Thirumurthy H et al., 2021, Kenya | To examine whether sustained provision of self-tests to women promotes testing among sexual partners and reduces HIV risk. | 23/1/2024 | Included | HIVST via secondary distribution |
| 69 | Ye ZH et al., 2023, China | To assess how an intervention (aPS with CBOs and HIV self-testing, aPSST) affects HIV partner testing in newly and previously diagnosed MSM, and its impact on identifying new testers, positives, linkage to care, and safety. | 23/1/2024 | Included | Community-led APN with HIVST among HIV-positive MSM in China |
| 70 | Marwa T et al., 2019, Kenya | To evaluate the effects of HIV self-testing kits on increasing uptake of male partner testing among pregnant women attending antenatal clinics in Kenya in a randomized controlled trial. | 24/1/2024 | Excluded | Wrong population: ANC clients were HIV-negative, not PLHIV or key populations. |
| 71 | Tembo TA et al., 2019, Malawi | To evaluate the impact of a behavioral skills-building training for healthcare providers on enhancing an HIV index case testing passive referral model in Mangochi District, Malawi. | 24/1/2024 | Excluded | Wrong outcome; provider training, not HIVST or aPN. |
| 72 | Torbunde N et al., 2021, Nigeria | To evaluate family index testing among biological children of people living with HIV in Nigeria. | 24/1/2024 | Excluded | Wrong population; child testing focus, not adult >18 years old PLHIV or KP. |
| 73 | Makyao N et al., 2020, Zambia | There is an opportunity to expand HIV identification from deceased HIV clients, and we recommend scaling up testing of contacts of deceased clients to reach potential undiagnosed individuals. | 25/1/2024 | Excluded | No full-text article; Conference abstract only |
| 74 | Clark JL et al., 2017, Peru | We assessed the impact of Expedited Partner Therapy (EPT) on self-reported partner notification among MSM in Peru with gonorrheal and/or chlamydial infection. | 25/1/2024 | Excluded | Wrong outcome; STI treatment focus, not HIVST or index testing. |
| 75 | Culbert GJ et al., 2020, Indonesia | To assess how the Impart APN model affects partner notification and HIV testing outcomes for incarcerated men with HIV; evaluate feasibility and effectiveness of implementing APN programs in prisons. | 25/1/2024 | Included | Explores acceptability of partner notification; Indonesia |
| 76 | Yan XM et al., 2022, China | To examine facilitators and barriers of HIV partner services (PS) using qualitative analysis. | 26/1/2024 | Included | HIV partner notification among MSM in China |
| 77 | Rahmalia A et al., 2022, Indonesia | To investigate HIV status disclosure and partner testing practices among women living with HIV (WLWH) in urban Bandung, Indonesia, to identify needs in partner notification services. | 26/1/2024 | Included | Self-Disclosure & PN, Disclosure women PLHIV |
| 78 | Gitige CG et al., 2021, Tanzania | To determine predictors of partner elicitation among index HIV-positive clients. | 26/1/2024 | Included | Index testing factors, Tanzania |
| 79 | Simon KR et al., 2018, Unknown | Family testing is an index case finding strategy through which HIV-infected patients are systematically screened to identify family members with unknown HIV status. | 27/1/2024 | Excluded | Wrong population: pediatric focus not not adult >18 years old PLHIV, not partner testing. |
| 80 | Asamoah Ampofo E et al., 2022, Ghana | To explore acceptance, barriers, challenges, and facilitators of family-based index testing for HIV among clients in Cape Coast, Ghana. | 27/1/2024 | Excluded | Wrong outcome; index testing without HIVST or aPN. |
| 81 | Mogaka JN et al., 2023, Kenya | To assess the feasibility of integrating hypertension screening into an HIV assisted partner notification services model in Kenya. | 27/1/2024 | Excluded | Wrong outcome; focus on HTN screening, not HIVST or aPN. |
| 82 | Martin K et al., 2023, Zimbabwe | This protocol outlines a cluster randomised trial to assess whether financial incentives improve uptake of partner services for STIs in antenatal care settings in Zimbabwe. | 28/1/2024 | Excluded | Wrong outcome; STI partner services protocol, not HIV |
| 83 | Sharma M et al., 2021, Kenya | To assess aPS acceptability, reasons, and predictors of non-enrolment among females in an ongoing implementation project of aPS scale-up in western Kenya, a region with high HIV prevalence (15%). | 28/1/2024 | Included | APN among diagnosed PLHIV; Kenya. |
| 84 | Davey DJ et al., 2022, South Africa | To evaluate the uptake and outcomes of HIV self-testing among male partners of women living with HIV in rural South Africa. | 28/1/2024 | Excluded | No full-text article; Conference abstract only |
| 85 | Goyette MS et al., 2018, Kenya | To investigate whether the history of intimate partner violence (IPV) modified APS effectiveness and risk of relationship dissolution. | 29/1/2024 | Included | APNS; diagnosed PLHIV |
| 86 | Songane M et al., 2023, Mozambique | To estimate the cost-efficiency and effectiveness of community index testing and compare the HIV testing outputs with facility-based testing. | 29/1/2024 | Included | HIV community index testing; Mozambique |
| 87 | Dessalegn NG et al., 2019, Ethiopia | To examine HIV-positive status disclosure to sexual partners among individuals receiving HIV care in Addis Ababa, Ethiopia. | 29/1/2024 | Excluded | Wrong outcome; disclosure focus, not HIVST or index testing. |
| 88 | Gibson W et al., 2016, Unknown | To pilot and evaluate the feasibility, acceptability, and outcomes of home/self-testing for HIV. | 30/1/2024 | Excluded | Wrong outcome; general HIVST use, not index testing or aPN. |
| 89 | Cibangu K, 2022, Zambia | To review existing medical files and registers in the Matero subdistrict of Zambia to describe existing information on index testing and propose better ways to improve HIV index testing positivity yield. | 30/1/2024 | Included | HIV index partner testing; Zambia |
| 90 | Jubilee M et al., 2019, Lesotho | To evaluate whether HIV index testing improves positivity rates and linkage to care among sexual partners, adolescents, and children of PLHIV in Lesotho. | 30/1/2024 | Excluded | Wrong population; primarily pediatric/adolescent focus. |
| 91 | Korte JE et al., 2020, Uganda | To determine uptake of HIV oral self-testing and linkage to care among male partners of women attending ANC in Central Uganda in a randomized trial. | 31/1/2024 | Excluded | Wrong population; HIV-negative ANC women |
| 92 | Cheng W et al., 2019, China | To assess partner notification patterns across different sexual partner types among MSM diagnosed with HIV in Guangzhou, China. | 31/1/2024 | Excluded | Wrong publication type; Letter to editor |
| 93 | Sheriff V, 2023, Unknown | To audit new HIV diagnoses and partner notification activities during the COVID-19 pandemic at a local clinic between January 2020 and February 2022. | 31/1/2024 | Excluded | No full-text article; Conference abstract only |
| 94 | Gore D et al., 2018, Unknown | To describe the availability and potential benefits of HIV partner notification services among young Black MSM in a representative sample. | 1/2/2024 | Excluded | Wrong setting; USA (high-income country). |
| 95 | Quinn C et al., 2018, Uganda | To understand community reactions to both passive and assisted partner notification approaches, with a specific focus on comparing responses between communities with differing HIV risk. | 1/2/2024 | Included | HIV partner notification; Uganda |
| 96 | Payne C et al., 2017, Uganda | To explore values and preferences regarding HIV partner notification among sex workers, fishermen, and community members in Rakai, Uganda. | 1/2/2024 | Excluded | No full-text article; Conference abstract only |
| 97 | Cherutich P et al., 2018, Kenya | To conduct cost and budget impact analyses, respectively, of aPS compared to the current practice of HIV testing services (HTS) in Kisumu County, Kenya. | 2/2/2024 | Included | HIV partner services in Kenya |
| 98 | Sanga E et al., 2023, Tanzania | To explore decision-making around disclosure to sexual partners among PLHIV on ART in North-Western Tanzania. | 2/2/2024 | Included | Sexual Partners, Disclosure, Self Disclosure; Tanzania |
| 99 | Choko AT et al., 2019, Malawi | To evaluate HIV self-testing alone or combined with additional interventions (e.g., financial incentives) and their effect on linkage to care or prevention among male partners of ANC attendees in Malawi. | 2/2/2024 | Excluded | Wrong population; HIV-negative ANC clients. |
| 100 | Mujugira A et al., 2021, Uganda | To explore how HIV self-testing and oral PrEP empower sex workers and their intimate partners in Uganda. | 3/2/2024 | Excluded | Wrong outcome: HIV prevention focus (not partner notification or index testing). |
| 101 | Agot K et al., 2020, Kenya | To explore the experiences of women distributing HIV self-test (HIVST) kits to their sexual partners, how partners reacted to self-testing and results, and how their relationships were affected by test results. | 3/2/2024 | Included | HIVST; HIV-Positive Women and their Sexual Partners; Kenya |
| 102 | Odiachi A et al., 2018, Nigeria | To investigate HIV status disclosure to male partners among rural Nigerian women in the PMTCT cascade through a mixed methods approach. | 3/2/2024 | Excluded | Double Duplicate |
| 103 | Olakunde BO et al., 2018, Nigeria | To examine HIV testing uptake among male partners of pregnant women in Nigeria and identify barriers. | 4/2/2024 | Excluded | Wrong population; HIV-negative ANC clients. |
| 104 | Luo MY et al., 2020, China | To analyze the uptake and infection status of HIV testing for sexual partners of newly diagnosed HIV-positive MSM. | 4/2/2024 | Included | index testing with HIVST among MSM in China |
| 105 | Kiene SM et al., 2017, Uganda | To assess the perceived feasibility and acceptability of index partner HIV self-testing (HIVST) by HIV-positive clients versus partner referral slips (standard of care) among HIV-positive clients in Uganda. | 4/2/2024 | Included | PITC study on partner testing uptake; includes PN outcomes and predictors in Uganda. |
| 106 | Matovu JKB et al., 2018, Uganda | To explore perceptions, delivery strategies, and post-test experiences of HIV self-testing among pregnant women and their male partners in Central Uganda. | 5/2/2024 | Excluded | Wrong population; HIV-negative ANC clients. |
| 107 | Naughton B et al., 2023, Uganda | To explore relationship factors impacting acceptability of HIV self-testing among pregnant women and their male partners in Uganda. | 5/2/2024 | Excluded | Wrong population; HIV-negative ANC clients. |
| 108 | Matovu JK et al., 2017, Uganda | To examine pregnant women and male partners' perceptions of female partner-delivered HIV self-testing in Uganda. | 5/2/2024 | Excluded | Wrong population; HIV-negative ANC clients. |
| 109 | Gichangi A et al., 2018, Kenya | To measure the impact of distributing HIV self-test kits to male partners of antenatal care (ANC) clients on testing uptake in Kenya. | 6/2/2024 | Excluded | Wrong population; HIV-negative ANC clients. |
| 110 | Culbert GJ et al., 2023, Indonesia | To examine the willingness of people living with HIV (PLHIV) in prison to participate in assisted HIV partner notification services and to explore their reasons for or against disclosing their HIV-positive status to their partners. | 6/2/2024 | Included | PN, testing, and new diagnoses; Indonesia |
| 111 | Little KM et al., 2019, Central Asia | To document implementation experiences and lessons learned from scaling up an HIV assisted partner notification intervention in Central Asia. | 6/2/2024 | Excluded | Wrong outcome; lacks relevant index testing or PNS outcomes. |
| 112 | Wamuti B et al., 2023, Kenya | To explore the factors affecting implementation fidelity to assisted partner services (aPS) in two high-HIV prevalence counties in western Kenya. | 7/2/2024 | Included | aPS fidelity study in Kenya |
| 113 | Mwakangalu D et al., 2016, Kenya | To implement and evaluate a quality improvement initiative aimed at increasing male partner testing in PMTCT settings at Kinango Hospital, Kwale County, Kenya. | 7/2/2024 | Excluded | Wrong outcome; no HIVST or index testing focus. |
| 114 | Matoga MM et al., 2018, Malawi | To apply the Model for Improvement to enhance passive partner notification for STI and HIV case finding in Lilongwe, Malawi. | 7/2/2024 | Excluded | No full-text article; Conference abstract only |
| 115 | Matoga M, 2018, Malawi | To assess the effect of quality improvement methods on passive partner notification for STI/HIV in Malawi. | 8/2/2024 | Excluded | Wrong publication; Thesis |
| 116 | Muchedzi A et al., 2018, Zimbabwe | To evaluate the technical efficiency of community index HIV sexual network testing for reaching the first 90 in Zimbabwe. | 8/2/2024 | Excluded | No full-text article; Conference abstract only |
| 117 | Mwango LK et al., 2020, Zambia | To present the CIRCUITS approach to case finding and examine HIV positivity yield and ART linkage across index testing and targeted community testing modalities, with sub-analyses by sex, age groups, and district. | 8/2/2024 | Included | Index testing, CBCT, men, Zambia, ART linkage |
| 118 | Urasa PL et al., 2019, Tanzania | To assess the effectiveness of index case finding strategies in closing gaps in HIV diagnosis in Tanzania. | 9/2/2024 | Excluded | No full-text article; Conference abstract only |
| 119 | Guthrie B et al., 2020, Kenya | To identify index factors associated with increased partner notification yield among people who inject drugs in Kenya. | 9/2/2024 | Excluded | No full-text article; Conference abstract only |
| 120 | Masyuko SJ et al., 2019, Kenya | To assess the efficacy of assisted partner services (aPS) based on the characteristics of index participants, including region of residence, rural/peri-urban vs. urban location, gender, age, and knowledge of HIV status. | 9/2/2024 | Included | RCT, aPS, index characteristics, Kenya, PLHIV, HIV testing, case finding |
| 121 | Francois VA, 2017, Haiti | To evaluate index partner testing and targeted case finding approaches for identifying HIV infections in northern Haiti. | 10/2/2024 | Excluded | No full-text article; Conference abstract only |
| 122 | Altaf A et al., 2021, Pakistan | To determine the feasibility and success of index testing among HIV-positive patients for identifying new HIV infections in Pakistan. | 10/2/2024 | Excluded | Wrong population; parent-child household testing |
| 123 | Remera E, 2019, Rwanda | To compare the efficiency of index testing versus intensified case finding approaches for HIV testing in Rwanda. | 10/2/2024 | Excluded | Wrong publication type; Letter to editor |
| 124 | Enugu A et al., 2022, Unknown | To evaluate various index testing approaches for early diagnosis of PLHIV and treatment initiation to support HIV epidemic control. | 11/2/2024 | Excluded | Wrong publication type; Letter to editor |
| 125 | Legkostup L et al., 2021, Ukraine | To assess performance metrics of index testing implementation efforts in Ukraine during 2019. | 11/2/2024 | Excluded | Wrong publication type; Letter to editor |
| 126 | Malik M et al., 2019, Pakistan | To evaluate the integration of assisted partner notification into existing HIV prevention services for people who inject drugs in Pakistan. | 11/2/2024 | Excluded | Wrong publication type; Letter to editor |
| 127 | Wamuti BM et al., 2023, Kenya | To explore stakeholders perspectives on integrating assisted partner services into Kenyas' national HIV testing services program. | 12/2/2024 | Excluded | Wrong outcome: focuses on implementation process, not index testing outcomes. |
| 128 | Grande M et al., 2021, Botswana | To evaluate Botswana's assisted partner services (APS) for reach, effectiveness, APS value for known cases, and re-linking identified partners to care. | 12/2/2024 | Included | aPS, implementation study, partner elicitation, low HIV case-finding, Botswana |
| 129 | MacPherson P et al., 2019, South Africa | To compare intensified household contact tracing and support versus standard of care for tuberculosis case contacts in a South African setting. | 12/2/2024 | Excluded | Wrong outcome; TB-focused study, not HIV index testing. |
| 130 | Mokgatle MM et al., 2018, South Africa | To assess intentions to use patient-initiated partner notification and acceptability of provider-initiated partner notification for STIs among minibus taxi drivers in Gauteng Province, South Africa. | 13/2/2024 | Excluded | Wrong outcome; STI partner notification, not HIV-specific. |
| 131 | Stangl AL et al., 2020, Multiple LMIC | To systematically review the safety of HIV index testing and partner notification for adolescent girls and young women in low- and middle-income countries. | 13/2/2024 | Excluded | Wrong publication type; commentary (no primary data). |
| 132 | Liu W et al., 2022, Kenya | To explore the experience of providing assisted partner services (APS), the barriers and facilitators, and how contextual factors influenced their experience. | 13/2/2024 | Included | aPS; provider acceptability; challenges/facilitators; Kenya; qualitative study |
| 133 | Lariat J et al., 2023, Zimbabwe | To investigate the acceptability of patient-referral partner notification for sexually transmitted infections among young people in Zimbabwe, identifying facilitators and barriers through a mixed methods approach. | 14/2/2024 | Excluded | Wrong outcome; STI partner notification, not HIV-specific. |
| 134 | Lariat J et al., 2023, Zimbabwe | To explore young people's perceptions of patient-referral partner notification for sexually transmitted infections and identify associated facilitators and barriers in Zimbabwe. | 14/2/2024 | Excluded | Double Duplicate |
| 135 | Lakoh S et al., 2019, Sierra Leone | To determine the rate of partner testing and identify factors associated with low partner testing in a high HIV prevalence setting in Freetown, Sierra Leone. | 14/2/2024 | Excluded | Wrong population-targeted general ANC mother not focused on PLHIV or key populations. |
| 136 | Guta K et al., 2022, Ethiopia | To measure the magnitude of prompt HIV-seropositive status disclosure to partners and identify associated factors among adult ART clients at Holeta Health Center in central Ethiopia, 2020. | 15/2/2024 | Excluded | Wrong outcome; focuses on disclosure timing, not partner testing or notification. |
| 137 | Alemayehu MT et al., 2017, Ethiopia | To assess the extent of male involvement in PMTCT and associated factors related to partner testing among couples in Goba town, Ethiopia. | 15/2/2024 | Excluded | Wrong population; ANC clients. |
| 138 | Krakowiak D et al., 2016, Unknown | To compare the effectiveness of home-based male partner HIV testing versus clinic invitation on male partner testing uptake during pregnancy. | 15/2/2024 | Excluded | Wrong population; ANC clients. |
| 139 | Oyugi E et al., 2017, Kenya | To evaluate the level and determinants of male partner involvement in PMTCT efforts among couples in Kisumu County, Western Kenya. | 16/2/2024 | Excluded | Wrong population; ANC clients. |
| 140 | Haile C et al., 2021, Ethiopia | To determine the level of male partner involvement in HIV testing and counselling among partners of pregnant women in Delanta District, Ethiopia, and associated factors. | 16/2/2024 | Excluded | Wrong population; ANC clients. |
| 141 | Napierala S et al., 2020, Kenya | To assess male partner testing uptake and sexual behavior changes after providing multiple HIV self-test kits to high-risk Kenyan women in a cluster randomized trial. | 16/2/2024 | Excluded | Wrong population; HIV-negative wome |
| 142 | Gottert A et al., 2018, Uganda | To explore relationship dynamics and HIV testing behavior among male partners of young women in Uganda. | 17/2/2024 | Excluded | Wrong population; male partners of AGYW |
| 143 | Wamuti B et al., 2020, Kenya | To identify factors associated with nonenrolment of HIV-positive female index clients into partner notification services in Kenya. | 17/2/2024 | Excluded | No full article; conference abstract. |
| 144 | Afe AJ et al., 2021, Nigeria | To assess the outcome of implementing Partner HIV Testing Services (PNS) in three public secondary health facilities in the north-central region of Nigeria. | 17/2/2024 | Included | PNS; HIV outcomes; PLHIV; Nigeria; case-finding study |
| 145 | Lasry A et al., 2021, Cote d'Ivoire | To evaluate HIV testing outcomes among family members of index cases across 36 health facilities in Abidjan, Cote d'Ivoire. | 18/2/2024 | Excluded | Wrong population: focused on family/child testing, not partner notification |
| 146 | Mgaya R et al., 2022, Tanzania | To assess outcomes of partner elicitation HIV testing services among key vulnerable populations via mobile laboratories in Mbeya and Songwe regions, Tanzania. | 18/2/2024 | Excluded | No full article; conference abstract. |
| 147 | Lyamuya FS et al., 2020, Tanzania | To examine outcomes and associated factors of partner notification and HIV testing processes in Northeastern Tanzania. | 18/2/2024 | Excluded | Wrong publication; udner review |
| 148 | Braun HM et al., 2017, Peru | To analyze individual and partnership factors associated with anticipated versus actual partner notification following STI diagnosis among MSM and transgender women in Lima, Peru. | 19/2/2024 | Excluded | Wrong outcome; STI partner notification, not HIV-specific. |
| 149 | Yan X et al., 2021, Unknown | To develop a people-centered approach for HIV partner notification intervention packages by identifying facilitators and barriers using a socioecological framework. | 19/2/2024 | Excluded | No full article; conference abstract. |
| 150 | Benemariya N et al., 2023, Rwanda | To assess factors associated with partner notification among people living with HIV in Bushenge Hospital, 2018â€“2019. | 19/2/2024 | Included | PNS; PLHIV; Rwanda; factors influencing notification |
| 151 | Harry TC et al., 2019, Nigeria | To argue for the necessity of integrating partner notification and contact tracing with provider-initiated HIV counselling and testing during population screening in Nigeria. | 20/2/2024 | Excluded | Wrong publication type; commentary (no primary data). |
| 152 | Green H et al., 2020, South Africa | To evaluate partner notification and treatment outcomes for sexually transmitted infections among pregnant women in Cape Town, South Africa. | 20/2/2024 | Excluded | Wrong outcome; STI partner notification, not HIV-specific. |
| 153 | Offorjebe OA et al., 2017, Botswana | To assess partner notification and treatment outcomes for STIs among pregnant women in Gaborone, Botswana. | 20/2/2024 | Excluded | Wrong outcome; STI partner notification, not HIV-specific. |
| 154 | Chitneni P et al., 2020, South Africa | To assess partner notification and treatment outcomes among South African adolescents and young adults diagnosed with an STI via laboratory-based screening. | 21/2/2024 | Excluded | Wrong outcome; STI partner notification, not HIV-specific. |
| 155 | Kingbo MH et al., 2020, Cote d'Ivoire | To compare partner notification approaches for sex partners and children of HIV index cases in Côte d’Ivoire (Ivory Coast). | 21/2/2024 | Excluded | Wrong population; includes child testing. |
| 156 | Cavalcante EG et al., 2016, Unknown | To explore perceptions of notified partners following partner notification for sexually transmitted infections. | 21/2/2024 | Excluded | Wrong outcome; STI partner notification, not HIV-specific. |
| 157 | Fu X et al., 2016, China | To explore the feasibility and efficiency of sexual partner notification (PN) and HIV testing among HIV-positive MSM in cooperation with MSM-serving CBOs via a pilot study in two Chinese cities. | 22/2/2024 | Included | PNS; MSM; China; CBO involvement; HIV+ index cases; testing outcomes |
| 158 | Tih PM et al., 2020, Cameroon | To evaluate partner notification practices among Cameroonian MSM diagnosed with HIV. | 22/2/2024 | Excluded | Wrong publication type; Thesis |
| 159 | Ugbena ER et al., 2021, Nigeria | To describe the level of acceptability and outcome of PNS among HIV-positive key populations (KPs) using various PN approaches and to describe the proportion of HIV-positive KPs who accepted partner notification services after diagnosis. | 22/2/2024 | Included | PNS among HIV+ KP in Nigeria; |
| 160 | Maroko GM et al., 2019, Kenya | To evaluate the implementation and outcomes of partner notification services in Kisii County, Kenya. | 23/2/2024 | Excluded | Wrong outcome; focuses on communication |
| 161 | Jiang W et al., 2021, Kenya | To determine partner HIV testing uptake among pregnant women offered retesting in Kenya. | 23/2/2024 | Excluded | Wrong population-targted ANC mother |
| 162 | Drammeh B et al., 2020, Multiple | To describe partner testing services and their contribution to HIV epidemic control in nine PEPFAR countries in 2019. | 23/2/2024 | Excluded | No full article; conference abstract. |
| 163 | Mujugira A et al., 2023, Uganda | To evaluate the benefits and risks of secondary distribution of HIVST from PLHIV. | 24/2/2024 | Included | HIVST via secondary distribution by PWLHIV in Uganda |
| 164 | Mujugira A et al., 2022, Uganda | To examine the effectiveness of HIV self-test distribution by Ugandan pregnant women with HIV in increasing partner testing. | 24/2/2024 | Excluded | Double Duplicate |
| 165 | Choko AT et al., 2021, Malawi | To investigate whether secondary administration of HIVST kits, with or without an additional financial incentive, via women receiving antenatal care (ANC) or via people newly diagnosed with HIV (i.e., index patients) could improve the proportion of male partners tested or the number of people newly diagnosed with HIV. | 24/2/2024 | Included | Index HIVST via ANC and newly diagnosed PLHIV; Malawi |
| 166 | Zhang K et al., 2019, China | To describe the partner notification (PN) experiences of people living with HIV (PLWH) and explore the perceived facilitators and barriers to PN using a qualitative method. | 25/2/2024 | Included | PN barriers and facilitators among PLHIV in China |
| 167 | Hershow RB et al., 2019, Malawi; Zambia | To assess stakeholders views and preferences of partner notification, home-based testing, and secondary distribution of self-test kits to understand whether offering choices for partner HIV testing may increase acceptability. | 25/2/2024 | Included | HIVST + PN perspectives; Malawi/Zambia |
| 168 | Setia GB et al., 2021, Indonesia | To understand provider and patient perspectives on assisted partner notification (aPN) from three demonstration sites in cities with a high HIV burden. | 25/2/2024 | Included | aPN acceptability among PLHIV, providers, Indonesia |
| 169 | Tembo TA et al., 2021, Malawi | To pilot-test a blended learning package for health care workers to improve index testing services in Southern Malawi. | 26/2/2024 | Excluded | Wrong population; focuses on healthcare workers, |
| 170 | Rees J et al., 2018, Unknown | To audit and evaluate partner notification processes at a community sexual health clinic for individuals newly diagnosed with HIV. | 26/2/2024 | Excluded | No full article; conference abstract. |
| 171 | Emeh A et al., 2021, Nigeria | To determine the positivity yield and identify factors influencing the yield from index testing strategy in selected healthcare facilities in Ondo State, southwest Nigeria. | 26/2/2024 | Included | Index testing study with positivity yield and linkage outcomes in Nigeria. |
| 172 | Masters SH et al., 2016, Malawi | To determine whether secondary distribution of multiple HIV self-tests by antenatal/postpartum women increases partner and couples testing versus invitation cards. | 27/2/2024 | Included | HIVST via secondary distribution by ANC |
| 173 | Cassell J et al., 2018, Unknown | To review public health aspects of STIs, focusing on partner notification strategies. | 27/2/2024 | Excluded | Wrong outcome; general STI PN review, not HIV-specific |
| 174 | Maman S et al., 2017, Kenya | To explore factors shaping the decisions of female sex workers (FSW) to offer self-tests to some of their partners, the strategies they used to introduce self-tests, and the reactions they received from their partners. | 27/2/2024 | Included | HIVST via FSW-led secondary distribution; Kenya |
| 175 | Dovel KL et al., 2022, Malawi | To evaluate the impact of index HIV self-testing on testing uptake among sexual partners of ART clients in Malawi through a randomized trial. | 28/2/2024 | Excluded | Double Duplicate |
| 176 | Krakowiak D et al., 2020, Kenya | To explore how home-based couple HIV testing among pregnant women and their male partners can reach hard-to-reach men in western Kenya. | 28/2/2024 | Excluded | Wrong outcome-general experiences of HIV-positive couples |
| 177 | Buhikire K et al., 2018, Uganda | To find out the predictors of successful contact tracing and testing of partners of HIV-positive individuals and possible barriers to contact. | 28/2/2024 | Included | APN study in Uganda |
| 178 | Mwango LK et al., 2019, Zambia | To present early results on the reach and yield of index testing among unreachable populations in Zambia's CIRKUITS project. | 29/2/2024 | Excluded | No full article; conference abstract. |
| 179 | Golden MR et al., 2020, Botswana | To assess the scale-up, outcomes, and challenges of assisted partner services (APS) in Botswana. | 29/2/2024 | Excluded | No full article; conference abstract. |
| 180 | Joel JN et al., 2022, Kenya | To describe the implementation strategies of the index testing program in Nairobi County, Kenya, and assess outcomes along the HIV index testing cascade over the first two years of implementation. | 29/2/2024 | Included | Index testing; Kenya |
| 181 | Joel JN et al., 2023, Kenya | To document experiences and achievements from scaling up HIV index testing in Nairobi County, Kenya. | 1/3/2024 | Excluded | Double Duplicate |
| 182 | Masyuko S et al., 2020, Kenya | To evaluate the implementation and outcomes of scaling up assisted partner notification services in Western Kenya. | 1/3/2024 | Excluded | No full article; conference abstract. |
| 183 | Omare JN et al., 2016, Kenya | To examine the process and outcomes of scaling up partner testing in maternal and child health clinic settings at Gucha Sub-County Hospital, Western Kenya. | 1/3/2024 | Excluded | Wrong population – focused on ANC mothers. |
| 184 | Zishiri V et al., 2022, South Africa | To evaluate the programmatic implementation of partner-delivered self-testing through antenatal care (ANC) attendees and people newly diagnosed with HIV by assessing use, positivity, linkage, and cost per kit distributed. | 2/3/2024 | Included | HIVST via secondary distribution by ANC and index clients in South Africa |
| 185 | Agot K et al., 2018, Kenya | To analyze the effect of secondary distribution of HIV self-tests on testing uptake among male partners of young women as a subgroup analysis of a randomized trial. | 2/3/2024 | Excluded | Wrong population – focused on HIV negative ANC mothers. |
| 186 | Sibanda EL et al., 2021, Unknown | To determine whether the secondary distribution of HIV self-test kits increases male partner testing uptake. | 2/3/2024 | Excluded | Wrong publication type; commentary. |
| 187 | Xiao WJ et al., 2020, China | To examine patterns and correlates of HIV self-testing (HIVST) distribution within Chinese MSM sexual network. | 3/3/2024 | Included | HIVST via sexual network among MSM in China |
| 188 | Chiou PY et al., 2022, China | To examine the feasibility and outcomes of using social networking platforms for sexual partner referral for HIV testing. | 3/3/2024 | Excluded | Wrong setting/country; study conducted in Taiwan. |
| 189 | Nguyen N et al., 2019, South Africa | To perform a latent class analysis to examine sexual partner types and incident HIV infection risk among rural South African adolescent girls and young women in HPTN 068. | 3/3/2024 | Excluded | Wrong outcome-Not index testing |
| 190 | Venter F et al., 2017, South Africa | To review policy and guidance considerations for HIV self-testing in South Africa. | 4/3/2024 | Excluded | Wrong outcome; focused on policy |
| 191 | Kundy J et al., 2022, Tanzania | To describe experiences and outcomes of successful index testing services at Amref Afya Kamilifu-supported sites in Tanga and Zanzibar. | 4/3/2024 | Excluded | No full article; conference abstract. |
| 192 | Cherutich P et al., 2016, Kenya | To describe surveillance methods and outcomes of HIV assisted partner services using routine health information systems in Kenya. | 4/3/2024 | Excluded | Wrong population-general testing |
| 193 | Mahachi N et al., 2019, Zimbabwe | To describe the implementation of index testing and partner notification services (PNS) under the Zimbabwe HIV Care and Treatment (ZHCT) project and the resulting uptake, HIV positivity rate, and links to HIV treatment. | 5/3/2024 | Included | Index testing + PNS under ZHCT in Zimbabwe; |
| 194 | Nolte K et al., 2017, Unknown | To explore experiences of Black women in approaching and encouraging male partners to test for HIV. | 5/3/2024 | Excluded | Wrong setting-USA |
| 195 | Curran K et al., 2017, Tanzania | To compare success rates between men and women in referring sexual partners for HIV testing via partner notification in Tanzania. | 5/3/2024 | Excluded | No full article; conference abstract. |
| 196 | Zewdie K et al., 2022, Kenya | To evaluate the effectiveness of a focused partner HIVST strategy to stimulate the identification of partners of index PLHIV in public health HIV clinics for PrEP or ART programs. | 6/3/2024 | Included | Index HIVST among PLHIV in Kenya |
| 197 | Mugwanya K et al., 2021, Kenya | To evaluate the positivity yield and reach of targeted index partner HIV self-testing among high-risk HIV-uninfected contacts in Kenya. | 6/3/2024 | Excluded | Double Duplicate |
| 198 | Clark JL et al., 2018, Peru | To evaluate whether traditional and web-based partner notification technologies improve notification and testing outcomes following syphilis diagnosis among MSM in Lima, Peru. | 6/3/2024 | Excluded | Wrong outcome; focused on syphilis partner notification, not HIV. |
| 199 | Kumwenda A et al., 2023, Unknown | To compare two strategies (partner notification and partner HIV self-testing) and identify predictors of male partner HIV testing in antenatal settings. | 7/3/2024 | Excluded | Wrong population; focused on HIV-negative ANC |
| 200 | Goyette M et al., 2016, Kenya | To qualitatively explore the client, community, and health system barriers to the implementation of APS in Kenya within the cluster randomized trial. | 7/3/2024 | Included | APS scale-up in Kenya |
| 201 | Kalichman SC et al., 2021, Unknown | To explore unintended consequences of undisclosed HIV status to sexual partners in the context of undetectable = untransmutable (U=U). | 7/3/2024 | Excluded | Wrong outcome: focused on disclosure behavior and U=U implications, not partner testing or HIVST. |
| 202 | Semple SJ et al., 2018, Mexico | To assess the uptake and outcomes of a partner notification model among primarily provider referral among MSM and transgender women in Tijuana, Mexico, while identifying factors influencing the notification of sexual partners. | 8/3/2024 | Included | Uptake of PN model among MSM and TGW; Mexico |
| 203 | Nante RW et al., 2023, Uganda | To determine uptake and associated factors of assisted partner notification among HIV-positive adults with severe mental illness at a national referral hospital in Uganda. | 8/3/2024 | Excluded | No full-text article; Conference abstract only |
| 204 | Mark J et al., 2017, Kenya | To measure uptake of home-based syphilis and HIV testing among male partners of pregnant women in western Kenya. | 8/3/2024 | Excluded | Wrong outcome: focus is on syphilis |
| 205 | Remera E et al., 2022, Rwanda | To assess the factors related to the HIV-positive outcome among older people (aged 50+) through index testing in Rwanda. | 9/3/2024 | Included | Included: APN among PLHIV |
| 206 | Beltrami J et al., 2018, USA | To describe the usefulness of individual-level HIV surveillance data for initiating statewide HIV partner services in Hawaii and New Mexico. | 9/3/2024 | Excluded | Wrong publication type-case study |
| 207 | Kariithi E et al., 2021, Unknown | To outline a protocol for an implementation science study evaluating assisted partner services for HIV testing and treatment among males and their female sexual partners. | 9/3/2024 | Excluded | Wrong outcome-study protocol |
| 208 | Wynn A et al., 2019, Botswana | To explore partner notification practices for STIs in a high HIV prevalence context in Botswana through qualitative methods. | 10/3/2024 | Excluded | Wrong outcome; focused on curable STIs, not HIV. |
| 209 | Kachero MM et al., 2021, Ethiopia | To assess utilization of HIV test services among pregnant women’s partners and associated factors in selected sub-cities of Addis Ababa, Ethiopia. | 10/3/2024 | Excluded | Wrong population-focused on HIV unknown status. |
| 210 | Kariuki RM et al., 2020, Kenya | To support policymakers and health managers in improving the implementation of PNS services aimed at achieving increasing HIV case identification and coverage of care and treatment. | 10/3/2024 | Included | Uptake of PNS; Kenya |
| 211 | World Health Organization, 2016, Global | To summarize WHO recommendations and rationale for assisting people with HIV to notify their partners. | 11/3/2024 | Excluded | Wrong publication-guidelines |
| 212 | Chen et al., 2019, Malawi | To determine the impact of a combination intervention versus passive partner notification on the identification of persons with HIV infection and their sexual and social contacts in Malawi. | 11/3/2024 | Included | partner notification vs. passive PN., Malawi |
| 213 | Uma TH et al., 2023, Ethiopia | To assess the proportion and the factors associated with partner and family-based index case HIV testing in Woliso Town, Oromia, Ethiopia. | 11/3/2024 | Included | Index testing using regular RDTs; Ethiopia |
