# Supplementary material for: HIV self-testing and partner notification strategies for key populations in low- to upper-middle-income countries: A mixed-methods systematic review
Source: PLoS One. 2025 Dec 29;20(12):e0338639. doi: 10.1371/journal.pone.0338639 (PMC12747344; doi:10.1371/journal.pone.0338639)
Supplement: S5 Table — (DOCX) [file pone.0338639.s005.docx]

| **Table S5. Characteristics of included studies** | | | | | | | | | | | | | | |
| --- | --- | --- | --- | --- | --- | --- | --- | --- | --- | --- | --- | --- | --- | --- |
| **S.No** | **Author** | **Study aim** | **Method** | **Extractor(s)** | **Date of Extraction** | **Eligibility Confirmed** | **Type of test kits used** | **Methods/strategies for PNS and testing** | **HIV-positive index clients** | | **HIV uninfected/unknown status KPs (MSM/TG/FSWs).** | | **Feasibility of index testing for PNS** | **Barriers and facilitators for PNS.** |
|  |  |  |  |  |  |  |  |  | **Acceptability of index testing for PNS** | **The outcome of PNS and testing** | **Acceptability of PNS** | **The outcome of PNS and testing** |  |  |
| 1 | Maman S et al., 2017, Kenya. | To explore factors shaping the decisions of FSW to offer self-tests to some of their partners, the strategies they used to introduce self-tests, and the reactions they received from their partners. | ▪Study design: Qualitative observational cohort ▪Population: HIV-negative FSW. ▪Mean age 28.4 years. ▪Sample size: 18 | LK | 28/2/2024 | JH, GC, JL | HIVST index testing | Secondary distribution | Not assessed | Not assessed | ▪12/18 women accepted and distributed HIVST to their primary.  ▪16/18 shared with their secondary clients. | ▪50 partners (11 primary, 39 commercial) of FSWs reached with HIVST. ▪39 clients reached via secondary distribution (27 regular, 12 new). ▪14 clients tested as a couple, 25 tested alone (often with a woman present). | ▪Withholding sex, conditional on testing. ▪Decision based on partner's perceived maturity or importance. | ▪Barriers: Negative reactions from partners including verbal and sexual abuse.  ▪Facilitators: Creative strategies to increase test uptake. |
| 2 | Hershow RB et al., 2019, Zambia. | To assess stakeholders’ views and preferences of partner notification, home-based testing, and secondary distribution of self-test kits to understand whether offering choices for partner HIV testing may increase acceptability. | ▪Study type: Qualitative cross-sectional  ▪Population: HIV-positive pregnant women and male sexual partners, Healthcare workers and policymakers. ▪Mean age: HIV-positive women (29 years) ▪Sample size: 148 | LK | 25/2/2024 | JH, GC, JL | HIVST index testing | Secondary distribution  Provider-based testing  Home-Based Testing | ▪Partner notification is motivating for women, men, and healthcare workers. ▪Healthcare workers and policymakers find it acceptable, citing effectiveness. | ▪Testing uptake: 43% (9 out of 21 women) ▪Home-based testing: 14% (3 out of 21 women) ▪HIVST uptake: 38% (8 out of 21 women) | Not assessed | ▪Partner notification: 28% (11 out of 39 women) ▪Home-based testing: 36% (14 out of 39 women) ▪Secondary distribution of self-test kits: 36% (14 out of 39 women) | ▪Lost referral letters, illiteracy, and lack of motivation. ▪Mixed views on follow-up tracing's effectiveness and concerns over costs.  ▪Concerns about difficulties in test administration, result reading, and seeking treatment. | Barrier:  ▪Partner distrust in women's ability to administer self-tests noted. ▪Potential relationship conflict over privacy concerns.  Facilitator:  ▪Convenient and confidential approach for men.  ▪Women appreciated their active role in facilitating testing with self-test kits. |
| 3 | Dovel K et al., 2023, Malawi. | To assess the impact of index HIVST on testing uptake among ART clients’ primary sexual partners as compared to the standard of care partner referral slips (PRS) and describe ART initiation among diagnosed individuals. | ▪Study type: RCT.  ▪Trail Registered: ClinicalTrials.gov, NCT03271307, and Pan African Clinical Trials, PACTR201711002697316. ▪Population: HIV-positive male and female,  ▪Age ≥15 years old.  ▪Sample size: 365 | LK | 23/1/2024 | JH, GC, JL | HIVST index testing | Partner referral slip (pRS)  Direct HIVST distribution | ▪HISVT distributed through PRS 92%. ▪HIVST distribution through direct self-test 90%.  ▪98% were comfortable explaining the intervention; ▪98.3% of females and 98.1% of males were comfortable with the HIV self-test demo.  ▪Low psychological IPV: 4% PRS, 1.2% HIVST. | ▪Testing uptake: HIVST 71% and pRS 24%  ▪Partner elicitation rate: HIVST 11% and 4%. ▪Testing yield: HISVT led to a 167% increase in testing uptake and a 211 increase in partner elicitation. | Not assessed | Not assessed | ▪Cost-efficient intervention for new diagnoses. ▪Some partners need guidance, counselling, or mistrust results. ▪Some partners faced challenges with understanding (16%) and interpreting (10%) HIV self-test. ▪Cost per test is lower for partner referral slip ($0.84) than for HIV self-test ($2.34). ▪Cost per person aware of positive status is lower for HIV self-test ($16.06) than PRS ($19.35). | Not assessed |
| 4 | Mutale W, 2021 et al., Zambia. | To investigate a combination approach, using evidence-based strategies, to increase HIV testing in male partners of HIV-positive and HIV-negative pregnant women. | ▪Study type: RCT.  ▪Trail Registered: ClinicalTrials.gov (NCT04124536). ▪Population: HIV-positive women, and HIV-negative women.  Age: HIV-positive woman 26, age range 23–29 years. HIV-negative women 26, age range 23–30 years)  Sample size: 329 | LK | 4/1/2024 | JH, GC, JL | HIVST index testing | Client self-referral  Contract referral | Not assessed | Trial 1 (HIV-positive women) Intervention Group ▪81% follow-up ▪6% facility-based male partner testing  ▪77% reported any male partner testing  Control Group ▪92% follow-up ▪28% facility-based male partner testing.  ▪36% reported any male partner testing. | Not assessed | In Trial 2 (Undiagnosed women) Intervention Group ▪97% follow-up ▪3% of facility-based male partner testing ▪78% reported any male partner HIV testing. Control Group ▪93% follow-up ▪34% of facility-based male partner testing ▪55% reported any male partner HIV testing. | Not assessed | Not assessed |
| 5 | Thirumurthy H et al., 2021 et al., Kenya. | To examine whether sustained provision of self-tests to women promotes testing among sexual partners and reduces HIV risk. | Study type: RCT.  Trail Registered: ClinicalTrials.gov, NCT03135067. Population: HIV-negative FSW. Age: 18 ≥years.  Sample size: 2102 | LK | 23/1/2024 | JH, GC, JL | HIVST index testing | Facility-based HIVST Direct HIVST distribution | Not assessed | Not assessed | ▪The HIVST intervention promoted frequent partner and couples testing | ▪At 6 months, 87.5% in the intervention group vs. 48.3% in comparison completed partner HIV tests. ▪Intervention consistently had higher partner testing rates at 12, 18, and 24 months.  Recently couples testing was 50.7% in intervention vs. 27.4% in comparison. | Not assessed | Not assessed |
| 6 | Agot K et al., 2018, Kenya. | To assess the occurrence of intimate partner violence (IPV) among women who accept an intervention involving the receipt of multiple self-tests for distribution to their sexual partners. Further, the study aims to compare IPV levels following the intervention to baseline levels of IPV. | Study type: Quantitative observational cohort.  Population: HIV-negative FSW. Median age: 25 years  Sample size: 280 | LK | 14/1/2024 | JH, GC, JL | HIVST index testing | Secondary distribution | Not assessed | Not assessed | Not assessed | ▪91% of ANC participants, 86% of PPC participants, and 75% of FSW) distributed a self-test to their partner. | ▪Baseline IPV did not significantly affect self-test distribution to partners.  ▪IPV occurrence decreased at follow-up compared to baseline. | Barrier:  ▪Baseline IPV linked to higher follow-up IPV likelihood. |
| 7 | Myers RS et al., 2016, Mozambique | To assess the acceptability, effectiveness, and safety of APS in a large, urban clinic in Maputo. | Study type: Quantitative observational cohort. Study type: HIV-positive male and female. Median age: 29 years. Sample size: 485 | LK | 1/1/2024 | JH, GC, JL | Regular RDTs for index testing | APS using CHWs | ▪Total APS acceptances: 220 IPs (99%) | ▪Testing uptake: Contact referral 54%, CHWs-assisted IPs 56%. | ▪35% of index clients anticipated future partnerships with HIV-negative partners. | ▪85% of partners contacted by CHWs. ▪32% of partners newly diagnosed with HIV through APS. | ▪65% of index patients disclosed their status before APS. | Barrier:  ▪Fear of IPV and adverse events identified. |
| 8 | Xiao WJ et al., 2020, China. | To examine patterns and correlates of HIVST distribution within Chinese MSM’s sexual network. | Study type: Quantitative observational cohort. Population: HIV-negative MSM.  Age: ≥18 years, median age 35 years.  Sample size: 400 | LK | 3/3/2024 | JH, GC, JL | HIVST index testing | Secondary distributing | Not assessed | Not assessed | ▪79.2% reported being present while their partners conducted the tests.  ▪54.2% used the kits together with their sexual partners. | ▪40.7% of participants distributed HIV self-test kits to partners. ▪51.4% gave one kit, while 15.3% distributed three or more. ▪58.3% gave kits to primary partners. ▪27.8% to both primary and casual partners. | ▪19.4% of distributors reported partners couldn't conduct tests properly. ▪ The majority reported no errors, indicating the finger prick method was easy. ▪Participants who hadn't tested recently were less likely to distribute kits. ▪Participants who used kits before were more likely to distribute. | Not assessed |
| 9 | Boye S et al., 2021, West Africa. | To improve our understanding of the practices, limitations and issues related to the distribution of HIV self-tests to PLHIV so that they can offer the tests to their sexual partners. | Study type: Qualitative cross-sectional.  Population: HIV-positive males and females. Age: 21-55 years.  Population: Health professionals, including physicians, nurses, social workers, pharmacists, and peer educators.  Sample size: 51 | LK | 15/1/2024 | JH, GC, JL | HIVST index testing | Health professional-assisted HIVST kits distribution. | ▪Out of 37 proposals for HIVST, 28 (23 women and 5 men) were accepted by PLHIV, indicating high acceptance. | ▪Majority of participants (91% ANC, 86% PPC, 75% FSW) distributed self-tests to their partners. | Not assessed | Not assessed | ▪Consultations with HIV self-test provision lasted 10-30 minutes, with variability depending on health professionals' approaches. | Barriers: ▪Health pros hesitated if PLHIV hadn't been disclosed. ▪PLHIV is reluctant due to fear of disclosure. Facilitators ▪Health pros-initiated test discussions. ▪PLHIV accepted tests after disclosure. |
| 10 | Nguyen V T H et al., 2019, Vietnam. | To explore the feasibility and effectiveness of implementing aPN as part of community testing services for key populations. | Study type: Quantitative quasi-experimental. Trail registration not specified. Population: Undiagnosed MSM, FSW, PWID.  Sample size: 3978 | LK | 17/1/2024 | JH, GC, JL | HIVST index testing | Provider referral Passive referral  Dual referral | Not assessed | Not assessed | ▪Clients preferred provider referral due to privacy concerns.  ▪Using these methods, 56.4% (105/186) were successfully contacted and provided with HTS. | ▪aPN was offered to 207 HIV-positive clients, and 105 partners tested for HIV. ▪ The proportion diagnosed with HIV was higher in self-testing (7.3%) vs. lay provider testing (5.8%). ▪Confirmatory testing and ART initiation found for lay provider and self-testing, except for FSWs | ▪Provider referral preferred, indicating operational feasibility.  ▪Successful partner notification among MSM partners (83.8%). | Barriers: ▪ Nondisclosure of HIV status by PLHIV.  ▪ PLHIVs fear disclosure due to concerns about stigma, & rejection, or  Facilitator: ▪Confidentiality, convenience, and free services. ▪Social media and innovative approaches reached young key populations, especially men. |
| 11 | Onovo A et al., 2022, Nigeria. | To describe the implementation of index partner testing as part of the national KP program and to present the first results. | Study type: Cross-sectional, quantitative.  Population: HIV-positive FSW, MSM, PWID including their sexual and drug-injecting partners, aged ≥15 years. Sample size: Total 10,508 (FSW: 5,636, MSM: 2,805 and PWID: 2,067). | LK | 22/1/2024 | JH, GC, JL | Regular RDTs for index testing. | Provider referral Passive/client referral | ▪Overall: Total: 10,508 Acceptance rate: 30.0%.  ▪Total Partners Elicited: 8,989 ▪Index Partner Ratio: 1:2.9 | ▪FSW Sexual Partners: 76.1% tested and 52% positive.  ▪Injecting Partners of PWID: 99.4% tested and 71% positive.  ▪MSM: 87.0% tested and 37% positive.  ▪Sexual Partners of PWID: 83.9% tested and 96% positive. | Not assessed | Not assessed | Not assessed | Not assessed |
| 12 | Oldenburg CE et al., 2018, Zambia. | To assess the effect of two health system approaches to distribute HIV self-tests on the number of female sex workers’ client and nonclient sexual partners. | Study type: Quantitative observational cohort. Population: Undiagnosed FSWs. Median age: 25 years (IQR) 21–30]. Sample size: 965 | LK | 23/1/2024 | JH, GC, JL | HIVST index testing | Secondary distribution  Partner referral slip to a health facility for HIVST | Not assessed | Not assessed | ▪Overall acceptance: 90.10% | ▪Nearly 4000 individuals and partners tested. ▪HIV-positive rates increased from 16.5% to 26.4% at 1 to 4 months using HIVST. | Not assessed | Not assessed |
| 13 | Dvora L. et al., 2022, South Africa. | To examine how HIVST can be incorporated as part of index partner testing in the rural South African context. | Study type: RCT, Trail Registered: ClinicalTrials.gov NCT02386215 Population: 180 PLHIV Women,  Age: ≥18 years, mean 35 years. | LK | 23/1/2025 | JH, GC, JL | HIVST index testing | Secondary distribution Contact referral/client | ▪80% of index clients distributed HIVST to their partners.  ▪High comfort and satisfaction. | ▪ 78% in HIVST vs. 55% in SOC tested. ▪Positivity: 14% HIVST vs. 12% SOC. ▪ART initiation: 67% HIVST vs. 100% SOC. ▪PrEP initiation: 5% HIVST vs. 16% SOC. | Not assessed | Not assessed | Not assessed | Barriers: Lower ART/PrEP initiation in the HIVST arm.  Facilitators: Counseling, distribution ease, high acceptability. |
| 14 | Zishiri V et al., 2022, South Africa. | To evaluate the programmatic implementation of partner-delivered self-testing through antenatal care (ANC) attendees and people newly diagnosed with HIV by assessing use, positivity, linkage and cost per kit distributed. | Study type: Observational cohort, mixed methods.  Population: HIV-positive ANC women.  Sample size: 4,066. | LK | 2/3/2024 | JH, GC, JL | HIVST index testing | Secondary distribution of Health facility-based distribution. | ▪ The high partner acceptance rate of HIVST kits (95.8%) demonstrates index testing acceptability for partner notification. | ▪1,649 Index clients reached 79.6% offered self-test kits to partners. ▪95.8% of partners accepted the HIVST kit. ▪95.9% of partners reported using the kit. | Not assessed | Not assessed | ▪Cost-effective scale-up in resource-limited settings. ▪Average cost per kit ranged from US$7.90 to US$14.81. ▪Human resource costs were major cost drivers. ▪Low distribution volumes in DKK led to higher average costs. | Not assessed |
| 15 | Choko AT et al., 2021, Malawi. | To investigate whether secondary administration of HIVST kits, with or without an additional financial incentive, via women receiving antenatal care (ANC) or via people newly diagnosed with HIV (i.e., index patients) could improve the proportion of male partners tested or the number of people newly diagnosed with HIV. | Study type: RCT.  Trial registered: ClinicalTrials.gov, NCT03705611. Population: HIV Undiagnosed ANC women  and diagnosed ANC women. Age: ≥18 years.  Sample size: ANC women 4544,  ANC index women 708 | LK | 25/2/2024 | JH, GC, JL | HIVST index testing | HIVST standard care group HIVST only group HIVST plus financial incentive group | Index cohort  ▪Standard care: Lost to follow-up (23.5%) ▪HIVST group: Lost to follow-up (23.5%) ▪HIVST+Financial group: Lost to follow-up (42.4%). Follow-up | Index cohort:  Standard care:  The mean reported partner testing per cluster was 35.0%. HIVST only group: The mean reported partner testing per cluster was 73.0%. HIVST Plus Financial Incentive Group: Mean reported partner testing per cluster: 65.2%. | ANC cohort  98.2% of eligible women were recruited into the study and 75.7% of them were successfully interviewed after 28 days.  Standard care: Lost to follow-up (22.6%) HIVST group: Lost to follow up (17.6%) HIVST+Finanical Group : (33.3%) | ANC cohort  Standard care group: 35.0%  HIVST-only group: 73.0% (adjusted RR 1.71, 95% CI 1.48-1.98; p<0.0001) HIVST plus financial incentive group: 65.2% (adjusted RR 1.62, 95% CI 1.45-1.81; p<0.0001) | ▪Supply-side issues limit outcomes in Malawi. ▪Strategies needed for better testing efficiency in HIV programs. ▪Challenges in optimizing HIVST accuracy, especially in rural areas. | Not assessed |
| 16 | Zewdie K et al., 2022, Kenya. | To evaluate the effectiveness of a focused partner HIVST strategy to stimulate the identification of partners of index PLHIV in public health HIV clinics for PrEP or ART programs. | Study type: Quantitative observational cohort.  Population: HIV-positive individual Age: ≥18 years Median age: 32 years, (IQR 26–40).  Sample size: 313 | LK | 6/3/2024 | JH, GC, JL | HIVST index testing | Partner referrals slip to a health facility for HIVST.  Secondary direct distribution. | ▪73% of participants discussed HIV testing with their partners: 76% in the invitation strategy vs 71% in the HIVST strategy. | ▪52% of partners in the HIVST strategy tested vs 38% in the partner referral slip.  ▪Secondary distribution, 73% in the HIVST strategy vs 50% in the invitation tested.  ▪25% (35/139) tested positive for HIV. | Not assessed | Not assessed | Not assessed | Barrier:  ▪Lack of nearby clinic, condom use, time constraints, lack of disclosure, fear of side effects. ▪14% reported social harm, including relationship breakups and abuse. |
| 17 | Luo M et al., 2016, China. | To analyze the uptake and infection status of HIV testing for sexual partners of newly diagnosed HIV-positive MSM. | Study type: Cross-sectional, quantitative  Population: HIV-positive MSM Sample size: 435 enrolled as index cases. | LK | 4/2/2024 | JH, GC, JL | HIVST index testing | CHCT partner notification PN  IAPN Oral HIV self-testing PN  Patient Referral PN | ▪17.43% of newly diagnosed MSM accepted index testing (435 out of 2495). ▪Patient referral and CHCT mode were most frequently selected by ICs (163, 37%). | ▪Overall, 87% of reachable partners accepted HIV testing, with a 16% testing yield.  ▪Sexual partners reached through the IAPN mode showed the highest testing uptake at 94%. | Not assessed | Not assessed | Not assessed | Not assessed |
| 18 | Agot K et al., 2020, Kenya. | To explore the experiences of these women when distributing HIVST kits to their sexual partners, how their partners reacted to self-testing and the test results, and how their sexual and other relationships were affected by the test results. | Study type: RCT.  Trail Registered: ClinicalTrials.gov NCT02386215.Population: Women index clients. Age: 18-39 years  Sample size: 600 | LK | 3/2/2024 | JH, GC, JL | HIVST index testing | Not assessed | ▪Partner reactions varied, with most supportive but some challenges convincing partners to test. ▪HIVST reduced uncertainty in relationships, promoted mutual support, and facilitated safer sex practices. | Not assessed | Not assessed | Not assessed | ▪Participants understood testing procedures well, using instruction sheets. ▪HIV-positive women safely distributed HIVST kits and effectively discussed testing importance. ▪Testing approaches considered convenience, confidentiality, and the significance of knowing one's status. | Not assessed |
| 19 | Mujugira A et al., 2023, Uganda. | To evaluate the benefits and risks of secondary distribution of HIVST from PLHIV. | Study type: RCT. Trail Registered: ClinicalTrials.gov, NCT03705611. Population: Pregnant women living with HIV, Age: ≥18 years, [IQR] 23–30).  Sample size: 500 | LK | 24/2/2024 | JH, GC, JL | HIVST index testing | Secondary distribution | Not assessed | HIVST Arm ▪48.5% of male partners underwent HIV testing. ▪86.9% of newly identified HIV-positive male partners-initiated ART. SOC Arm ▪44.6% of male partners tested for HIV. ▪92.3% of newly identified HIV-positive male partners started ART. | Not assessed | Not assessed | Not assessed | Not assessed |
| 20 | Ye ZH et al., 2023, China. | To assess how an intervention (aPS with CBOs and HIV self-testing, aPSST) affects HIV partner testing in newly and previously diagnosed MSM, and its impact on identifying new testers, positives, linkage to care, and safety. | Study type: RCT. Trail Registered: chictr.org.cn (ChiCTR2000038784).  Population: HIV-positive MSM  Age: 18–60 years Sample size: 325 | LK | 24/1/2024 | JH, GC, JL | HIVST index testing | Assisted partner notification Facility-based routine partner notification. | Not assessed | ▪aPSS more contactable sexual partners completed HIV testing compared to the rPS group (48.1% vs. 21.9%, p < 0.001).). | Not assessed | Not assessed | For HIV-diagnosed MSM, voluntary aPS with CBOs and HIVST effectively promoted HIV partner testing. | Not assessed |
| 21 | Chen et al., 2019, Malawi. | To determine the impact of a combination intervention versus passive partner notification on the identification of persons with HIV infection and their sexual and social contacts in Malawi. | Study type: RCT. Trail Registered: ClinicalTrials.gov, number (NCT02467439). Population: Index clients, aged 18 years.  Sample size: 3594 | LK | 11/3/2024 | JH, GC, JL | Regular RDTs for index testing. | Passive Partner  Contract Partner  Combination of passive and contact | Not assessed | Intervention vs. pPN.  ▪Intervention saw 29% contact presence vs. 15% in pPN. | Not assessed | Not assessed | Not assessed | Not assessed |
| 22 | Madsen T et al., 2019, West Africa. | To investigate the acceptance, preferred choice of method, and outcome of Partner Notification (PN) among HIV-infected patients in Guinea-Bissau, West Africa. Additionally, the study aimed to identify challenges in the implementation of PN in this setting. | Study type: Quasi-experimental Trail registration not specified Population: HIV-positive individuals (63% women and 47% men), Median age was 38 years (IQR) 32–45).  Sample size:697 | LK | 2/1/2024 | JH, GC, JL | Regular RDTs for index testing. | Passive referral Contract referral Provider referral: 39 partners | ▪Of the 697 HIV patients, 495 patients (71.0%) accepted PN for at least one partner, while 202 patients (29.0%) declined PN. | ▪Of the identified partners, 41.9% tested positive for HIV. ▪Married partners had a higher HIV prevalence compared to non-marital partners. ▪Most patients chose passive referral for PN. ▪A small percentage of patients opted for a contract/provider referral. | Not assessed | Not assessed | ▪Newly diagnosed patients had higher partner referral success.  ▪Married patients are more successful in partner referral. | Barrier: ▪Geographical distances (51.5%),. ▪Fear of abandonment (22.8%). ▪Not being able to reach partner (14.4%).  ▪Partner refusing testing (2.5%). ▪Fear of stigma (1.0%) and other reasons (n = 16, 7.9%). |
| 23 | Cherutich P et al., 2017, Kenya. | To establish whether or not assisted partner services increase HIV testing, diagnoses, and linkage to care among sex partners of people with HIV infections in Kenya. | Study type: RCT  Trail Registered: ClinicalTrials.gov, number NCT01616420. Population: HIV index clients, median ages 30, (IQR 25-38), Sample size: Total 1305 | LK | 11/1/2024 | JH, GC, JL | Regular RDTs for index testing. | Contact/Immediate referral Passive referral | ▪Initial index clients approached 1760  ▪841 under immediate aPNS (acceptance rate 69%). ▪919 under delayed aPNS (acceptance rate 66%). | ▪Immediate group: 392 (67%) tested, 136 (23%) new HIV+, 88 (15%) in care. ▪Delayed group: 85 (13%) tested, 28 (4%) new HIV+, 19 (3%) in care. ▪Immediate intervention increased testing five-fold. | Not assessed | Not assessed | Not assessed | Barrier: One violent event per group related to notification/study. Facilitators: No rise in intimate partner violence with assisted services; |
| 24 | Goyette MS et al., 2018, Kenya. | To investigate whether the history of intimate partner violence (IPV) modified APS effectiveness and risk of relationship dissolution. | Study type: RCT. Trail Registered: ClinicalTrials.gov, number NCT01616420. Population: Index patients. Age: No age categories were mentioned.  Sample size: 119 | LK | 13/1/2024 | JH, GC, JL | Regular RDTs for index testing. | Not assessed | Not assessed | Not assessed | Not assessed | Not assessed | Not assessed | Barrier: ▪IPV history linked to younger age and singleness in females, and condom use in males. ▪IPV history increased the risk of relationship dissolution. |
| 25 | Masyuko SJ et al., 2019, Kenya. | To assess the efficacy of assisted partner services (aPS) based on the characteristics of index participants, including region of residence, rural/peri-urban versus urban location, gender, age, and knowledge of HIV status. | Study type: RCT. Trail Registered: ClinicalTrials.gov, number NCT01616420. Population: Newly diagnosed index clients, Age: ≥18-year-old, median age was 30 years (IQR 25, 38). Sample size: 1119 | LK | 10/2/2024 | JH, GC, JL | Regular RDTs for index testing. | Contract referral Provider referral | Not assessed | Not assessed | Not assessed | Not assessed | ▪Immediate aPS more effective than delayed aPS, especially in high-prevalence regions like Nyanza (IRR 7.2).  ▪aPN feasible for new cases for high detection.  ▪Providing aPS to females resulted in significantly higher HIV case-finding rates compared to males (IRR 9.1 vs. 3.2). | Not assessed |
| 26 | Culbert GJ et al., 2023, Indonesia. | To assess how the Impart APN model affects partner notification and HIV testing outcomes for incarcerated men with HIV. It also evaluates the feasibility and effectiveness of implementing APN programs in prisons and adds to the existing knowledge on APN interventions. | Study type: RCT.  Trail registration: ClinicalTrials.gov NCT04155320. Population: Index clients Mean aged: ≥18 years,  Sample size: 55 index clients | LK | 26/1/2024 | JH, GC, JL | Regular RDTs for index testing. | Passive referral  Contact referral | Not assessed | ▪Self-test: 18% of partners notified; 0% of partners tested.  Impart APN: 53% notified;  63% tested. | Not assessed | Not assessed | Not assessed | Not assessed |
| 27 | Uma TH et al., 2023, Ethiopia. | To assess the proportion and the factors associated with partner and family-based index case HIV testing in Woliso Town. | Study type: Quantitative cross-sectional.  Population: HIV-positive. Median age: 38 years (IQR=35-43)  Sample size: 346 (Male 126 and female 219) | LK | 2/1/2024 | JH, GC, JL | Regular RDTs for index testing. | Not assessed | ▪Acceptability Indicators: 96.5% of families tested; 93.9% disclosed HIV status. | ▪96.5% of index cases had their families tested for HIV. | Not assessed | Not assessed | Not assessed | Barrier: ▪25.5% cite stigma fear. Facilitators: Counseling aids in testing uptake. |
| 28 | Tih PM et al., 2019, Cameroon. | To describe the large-scale implementation of aPNS and overall programmatic achievements in a resource-limited setting. | Study type: Quantitative cross-sectional. Population: HIV-positive individuals. Sample size: 1,261 | LK | 7/1/2024 | JH, GC, JL | Regular RDTs for index testing. | Provider referral Contract referral Patient referral |  | ▪Of 21,057 contact persons 71% of notified contact persons.  ▪4,764 out of 21,057 contacts tested positive for HIV and 66% liked treatment. | Not assessed | Not assessed | ▪aPNS were feasible in SSA with little social harm, despite limited funding. | Barrier: ▪19.7% of index persons reported pre-existing social harms. ▪Among those with prior IPV, 43.5% experienced harm within three months before aPNS enrollment. ▪6.3% experienced adverse outcomes post-notification, mainly partnership dissolution. |
| 29 | Chelogoi E et al., 2020, Kenya. | To investigate factors that obstruct assisted partner notification services in this setting. | Study type: Quantitative cross-sectional Population: HIV-positive individuals.  Age: ≥18 years Sample size: 423 (Male 190, and female 228). | LK | 12/1/2024 | JH, GC, JL | Regular RDTs for index testing. | Not assessed | Not assessed | Not assessed | Not assessed | Not assessed | Not assessed | Barrier: ▪Embarrassment: 55% more likely not to participate in aPNS. ▪Stigma: 44% more likely not to participate. ▪Unfriendly services: Nearly 4 times higher risk of non-participation. |
| 30 | Shamu S rt al., 2019, South Africa. | To compare index client tracing modality’s outcomes with other CBCT recruitment modalities (mobile, workplace, home-based), 2015–2017 | Study type: Quantitative cross-sectional.  Population: All populations including HIV-positive individuals Age: ≥12 months.  Sample size: 1,282,369 | LK | 19/1/2024 | JH, GC, JL | Regular RDTs for index testing. | Contact referral  passive referral  Anonymized Referral | Not assessed | ▪HIV testing Year 1: 660,351, and Year 2: 622,018 tests. ▪HIV Positivity: Overall: 7.4% (6.6% in year 1, 8.3% in year 2). ▪Linkage to Care: Overall: 56.1%.Year 1: 33.3%, Year 2: 78.9%. | Not assessed | Not assessed | Not assessed | Not assessed |
| 31 | Andriyanto A et al., 2023, Indonesia. | To analyze the determinants of notification of spouses of people with HIV-AIDS (PLWHA) in Care, Support, and Treatment Services in Mojokerto City. | Study type: Quantitative cross-sectional  Population: PLWHA, age 26-35 years. Sample size: 92 | LK | 21/1/2024 | JH, GC, JL | Not assessed | Double referral  Patient referrals | ▪12% of respondents rated the notifications as very good. ▪2.2% of respondents rated the notifications as good. ▪20.7% felt the notifications were enough. ▪The majority, 41.3% of respondents, rated the notifications as bad. | Not assessed | Not assessed | Not assessed | Not assessed | Not assessed |
| 32 | Culbert GJ et al., 2020, Indonesia. | To examine the willingness of people living with HIV (PLHIV) in prison to participate in assisted HIV partner notification services and to explore their reasons for or against disclosing their HIV-positive status to their partners. | Study type: Quantitative cross-sectional. Population: All populations including HIV-positive individuals. Age: ≥12 months.  Sample size: 1,282,369 | LK | 6/2/2024 | JH, GC, JL | Not assessed | Provider referral | 66.4% endorsed provider referral for notifying sex partners. 72.4% endorsed provider referral for notifying drug-injecting partners. | Not assessed | Not assessed | Not assessed | Not assessed | Barriers: Stigma and privacy concerns deterred disclosure. Protecting partners and moral duty drove disclosure. |
| 33 | Gitig GG et al., 2021, Tanzania. | To determine predictors of partner's elicitation among index HIV-positive clients. | Study type: Quantitative cross-sectional  Population: HIV-positive clients who were diagnosed within the previous 12 months, Mean age: 37 years old. Sample size: 427. | LK | 27/1/2024 | JH, GC, JL | Regular RDTs for index testing. | Contact referral APNS | Not assessed | ▪Proportion disclosing partners' information: 55.5% ▪Preferred notification approach: 71% opted for passive notification. | Not assessed | Not assessed | Not assessed | ▪Barrier: Inability to locate partners' residential places (29%). ▪Facilitator: Availability of privacy during HIV care. |
| 34 | Cibangu K, 2022, Zambia. | To review existing medical files and registers in the Matero subdistrict of Zambia to describe existing information on index testing and propose better ways to improve HIV index testing positivity yield. | Study type: Quantitative cross-sectional.  Population: Index Clients  Age range: 16 to 78 years, mean age of 34 years  Sample size: 604 index clients | LK | 30/1/2024 | JH, GC, JL | Regular RDTs for index testing. | Assisted partner notification  Routine regular partner notification. | ▪Acceptance Rate: 92.6% of the clients  ▪Offer Index testing=617, 604 accepted (98%). | ▪Of the 845 contacts elicited, 673 reached/tested (80%).  ▪53.5% tested HIV negative, 13.4% tested positive, and 12.8% were known positives. ▪79.6% of positive cases started antiretroviral therapy within 7 days. | Not assessed | Not assessed | Not assessed | Not assessed |
| 35 | Mwango LK et al., 2020, Zambia. | To present the CIRCUITS approach to case finding and examining HIV positivity yield and antiretroviral therapy (ART) linkage across index testing and targeted community testing modalities, with sub-analyses by sex, age groups and district. | Study type: Quantitative cross-sectional. Population: Male Index clients Sample size: 391. | LK | 9/2/2024 | JH, GC, JL | Regular RDTs for index testing. | Assisted Partner Notification  Community-based testing | Index testing  ▪Out of 12,391 index clients, 11,480 accepted index testing,(92.6% acceptance rate).  ▪Acceptance rate for females: 88.4% ▪Acceptance rate for males: 89.2% | Index testing  ▪Elicitation ratio for index testing: 1:1.8. ▪Contact tracing rate: 77.8%. ▪Positivity yield among traced contacts: 44.7%. ▪Overall ART linkage rate for index testing: 88.7%. | Not assessed | Not assessed | Not assessed | Not assessed |
| 36 | Afe AJ et al., 2021, Nigeria. | To assess the outcome of implementing Partner HIV Testing Services (PNS) in three public secondary health facilities in the north-central region of Nigeria. | Study type: Quantitative cross-sectional.  Population: HIV-positive client  Sample size: 705 | LK | 18/2/2024 | JH, GC, JL | Regular RDTs for index testing. | Assisted partner notification | Uptake of PNS: 100% among index cases, indicating high acceptability. HIV testing yield of 27% higher than routine walk-in clients, suggesting the effectiveness and acceptability of PNS. | Uptake of PNS: 100% among index cases Sexual partners contacted: 98% underwent HIV testing services Index cases who brought their sexual partners for HIV testing: 100% HIV Positivity Yield: 27% and 21.6%. ART linkage. | Not assessed | Not assessed | Not assessed | Not assessed |
| 37 | Fu X et al., 2016, China. | To explore the feasibility and efficiency of sexual PN and HIV testing among HIV-positive MSM in cooperation with MSM-serving CBOs, we performed a pilot study in two Chinese cities. | Study type: Quantitative cross-sectional.  Population: HIV-positive MSM. Median age: 33 years (IQR 14.46. Sample size: 253 | LK | 22/2/2024 | JH, GC, JL | Regular RDTs for index testing. | Assisted partner notification | Not assessed | ▪Uptake of Index Testing to Partner Notification Services: ▪Over 96% of previously identified HIV cases and 67.7% of screened reactive. ▪Among reached sexual partners, 10.5% screened reactive, with 82.8% confirmed HIV-positive. | Not assessed | ▪71.6% of reached sexual partners received HIV testing. | ▪Conducting partner notification and testing mobilization with MSM-serving community-based organizations (CBOs) was feasible and efficient. | ▪Barriers: Fear of disclosing HIV status and lack of contact information for partners. |
| 38 | Ugbena ER et al., 2013, China. | To describe the level of acceptability and outcome of PNS among HIV-positive KPs using the various approaches for PNS services and to describe the proportion of HIV-positive KPs who accepted partner notification services after diagnosis | Study type: Cross-sectional reported. Population: HIV-positive PWIDF, PWIDM, MSM, and FSW. Sample size: 47668 KPs recruited and 846 index included for analysis. | LK | 23/2/2024 | JH, GC, JL | Regular RDTs for index testing. | Passive method  Contract method  Provider-initiated Dual method | ▪Overall acceptance rate of index testing among KPs: 34% | ▪Number of Partners elicited by HIV-positive KPs 941 Number of partners notified and tested for HIV=938 (99.60%)-uptake  ▪Positivity rate among tested partners: (421, 45%). | Not assessed | Not assessed | Not assessed | Not assessed |
| 39 | Emeh A et al., 2021, Nigeria. | To determine the positivity yield and identify factors influencing the yield from index testing strategy in selected healthcare facilities in Ondo State, southwest Nigeria. | Study type: Quantitative cross-sectional. Population: Index clients, Mean age: 38.52 years.  Sample size: 904 | LK | 27/2/2024 | JH, GC, JL | Regular RDTs for index testing. | Passive/Client Referral Provider Referral Dual Referral Contract Referral | ▪82% of partners contacted by phone, showing high acceptability. ▪56% effectiveness for assisted referral, indicating positive attitudes among HIV-positive individuals. | ▪904 index clients and partners participated; a high female proportion (60.6%) suggests active testing service engagement. ▪HIV positivity rate among partners: 20%, 100 linked to ART. | Not assessed | Not assessed | Not assessed | Not assessed |
| 40 | Buhikire K et al., 2018, Uganda. | To find out the predictors of successful contact tracings and testing of partners of HIV+ individuals and possible barriers to contact. | Study type: Quantitative cross-sectional Population: HIV+ men and women,  Age: ≥15 years old.  Sample size: 464 | LK | 29/2/2024 | JH, GC, JL | Regular RDTs for index testing. | Passive referral  Contact referral  Method unknown | 464 HIV+ index clients identified 660 sexual partners. | ▪Named sexual partners: 660 ▪Partners aware of index client's HIV status: 57% ▪Partners contacted by the program: 51% ▪Contacted partners tested for HIV: 58% (193 out of 334). | Not assessed | Not assessed | ▪Less disclosure at healthcare facilities than hospitals. ▪Current status strongly influences contact. | Barrier:  Challenges include partners' prior awareness of HIV status and logistical issues with partner contact. Success is linked to disclosure method and partner type. |
| 41 | Joel JN et al., 2017, Kenya. | To describe the implementation strategies of the index testing program in Nairobi County, Kenya, and assess outcomes along the HIV index testing cascade over the first two years of implementation. | Study type: Quantitative cross-sectional  Population: Index HIV cases  Age: ≥15 years  Population: General population. Sampler size: Year 1: 4906, Year 2: 6633 | LK | 1/3/2024 | JH, GC, JL | Regular RDTs for index testing. | Passive referral Provider referral | ▪HIV detection rates over two years: 25.2%, 24.1% ▪Higher detection in women and those aged 50+: 30.0%, 32.4% ▪Index testing effective for general and key populations ▪Increased HIV-positive case identification over time. | ▪Testing of partners increased from 42.4% to 74.9% in the general population. ▪Testing of partners in key populations decreased from 52.4% to 40.7%. ▪However, HIV detection among key populations rose from 8.6% to 23.9%. | Not assessed | Not assessed | Not assessed | Not assessed |
| 42 | Mahachi N et al., 2019, Zimbabwe. | To describe the implementation of index testing and PNS under the Zimbabwe HIV Care and Treatment (ZHCT) project and the resulting uptake, HIV positivity rate and links to HIV treatment. | Study type: Cross-sectional Population: Index clients  Sample size: 25,704 index clients, of whom 24,453 (95.1%) consented to participate. | LK | 5/3/2024 | JH, GC, JL | Regular RDTs for index testing. | APNS | ▪25,704 index clients participated. 24,453 (95.1%) consented to index testing and PNS. ▪ Monthly positivity rate from index testing: 32.6% ▪Significantly higher than other facility testing modalities: 4.1% (p < 0.001) | ▪55,149 contacts who received HIV testing services. Of these 55,149 contacts, 15,944 (29%) tested HIV positive | Not assessed | Not assessed | Not assessed | Not assessed |
| 43 | Kariuki RM et al., 2020, Kenya. | To support policymakers and health managers in improving the implementation of PNS services aimed at achieving increasing HIV case identification and coverage of care and treatment. | Study type: Quantitative cross-sectional Population: Male and female Index case Sample size: 183 | LK | 11/3/2024 | JH, GC, JL | Regular RDTs for index testing. | Provider referral (51%) Contract referral (45%).  Dual referral (4%) | ▪Overall, 162 [89%] index clients accepted PNS. ▪Acceptance rate was higher among male index clients [92%] than in females [86%]. | ▪Only 15% (34) of sexual partners’ HIV status is known to index clients. ▪HIV status is unknown for 85% (182) of sexual partners. | Not assessed | Not assessed | Not assessed | Not assessed |
| 44 | Remera E et al., 2022, Rawanda. | To assess the relative effectiveness of VAPN modalities in identifying undiagnosed HIV infections | Study type: Quantitative observational cohort. Population: New HIV diagnosis and PLHIV on ART. Sample size: 6336 | LK | 3/1/2024 | JH, GC, JL | Regular RDTs for index testing. | Contract referral: 2,605 partners notified:  Provider referral: 3,394 partners notified.  Dual referral: 1,691 partners notified | ▪Percentage of respondents who accepted HIV testing after being invited: 92.9% (7,690 out of 8,276). | ▪Total index cases reporting partners with unknown HIV status: 6,336. ▪Percentage of invited partners tested: 89.7% (10,432 out of 11,633). ▪Percentage accepting testing: 92.9% (7,690 out of 8,276). ▪HIV-positive 7.1% (546 out of 7,690). | Not assessed | Not assessed | Not assessed | Not assessed |
| 45 | Golden MR et al., 2023, Mozambique. | To assess the safety and effectiveness of assisted partner notification services during scale-up in Mozambique. | Study type: Observational cohort, quantitative.  Population: Newly testing HIV-positive, previously diagnosed HIV infection received APS  Sample size: 9219 | LK | 9/1/2024 | JH, GC, JL | Regular RDTs for index testing. | Assisted Partner Notification Services | Not assessed | ▪Total index cases receiving APS: 9219 ▪Percentage of cases identifying at least one sex partner: 93% ▪Untested partners without prior HIV diagnosis tested for HIV: 69% | Not assessed | Not assessed | ▪More women than men received APS. ▪Antenatal clinic attendees had a higher APS rate than other sectors. ▪Younger and older patients were less likely to receive APS than those in middle age. | ▪ Fear of adverse events excluded. ▪ Fear of loss of support, and untested partners linked to adverse outcomes. |
| 46 | Opeyemi A et al., 2013, Nigeria. | To evaluate HIV partner notification services at the University of Abuja Teaching Hospital, comparing their effectiveness in identifying positive cases to other testing methods at the facility. | Study type: Mixed method observational cohort. Population: HIV-positive individuals Age: ≥15 years.  Sample: 451 | LK | 14/1/2024 | JH, GC, JL | Regular RDTs for index testing. | Facility-based routine partner testing | ▪Total clients with HIV infection interviewed: 451 ▪Acceptance rate for Partner Notification Service: 86.2% ▪Total partners elicited: 457 ▪Average number of partners per client: 1.2 | ▪84.9% of partners were notified of their risk of exposure to HIV. ▪ 30% previously knew their HIV-positive status, ▪19.2% were newly diagnosed, and 50.8% tested non-reactive. | Not assessed | Not assessed | Not assessed | Not assessed |
| 47 | Katbi M et al., 2018, Nigeria. | To design and evaluate the impact of interventions aimed at identifying previously undiagnosed cases of HIV infections among the sexual partners of index Persons Living with HIV (PLHIV). | Study type: Quantitative quasi-experimental. Trial registration not specified, mixed-method.  Population: newly diagnosed PLHIV and those already on ART. Sample size: 1277 | LK | 22/1/2024 | JH, GC, JL | Regular RDTs for index testing. | Not assessed | ▪Total index cases counseled and interviewed: 1277 ▪Index clients agreeing to disclosure: 879 Disclosure rate: 68.3% | ▪Sexual partners identified: 888 ▪Sexual contacts traced: 97.9% (870) ▪Sexual contacts tested for HIV: 85.2% (741) ▪% tested positive: 51% | Not assessed | Not assessed | Not assessed | Not assessed |
| 48 | Sharma M et al., 2021, Kenya | To assess aPS acceptability, reasons and predictors of non-enrollment among females in an ongoing implementation project of aPS scale-up in western Kenya, a region with high HIV prevalence (15%) | Study type: Quantitative observational cohort.  Population: HIV-positive females  Age: ≥15 years old.  Sample size: 839 | LK | 28/1/2024 | JH, GC, JL | Regular RDTs for index testing. | Provider Notification Contract Referral Dual Referral Voluntary Consent | ▪Overall, 80% of HIV-positive female index clients enrolled in assisted partner services (aPS). | ▪Overall, 77% of male partners named by female index clients were traced and tested for HIV by aPS providers. ▪Tested partners: 44% HIV+ ▪Newly diagnosed partners: 17% ▪Partners on ART: 97% | Not assessed | Not assessed | Not assessed | Not assessed |
| 49 | Kiene SM et al., 2017, Uganda | To assess the perceived feasibility and acceptability of index partner HIVST by HIV-positive clients versus partner referral slips (standard of care) among HIV-positive clients in Uganda. | Study type: Quantitative observational cohort.  Population: HIV-positive men and women, Sample size: 304 (152 males and 152 females). | LK | 5/2/2024 | JH, GC, JL | Regular RDTs for index testing. | Provider-Initiated Testing and Counseling | High disclosure and engagement rates indicate good acceptability. | High disclosure rates (men: 97.4%, women: 96.0%) and significant efforts to engage partners in testing (over 80% asked partners for testing). | Not assessed | Not assessed | Not assessed | Barrier: ▪Lower testing rates reported by women due to gender-specific challenges. Facilitators ▪Previous testing and positive social support. |
| 50 | Semple SJ et al., 2018, Mexico | To assess the uptake and outcomes of a partner notification model, primarily provider referral, among MSM and TW in Tijuana, Mexico, while identifying factors influencing the notification of sexual partners. | Study type: Mixed method observational cohort.  Population: HIV-positive MSM and TW. Median age: 28 years (IQR: 23–35). Sample size: 36 | LK | 8/3/2024 | JH, GC, JL | Regular RDTs for index testing. | Provider referral  Contact referral | Among notified sexual partners, 76% agreed to be screened for eligibility, with 51% of them eligible for enrollment and HIV testing. | ▪94% of HIV+ index patients opted for provider referral. ▪Resulted in 70% partner notification. ▪Partner-based index patients saw 59% partner notification with provider referral. | Not assessed | Not assessed | Not assessed | Barrier:  ▪Lack of reliable contact information and suspicion about notification staff intentions. |
| 51 | Offorjebe OA et al., 2020, Malawi | To assess the perceived feasibility and acceptability of index partner HIVST by HIV-positive clients versus partner referral slips (standard of care) among HIV-positive clients in Malawi. | Study type: Qualitative cross-sectional study. Population: HIV-positive.  Sample size: 404 (male 159, female 245) | LK | 1/1/2024 | JH, GC, JL | HIVST index testing & RDT | Facility-Based Partner Referral Slips for HIVST  Home-Based HIVST.  HIV-positive clients are more comfortable with HIVST kits than referral slips (90% vs. 81%). | Quantitative result  90% of clients reported being comfortable delivering HIVST kits. 81% of clients reported being comfortable delivering partner referral slips. Clients preferred HIV self-testing (HIVST) over clinic-based testing for their partners (77% vs. 66%). | 404 HIV-positive clients participated in the index partner testing survey.  ▪Only 13% of both men and women were newly diagnosed with HIV within the prior 3 months. ▪87% of men and 92% of women were currently on ART | Not assessed | Not assessed | ▪More clients believed their partners would test using HIVST compared to returning for clinic-based HIV testing following receipt of partner referral slips (77% vs. 66%). | Barrier: ▪Worries about partner trust, intimate partner violence concerns, and gender norms hinder HIV self-testing kit distribution.  Facilitators  ▪HIV self-testing is widely accepted among couples, strengthening relationships, and providing valued privacy. ▪ Married index clients willing to use HIVST. |
| 52 | Edosa M et al., 2022, Ethiopia | To assess the magnitude and factors associated with ICHT among HIV patients attending ART in Nekemte town public health facilities. | Study type: Mixed cross-sectional study.  Population: All HIV-positive individuals. Age: ≥18 years. Sample size: 396 | LK | 2/1/2024 | JH, GC, JL | Regular RDTs for index testing. | APNS | ▪85.2% (95% CI=84.9–91.1) of participants agreed to tests for partners and children. ▪Among those accepting ICHT, 88% preferred facility-based testing. | Not assessed | Not assessed | Not assessed | Not assessed | Barrier:  ▪Fear of stigma and discrimination (38%) and fear of physical violence (20%) were cited as barriers to accepting index case HIV testing. |
| 53 | Grande M et al., 2021, Botswana | To evaluate Botswana's APS for reach, effectiveness, APS value for known cases, and re-linking identified partners to care | Study type: Quantitative observational cohort,  Population: HIV-positive individuals  sample size: | LK | 12/2/2024 | JH, GC, JL | Regular RDTs for index testing. | Not assessed | ▪Phase 1: 87% acceptance. ▪Phase 2: 95% acceptance. | ▪Phase 1: 4,401 partners named. ▪Phase 2: 1,670 partners named. ▪Phase 1: 66% tested, 22% HIV+. ▪Phase 2: 54% tested, 31% HIV+. | Not assessed | Not assessed | Not assessed | Not assessed |
| 54 | Selvaraj K et al., 2017, India | To assess the status of partner testing and factors influencing it among PLHIV registered in selected ART centres in Gujarat, India, from 2011–2015. | Study Type: Mixed method cross-sectional. Population: diagnosed PLHIV. Mean age: 38 years. Sample size: 3884 (60% were male and 40% female). | LK | 6/1/2024 | JH, GC, JL | Regular RDTs for index testing. | Not assessed | Not assessed | Not assessed | Not assessed | Not assessed | Non-testing linked to Male, >26 years, illiterate, professional, alcohol/tobacco use, key populations (MSM, FSW, IDU), WHO stage 3/4, CD4 ≤ 350 cells/mm³Not assessed | Barrier: ▪Stigma and discrimination. ▪Sex and age dynamics.  ▪Social and legal factors. Facilitator: ▪Increased access to HIV testing through decentralization. ▪Direct access to HIV counselling and testing without queuing. ▪Availability of free services. ▪Counseling services offered by multiple stakeholders. ▪Travel reimbursement and positive patient attitudes. |
| 55 | Rahmalia A et al., 2022, Indonesia | To investigate HIV status disclosure and partner testing practices among WLWH in urban Bandung, Indonesia, to identify needs in partner notification services. | Study type: Exploratory Mixed method Population: Women living with HIV. Age: 18 to 42 years old, median age 35 years old. Sample size: 122 | LK | 26/1/2024 | JH, GC, JL | Regular RDTs for index testing. | aPNS | Not assessed | Among 122 female patients receiving HIV care, 92.8% disclosed their status, but only 53.7% of new partners were tested. | Not assessed | Not assessed | Not assessed | Barrier: ▪Fear of rejection hindered initial disclosure, delaying testing. ▪Partnership counselling and tailored guidelines are recommended to address barriers.  Facilitators ▪HIV diagnosis prompted women with PLHIV to form new partnerships. ▪Some women PLHIV disclosed their status to new partners independently. |
| 56 | Wamuti B et al., 2023, Kenya | To explore the factors affecting implementation fidelity to aPS in two high-HIV prevalence counties in western Kenya | Study type: Exploratory mixed method. Population: Newly diagnosed HIV-positive female. Age: 35 years, age range 25 to 52 years. Sample size: 3017 | LK | 7/2/2024 | JH, GC, JL | Regular RDTs for index testing. | APNS | Of the 3017 Partners notified, 98% were successfully traced. Out of the traces, 95% were successfully located and tested. | Not assessed | Not assessed | Not assessed | ▪Positive provider attitudes and conducive work environments promoted aPS.  ▪Negative responses from Most Significant Partners (MSPs) and challenging tracing conditions hindered aPS implementation fidelity. | Not mentioned |
| 57 | Vermandere H et al., 2021, Mexico | To explore the awareness of and need for HIV partner notification, as well as to outline potential strategies for APNS based on identified barriers and facilitators. | Study type: Exploratory qualitative study.  Population: HIV-positive MSM. Mean age: MSM 30 years old, TG mean age 43 years old.  Sample size: MSM=12, and TG=11 | LK | 22/1/2024 | JH, GC, JL | Not assessed | APNS | •Participant's suggestions for improving assisted partner notification services (APNS). •Participants' acknowledgement of the benefits and importance of APNS. | Not assessed | Not assessed | Not assessed | • Strategies outlined for promoting the feasibility of implementing APNS. | Barrier: ▪Fear of stigma, rejection, violence, and logistical issues like lacking contact information for casual partners. Facilitator:  ▪Relationship type (formal vs. casual), knowledge about HIV, and the use of social media and dating apps. |
| 58 | Yan XM et al., 2022, China | To examine facilitators and barriers of HIV PS using qualitative analysis. | Study type: Exploratory qualitative study. Population: HIV-positive MSM. Age: 18 to 25 years old.  Sample size: 53 | LK | 26/1/2024 | JH, GC, JL | Not assessed | APNS | Not assessed | Not assessed | Not assessed | Not assessed | Not assessed | ▪Facilitator: Include individual motivations, community support, and societal policies.  ▪Barrie: Encompass psychological stressors, negative partner reactions, and societal stigma. |
| 59 | Quinn et al., 2018, Uganda | To understand community reactions to both passive and assisted partner notification approaches, with a specific focus on comparing responses between communities with differing HIV risk. | Study type: Exploratory qualitative study. Population: Healthcare Providers, Community members, fishing Communities, and mainland communities.  Median age: 28 years fishing communities, Median age: 26 years Mainland, Median age: 32 years health care providers  Sample size: 63 IDIs and 6 FGDs | LK | 1/2/2024 | JH, GC, JL | Not assessed | Provider Referral  Passive Referral  Contract Referral | ▪Provider-assisted notification is favoured for revealing HIV status and reducing transmission. ▪Providers worry about tracing issues and confidentiality breaches. ▪Participants fear blame and social harm after notification. | ▪Increased number of partners coming in for testing compared to self-disclosure or passive referral. ▪67% of partners notified with immediate provider assistance sought HIV testing. | Not assessed | Not assessed | ▪Provider-assisted notification leads to higher yields of partners newly diagnosed with HIV compared to passive referral. | Barrier:  ▪Loss of trust feared in serodiscordant couples.  ▪Suicide is seen as a risk in partner notification. ▪Concerns over break-ups, violence, and family issues.  Facilitator:  ▪Financial needs may keep partners together. ▪Financial dependence heightens fear of abandonment. |
| 60 | Sanga E et al., 2023, Tanzania | To explore decision-making around disclosure to sexual partners among PLHIV on ART in North-Western, Tanzania | Study type: Exploratory qualitative study.  Population: People living with HIV aged 18 years and above.  Sample size: 86 | LK | 2/2/2024 | JH, GC, JL | Not assessed | Not assessed | PLHIV commonly sought relief from "living a lie" by disclosing to partners, finding it liberating. | Not assessed | Not assessed | Not assessed | Not assessed | Barrier:  ▪Risks of disclosure included fear of stigma, rejection, and discussions about fidelity. ▪Non-disclosure was linked to avoidable deaths from untreated illness in partners. |
| 61 | Liu W et al., 2022, | To explore their experience of providing APS, the barriers and the facilitators, and how contextual factors influenced their experience. | Study type: Exploratory qualitative study. Population: Key service providers.  Sample size: 14 | LK | 14/2/2024 | JH, GC, JL | Regular RDTs for index testing. | ▪Provider referral preferred for efficiency and confidentiality. ▪Kind-hearted benefits of APS motivate providers despite challenges. | Not assessed | Not assessed | Not assessed | Not assessed | Not assessed | Barrier ▪Partner elicitation is sensitive due to fear of judgment and breaches.  ▪Partner tracing is challenging due to misinformation, geography, and suspicion/refusal. Facilitator ▪Confidentiality is crucial to protect clients' identities. |
| 62 | Aliza MW et al., 2019, Kenya | To describe barriers encountered and potential opportunities to providing aPNS to established patients living with HIV | Study type: Exploratory qualitative study.  Population: Index clients Sample size: 47 | LK | 9/1/2024 | JH, GC, JL | Regular RDTs for index testing. | APNS | Not assessed | Not assessed | Not assessed | Not assessed | Not assessed | Barrier ▪Fear of relationship repercussions and stigma. ▪Fear of blame and loss of financial support. ▪Fear of violence upon disclosure. Facilitators ▪ HCW assistance and sensitization helped with disclosure. |
| 63 | Zhang K et al., 2019, China | To describe the PN experiences of PLWH and explore the perceived facilitators of and barriers to PN using a qualitative method | Study type: Exploratory qualitative study.  Population: Diagnosed PLHIV Age: 18 to 62 years, mean age of 39.3 years. Sample size: 42 | LK | 25/2/2024 | JH, GC, JL | Regular RDTs for index testing. | APNS | Not assessed | Not assessed | Not assessed | Not assessed | Participants reported several benefits of PN, including convincing the sexual partner to have HIV testing. | Barrier: ▪Stigma and fear of negative reaction hindered disclosure. Facilitator: ▪Disclosure preserved trust and protected partners' health. ▪Support from healthcare providers and friends facilitated disclosure. ▪aPN encourages HIV testing. |
| 64 | Setia GB et al., 2021, Indonesia | To understand provider and patient perspectives on aPN from three demonstration sites in cities with a high HIV burden. | Study type: Exploratory qualitative study.  Population: Health care providers, people living with HIV and general populations, age ≥18 years.  Sample size: 40 | LK | 26/2/2024 | JH, GC, JL | Regular RDTs for index testing. | APNS | Not assessed | Not assessed | Not assessed | Not assessed | ▪Confidentiality and stigma influenced preferences. ▪Provider referral is seen as less effective by some. ▪Providers lacked confidence.  ▪Digital approaches were recognised but raised concerns. ▪Dual referral supported by all stakeholders. | Barrier:  ▪Concerned breaching confidentiality.  Facilitator:  ▪Providers felt that initial phone or short message contact was ideal for aPN. |
| 65 | Goyette M et al., 2016, Kenya | To qualitatively explore the client, community, and health system barriers to the implementation of APS in Kenya within the cluster randomized trial. | Study type: Exploratory qualitative study.  Population: PWLHIV,  Age: Median age 40, age range 30 to 47 year  Sample size: 20 | LK | 7/3/2024 | JH, GC, JL | Regular RDTs for index testing. | APNS | Not assisted | Not assessed | Not assessed | Not assessed | ▪Some opt for methods like couples testing. ▪Building trust fosters openness. ▪Stable relationships aid engagement. | Barrier: ▪Fear of Stigma, and concerns about discrimination  ▪Fear of conflict in relationships  ▪Lack of community awareness about APS  Facilitator:  ▪Clients' states of mind, trust in counsellors, and relationship dynamics influence uptake. |
| 66 | Benemariya N et al., 2023, Rawanda | To assess factors associated with partner notification among people living with HIV in Bushenge Hospital, 2018-2019. | Study type: Case-control.  Population: People living with HIV,  Age: ≥44 years old.  Sample size: 282 | LK | 20/2/2024 | JH, GC, JL | Regular RDTs for index testing. | Provider Referral Passive/Client Referral Dual Referral | Not assessed | ▪The partner notification service rate was reported at 20.8% in 2019, indicating low uptake in the Bushenge Hospital catchment area. | Not assessed | Not assessed | ▪The effectiveness of the dual referral approach suggests it is operationally feasible. | Barrier: ▪ Social, psychological, and operational challenges.  ▪The dual referral approach is highlighted as a facilitator for increasing partner notification rates. |
| 67 | Sharma M et al., 2018, Kenya | The study aimed to evaluate the cost-effectiveness and health impact of implementing Assisted Partner Services (aPS) for HIV testing and linkage to care in western Kenya. | Study type RCT. Clinical trial registration not reported.  Population: HIV-positive individuals. Age: ≥18 years.  Sample size: 1,119 | LK | 8/1/2024 | JH, GC, JL | Not used | Not assessed | Not assessed | Not assessed | Not assessed | Not assessed | ▪aPS is projected to prevent 492 HIV infections, 759 deaths, and 6,198 DALYs over 10 years. ▪Estimated to avert 3.7% of infections, 2.6% of deaths, and 1.4% of DALYs vs. standard care. ▪Cost ranged from $2.5 to $3.5M over 5 years, below Kenya's GDP per capita | Not assessed |
| 68 | Wamuti B et al., 2022, Kenya | We estimated the costs of integrating aPS into routine HTS within  an ongoing aPS scale-up project in western Kenya | Study type: Micro costing analysis  Population: Index clients  Age: ≥15 years.  Sample: 1000 | LK | 20/1/2024 | JH, GC, JL | Not used | Not assessed | Not assessed | Not assessed | Not assessed | Not assessed | ▪Provider time: 25% on partner elicitation. ▪Incremental cost: $7,486 per facility/year. ▪Excluding NGO costs, $5,494 per facility/year.  ▪49% of the total cost on personnel.  ▪13% on transport. | Not assessed |
| 69 | Songane M et al., 2023, Mozambique | To estimate the cost-efficiency and effectiveness of community index testing and compare the HIV testing outputs with facility-based testing. | Study type: Cost analysis of community HIV index testing implemented. | LK | 29/1/2024 | JH, GC, JL | Not used | Not assessed | Not assessed | Not assessed | Not assessed | Not assessed | Of the total cost, 52% was on human resources, 28% on testing kits, and 8% on supplies.  $ 5.8 per client testing, $65.32 per new HIV diagnosis.  •Cost per infection averted per year was $1,813. | Not assessed |
| 70 | Cherutich P et al., 2018, Kenya | To conduct cost and budget impact analyses, respectively, of aPS compared to the current practice of HIV testing services (HTS) in Kisumu County, Kenya. | Study type: Budget Impact Analysis, Study population: New and old HIV cases | LK | 2/2/2024 | JH, GC, JL | Not used | Not assessed | Not assessed | Not assessed | Not assessed | Not assessed | ▪Total budget impact: Nurse model US$1,094,577 to US$1,767,863; CHW model US$1,191,185 to US$1,258,848 over 5 years, including aPS and ART costs. ▪Cost differences: CHW-based aPS costs 29-32% lower; Nurse model variations 1.9% to 6.6%. | Not assessed |
| 71 | Hu QH et al., China | To compare the effect of assisted vs. passive partner notification (PN) on uptake of HIV testing among sexual partners of newly HIV-diagnosed MSM | Randomized controlled trial (RCT); n = 187 MSM; Location: Shenyang, China | LK | 8/1/2024 | JH, GC, JL | HIV self-testing (HIVST) kits provided in assisted PN group | Passive PN: Self-referral; Assisted PN: HIVST kits and/or CBO referral. | Implicitly high in assisted PN group based on uptake; not quantitatively stated | 35% (assisted) vs 17% (passive) of index cases had partners tested; More partners tested in assisted PN (0.5 vs 0.2 per index case) | High acceptability inferred for assisted PN method | 49% of disclosed partners accessed HTS in assisted PN vs 28% in passive PN group | Implicitly high in assisted PN group based on uptake; not quantitatively stated | Facilitators: Community-based organization (CBO) outreach; HIVST kits; anonymous referral; Barriers: Not explicitly mentioned in abstract |
| 72 | Remera E et al., 2022, Rawanda | To assess the factors related to the HIV-positive outcome among older people (aged 50+) through index testing in Rwanda. | Quantitative observational study using logistic regression; 18,453 index cases; 31,227 partners notified and tested; Period: Oct 2018 â€“ Sept 2021 | LK | 19/1/2024 | JH, GC, JL | Facility-based rapid HIV testing (no mention of HIVST) | Client referral, provider referral, and dual referral strategies used to notify partners | Implied high based on scale and partner testing uptake | 3156 (10%) of tested partners were aged 50; 6% of them HIV-positive; 14.7% positivity among those notified by newly diagnosed index cases. | Not explicitly | Higher odds of HIV-positive result among female partners, those notified by newly diagnosed index cases, and those in age-discrepant relationships | Large-scale implementation reaching 31,227 partners; successful testing and identification of older HIV-positive individuals | Facilitators: Higher positivity among partners of newly diagnosed cases, age-discrepant relationships; Barriers not detailed |
| 73 | Goyette MS et al., 2018 | To assess whether history of intimate partner violence (IPV) modified APS effectiveness and relationship dissolution risk | Secondary analysis of cluster-randomized trial; immediate vs 6-week delayed APS; n = 1,119 index participants & 1,286 partners | LK | 13/1/2024 | JH, GC, JL | None (standard facility HIV testing) | Provider-assisted PN (immediate APS) vs delayed APS after 6 weeks | High acceptability; past IPV did not contraindicate APS | Testing, diagnosis, and linkage rates did not differ by IPV history. | High; no increased relationship dissolution among those with past IPV | Relationship dissolution rates similar | APS feasible and effective regardless of IPV history | Not explicitly reported; adverse events monitored |
| 74 | Maierhofer CN et al., Malawi | To characterize heterogeneity in effectiveness of network-based HIV testing interventions vs passive PN among persons with HIV | Randomized controlled trial; combination intervention vs pPN at 2 STI clinics in Lilongwe (2015-2019); binomial regression | LK | 16/1/2024 | JH, GC, JL | Standard rapid HIV testing (acute) | Combination: acute HIV testing + contract PN + social contact referral vs passive PN | Not explicitly reported | Greater PD for any contact by subgroup; no PD differences for new diagnoses; greater effect on HIV-negative contacts among younger & >1 partners. | Not explicitly reported | Efficacy measures as above; no adverse events | Network-based PNS feasible and efficacious, especially for women & previously diagnosed | Facilitators: female sex, prior diagnosis, younger age, multiple partners; Barriers: men, newly diagnosed, older, â‰¤1 partner |
| 75 | Onovo A et al., 2021, Nigeria | To describe feasibility and effectiveness of community-led index case testing for HIV diagnosis & linkage among KP partners | Programmatic study; index testing Oct 2018â€“Sep 2019 in nightclubs, hotels, community ART clinics; descriptive analysis | LK | 18/1/2024 | JH, GC, JL | Standard rapid HIV testing kits | Index testing by peer navigators; provider & passive referral; in-person & social network methods | High acceptability | 3,753 (49.7%) partners tested HIV-positive; 3,492 (93.0%) linked to care | High: 68.3% provider vs 30.1% passive referral | 8,989 contacts identified; high testing & linkage | Feasible & effective; reached many first-time testers & male KP | Facilitators: peer navigators; community-led outreach; Barriers: stigma & discrimination |
| 76 | Masters SH et al., Kenya | To determine whether secondary distribution of multiple HIV self-tests by ANC/postpartum women increases partner & couples testing vs invitation cards | Randomized clinical trial; n = 600 antenatal/postpartum women aged 18-39; Kisumu, Kenya; June 2015 Jan 2016 | LK | 27/2/2024 | JH, GC, JL | Oral-fluid HIV self-test kits (2 per participant) | HIVST group: 2 kits for partner or couples testing; Comparison: invitation card for clinic-based testing | High acceptability; no IPV reported | Partner testing: 90.8% vs 51.7% (diff 39.1%); Couples testing: 75.4% vs 33.2% | High acceptability; no adverse events | Same as outcomes above | Highly feasible; HIVST distribution led to >90% partner testing | Facilitators: privacy, convenience, couples testing; Barriers: self-report limitation |

**Key:** aPS: Assisted Partner Service, PN: partner notification, HIVST: HIV self-testing, HTS: HIV testing service, MSM: men who have sex with men, CHW: community health worker, ICTH: index case HIV/AIDS testing, WLIHV: women living with HIV, aPNS: assisted partner notification services, KP: key populations, VAPN: voluntary assisted partner notification services, PLHIV: people living with HIV, TW: transwomen, CBO: community-based organization, aPSST: assisted partner service self-testing, ART: antiretroviral therapy, PreP: pre-exposure prophylaxis, ANC: antenatal care, IPV: intimate partner violence, PRS: partner referral slip, FSW: female sex workers, rPS: Routine Partner Service, PC: postpartum care, RDT: rapid diagnostic kits, PWID: people who inject drugs, IAPN: information assisted partner notification, CHCT: Couples’ HIV counseling and testing, SOC: standard‐of‐care, DALY: Disability-adjusted life years
