# Supplementary material for: HIV self-testing and partner notification strategies for key populations in low- to upper-middle-income countries: A mixed-methods systematic review
Source: PLoS One. 2025 Dec 29;20(12):e0338639. doi: 10.1371/journal.pone.0338639 (PMC12747344; doi:10.1371/journal.pone.0338639)
Supplement: S6 Table — (DOCX) [file pone.0338639.s006.docx]

| **Table S6: Detailed quality appraisal** | | | | | | | | | | | | | | |
| --- | --- | --- | --- | --- | --- | --- | --- | --- | --- | --- | --- | --- | --- | --- |
| **Author/Year** | **KEY QUESTIONS** | **RELEVANCY** | **RELIABILITY** | | | **VALIDITY** | | | | **Applicability,** | **FINAL SCORE** |  | **Final Decision** |  |
|  |  | **Does the study address a topic(s) relevant to the issue under investigation?** | **a) Is the study presented clearly?** | **b) Are the research methodology and results clearly described?** | **c) Are ethics procedures described?** | **a) Is the study methodology appropriate for the scope of research?** | **b) Do they identify sources of bias and mitigation measures?** | **c) Are the authors’ conclusions explicit and transparent?** | **d) Can I be confident about the findings?** | **Can the results be applied within the scope of public health?** | **Yes (2) Not sure/unclear (1) No (0) MAX SCORE: 18/18** | **ROB** | **Will the paper be included or excluded?** |  |
| Maman S et al., 2017, Kenya | **Rating** | **Yes**/No & Not sure/unclear | **Yes**/No & Not sure/unclear | **Yes**/No & Not sure/unclear | **Yes**/No & Not sure/unclear | **Yes**/No & Not sure/unclear | Yes/No & **Not sure/unclear** | **Yes**/No & Not sure/unclear | **Yes**/No & Not sure/unclear | **Yes**/No & Not sure/unclear | 17 | Low-risk | Included |  |
|  | Reviewer's response | Identified gap, explores partner notification services (PNS) acceptability and feasibility. Consistent results with objectives. | Clear introduction, methods, results, discussion, organization, language, visuals, citations, conclusion, and coherence | Qualitative subset of larger cohort study. Structured sampling, interviews, analysis; results consistent with objectives, and reproducible methods. | Ethics approval reported | Methodology consistent with study objectives, purposive sampling, In-depth exploration. | Limitations: Self-reporting, selection bias Generalizability: Confined to FSW Missing interviews: Male partners, verification | Conclusion aligns with objectives | Findings consistency: Acceptability, the feasibility of HIVST Connection: Larger cohort study | The result can be useful for the promotion of HIVST to reach hidden key populations. |  |  |  |  |
|  | Reviewer's Score | 2 | 2 | 2 | 2 | 2 | 1 | 2 | 2 | 2 |  |  |  |  |
| Hershow RB et al., 2019, Malawi | **Rating** | **Yes**/No & Not sure/unclear | **Yes**/No & Not sure/unclear | **Yes**/No & Not sure/unclear | **Yes**/No & Not sure/unclear | **Yes**/No & Not sure/unclear | Yes/No & **Not sure/unclear** | **Yes**/No & Not sure/unclear | **Yes**/No & Not sure/unclear | **Yes**/No & Not sure/unclear | 17 | Low-risk | Included |  |
|  | Reviewer's response | Identified gap, assessing perspective on PNS using HIVST, explores male testing uptake, offers insights for testing initiatives. | Clear introduction, methods, results, discussion, organization, language, visuals, citations, conclusion, and coherence | Qualitative cross-sectional, structured sampling, interviews, analysis; results consistent with objectives, and reproducible methods. | Ethics approval reported | Methodology consistent with study objectives, purposive sampling, Qualitative in-depth interviews. | Study Design: Interviewer Bias Participants: Unclear Criteria Measurement: Self-Reporting Bias Factors: Critical Ones Omitted | Conclusion aligns with objectives | Authors' conclusions are clear and based on the results, ensuring transparency. | Results have implications for public health interventions and strategies. |  |  |  |  |
|  | Reviewer's Score | 2 | 2 | 2 | 2 | 2 | 1 | 2 | 2 | 2 |  |  |  |  |
| Dovel K et al., 2023, Malawi | **Rating** | **Yes**/No & Not sure/unclear | **Yes**/No & Not sure/unclear | **Yes**/No & Not sure/unclear | **Yes**/No & Not sure/unclear | **Yes**/No & Not sure/unclear | Yes/No & **Not sure/unclear** | **Yes**/No & Not sure/unclear | **Yes**/No & Not sure/unclear | **Yes**/No & Not sure/unclear | 17 | Low-risk | Included |  |
|  | Reviewer's response | Identified gap, assessing the effectiveness of index testing for PNS. Result relevant to the issues. | Clear introduction, methods, results, discussion, organization, language, figures/tables, citations, conclusion, and overall coherence. | Quantitative RCT study, structured sampling, interviews, Binomial regression, stratified analysis, results consistent with objectives, and reproducible methods. | Ethics approval reported | Methodology consistent with study objectives, Simple randomization, surveys conducted. | Bias: Incomplete surveys, reporting reliance, small sample. Mitigation: Reduce attrition, and involve partners. Use larger, diverse samples. | Clear conclusions, detailed discussion, positive outcomes, stated primary and secondary outcomes. | Rigorous methodology, clear criteria, appropriate statistics | To enhance HIV testing for timely care and support. |  |  |  |  |
|  | Reviewer's Score | 2 | 2 | 2 | 2 | 2 | 1 | 2 | 2 | 2 |  |  |  |  |
| Mutale W et al., 2021, Zambia | **Rating** | **Yes**/No & Not sure/unclear | **Yes**/No & Not sure/unclear | **Yes**/No & Not sure/unclear | **Yes**/No & Not sure/unclear | **Yes**/No & Not sure/unclear | Yes/No & **Not sure/unclear** | **Yes**/No & Not sure/unclear | **Yes**/No & Not sure/unclear | **Yes**/No & Not sure/unclear | 17 | Low-risk | Included |  |
|  | Reviewer's response | Identified gap, studying male PNS with HIVST; Results relevant to the issues. suitable RCT for evaluation. | Clear introduction, easy conduct. Well-presented outcomes, and reproducible. | Two parallel RCT study, structured sampling, interviews, analysis; results consistent with objectives, and reproducible methods. | Ethics approval reported | Methodology consistent with study objectives, randomization done, surveys conducted. | Possible bias: Unmasked RCT, no concealment. Noted biases: Recall, reporting. | Findings presented clearly; limitations cited. | Valuable insights, interpret cautiously due to limitations. | Study results impact HIV testing rates for male partners of pregnant women. |  |  |  |  |
|  | Reviewer's Score | 2 | 2 | 2 | 2 | 2 | 1 | 2 | 2 | 2 |  |  |  |  |
| Thirumurthy H et al., 2021, Kenya | **Rating** | **Yes**/No & Not sure/unclear | **Yes**/No & Not sure/unclear | **Yes**/No & Not sure/unclear | **Yes**/No & Not sure/unclear | **Yes**/No & Not sure/unclear | Yes/No & **Not sure/unclear** | **Yes**/No & Not sure/unclear | **Yes**/No & Not sure/unclear | **Yes**/No & Not sure/unclear | 17 | Low-risk | Included |  |
|  | Reviewer's response | Identified gap, assessing the effectiveness of women's sustained access to HIVST for couple testing, Results relevant to the issues. suitable pair-matched cluster-RCT. | The study introduction is clear, and all components described well. | A cluster-RCT study, structured sampling, interviews, analysis; results consistent with objectives, and reproducible methods. | Ethics approval reported | Research questions aligns with design, sued pair-matched cluster-randomized trial design, informed consent, survey conducted. | Participant bias noted, baseline data missing. Analysis controls for bias, chance findings possible. Results consistent with objectives. | Findings presented clearly; limitations cited. | Robust methods and clear findings | To increase the coverage of testing and treatment. |  |  |  |  |
|  | Reviewer's Score | 2 | 2 | 2 | 2 | 2 | 1 | 2 | 2 | 2 |  |  |  |  |
| Agot K et al., 2018, Kenya | **Rating** | **Yes**/No & Not sure/unclear | **Yes**/No & Not sure/unclear | **Yes**/No & Not sure/unclear | **Yes**/No & Not sure/unclear | **Yes**/No & Not sure/unclear | Yes/No & **Not sure/unclear** | **Yes**/No & Not sure/unclear | **Yes**/No & Not sure/unclear | **Yes**/No & Not sure/unclear | 17 | Low-risk | Included |  |
|  | Reviewer's response | Identified gap, distribution of HIVST for sexual partners, analysis from larger cohort study, results consistent with objectives. | The study introduction is clear, and all components described well. | Observational cohort quantitative study, structured sampling, interviews, descriptive, chi-square; results consistent with objectives, and reproducible methods. | Ethics approval reported | Research question aligns with design, convenience and purposive sampling, survey and testing conducted. | Potential bias: Self-report, loss to follow-up, no confounder control, limited generalizability. Mitigations: SOPs, training, informed consent. | Findings presented clearly; limitations cited. | Yes | Yes |  |  |  |  |
|  | Reviewer's Score | 2 | 2 | 2 | 2 | 2 | 1 | 2 | 2 | 2 |  |  |  |  |
| Myers RS et al., 2016, Mozambique | **Rating** | **Yes**/No & Not sure/unclear | **Yes**/No & Not sure/unclear | **Yes**/No & Not sure/unclear | **Yes**/No & Not sure/unclear | **Yes**/No & Not sure/unclear | Yes/No & **Not sure/unclear** | **Yes**/No & Not sure/unclear | **Yes**/No & Not sure/unclear | **Yes**/No & Not sure/unclear | 15 | Low-risk | Included |  |
|  | Reviewer's response | Identified gap, assessing acceptability and effectiveness of assisted PNS, results relevant with issues. | The study introduction is clear, and all components described well. | Observational cohort study, structured sampling, interviews, analysis; results consistent with objectives, and reproducible methods. | Ethics approval reported | Research question aligns with design, convenience sampling, structured Interview and testing conducted. | Study Bias: potential bias, Self-reporting may introduce bias. 65%: Reported HIV disclosure before APS Incomplete: Partner testing, self-reporting, APS, potential bias | Findings presented clearly; limitations cited. | Methodological limitations noted, results align with literature. Discrepancies with previous studies unclear. | Results: Enhance HIV testing Partner notification: Evidence-based strategies Linkage to care: Implementation |  |  |  |  |
|  | Reviewer's Score | 2 | 2 | 2 | 2 | 1 | 1 | 2 | 1 | 2 |  |  |  |  |
| Xiao WJ et al., 2020, China | **Rating** | **Yes**/No & Not sure/unclear | **Yes**/No & Not sure/unclear | **Yes**/No & Not sure/unclear | **Yes**/No & Not sure/unclear | **Yes**/No & Not sure/unclear | Yes/No & **Not sure/unclear** | **Yes**/No & Not sure/unclear | **Yes**/No & Not sure/unclear | **Yes**/No & Not sure/unclear | 16 | Low-risk | Included |  |
|  | Reviewer's response | Identified gap, evaluating the PNS Sexual network distribution of HIVST for MSM, | The study introduction is clear, and all components described well. | Observational cohort study, structured sampling, interviews, analysis; results consistent with objectives, and reproducible methods. | No mention of ethics. | Research question aligns with design, convenience sampling, Structured Interviews, questionaries, mobile device surveys, testing. | Small sample size, potential social desirability bias. Relevant factors measured, consistent with the study. No mention of chance ruling out. | Study conclusive, reports key findings, includes limitations. | Valuable insights, interpret results cautiously due to limitations. | Implications for public health: Improved HIV testing, reduced transmission. |  |  |  |  |
|  | Reviewer's Score | 2 | 2 | 2 | 2 | 2 | 1 | 2 | 1 | 2 |  |  |  |  |
| Boye S et al., 2021, Mali | **Rating** | **Yes**/No & Not sure/unclear | **Yes**/No & Not sure/unclear | **Yes**/No & Not sure/unclear | **Yes**/No & Not sure/unclear | **Yes**/No & Not sure/unclear | Yes/No & **Not sure/unclear** | **Yes**/No & Not sure/unclear | **Yes**/No & Not sure/unclear | **Yes**/No & Not sure/unclear | 17 | Low-risk | Included |  |
|  | Reviewer's response | Identified gap, exploring the challenges in HIVST distribution, results consistent with issues. | The study introduction is clear, and all components described well. | Cross-sectional quantitative study, structured sampling, interviews, analysis; information source clear, results consistent with objectives, and reproducible methods. | Ethics approval reported | Research question aligns with design, convenience Sampling, semi-structured Interviews, and observation. | Bias: Implicit inclusion criteria. Sample: Lack of representativeness. Analysis: Thematic coding. Generalizability: Single-site limitation. | Findings presented clearly, limitations cited. | Study findings valid, align with objectives, limitations noted. | Results relevant for informing HIV testing strategies among PLHIV partners. |  |  |  |  |
|  | Reviewer's Score | 2 | 2 | 2 | 2 | 2 | 1 | 2 | 2 | 2 |  |  |  |  |
| Nguyen V et al., 2019, Vietnam | **Rating** | **Yes**/No & Not sure/unclear | **Yes**/No & Not sure/unclear | **Yes**/No & Not sure/unclear | **Yes**/No & Not sure/unclear | **Yes**/No & Not sure/unclear | Yes/No & **Not sure/unclear** | **Yes**/No & Not sure/unclear | **Yes**/No & Not sure/unclear | **Yes**/No & Not sure/unclear | 17 | Low-risk | Included |  |
|  | Reviewer's response | Gaps identified, pilot study to assess the community-led HIV testing services (HTS) for PNS, results consistent with issues. | The study introduction is clear, and all components described well. | Quantitative study with structured sampling, interviews, clear data sources; results align with objectives; reproducible | Ethics approval reported | Research question aligns with design, convenience sampling, client information forms, observation and testing. | Challenges in aPN implementation; difficulty reaching casual partners; no biases identified; calls for additional strategies and bias awareness | Findings presented clearly; limitations cited. | Methods: Relevant to objectives Discussion: Grounded in findings, relevant literature | aPN effective for HIV case-finding in Vietnam. Advocate integration into community-led testing. Benefits public health efforts. |  |  |  |  |
|  | Reviewer's Score | 2 | 2 | 2 | 2 | 2 | 1 | 2 | 2 | 2 |  |  |  |  |
| Onovo A et al., 2022, Nigeria | **Rating** | **Yes**/No & Not sure/unclear | **Yes**/No & Not sure/unclear | **Yes**/No & Not sure/unclear | **Yes**/No & Not sure/unclear | **Yes**/No & Not sure/unclear | Yes/No & **Not sure/unclear** | **Yes**/No & Not sure/unclear | **Yes**/No & Not sure/unclear | **Yes**/No & Not sure/unclear | 16 | Low-risk | Included |  |
|  | Reviewer's response | Gaps identified, HTS among key populations (KP), results consistent with issues. | Clear intro, methods, results, discussion, organization, language, visuals, citations, and coherence. | Retrospective cohort study with structured sampling, interviews, clear data sources; results align with objectives; reproducible. | Ethics approval reported | Research question aligns with design, convenience sampling, PNS register, client intake forms and testing. | Bias: Not explicitly identified Mitigation measures: Not specified | Authors' conclusions transparent, align with observed patterns and themes. | Methods: Relevant to objectives Discussion: Grounded in findings, relevant literature | Findings support public health; improve HIV testing, prevention, treatment; target key populations; aid epidemic control |  |  |  |  |
|  | Reviewer's Score | 2 | 2 | 1 | 2 | 2 | 1 | 2 | 2 | 2 |  |  |  |  |
| Oldenburg CE et al., 2018, Zambia | **Rating** | **Yes**/No & Not sure/unclear | **Yes**/No & Not sure/unclear | **Yes**/No & Not sure/unclear | **Yes**/No & Not sure/unclear | **Yes**/No & Not sure/unclear | Yes/No & **Not sure/unclear** | **Yes**/No & Not sure/unclear | **Yes**/No & Not sure/unclear | **Yes**/No & Not sure/unclear | 18 | Low-risk | Included |  |
|  | Reviewer's response | Gap identified, assessing the effect of HIVST PNS among female sex workers, results consistent with issues.  Research design-three-arm cluster RCT | The study aim is clear and well described study conduct and overall coherence. | Prospective cohort quantitative study with structured sampling, interviews, clear data sources; results align with objectives; reproducible | Ethics approval reported | Design fits question; convenience sampling; CAPI, baseline surveys, registers, and testing used | Social Desirability Bias, Masking Effects of Peer Educator Intervention. | Clear, transparent conclusions connected to study results. | Relevant methods: discussion grounded in findings, literature. | HIV self-testing reduces partners Advocates tailored interventions Expands testing access for HIV prevention |  |  |  |  |
|  | Reviewer's Score | 2 | 2 | 2 | 2 | 2 | 2 | 2 | 2 | 2 |  |  |  |  |
| Dvora L. et al., 2022, South Africa | **Rating** | **Yes**/No & Not sure/unclear | **Yes**/No & Not sure/unclear | **Yes**/No & Not sure/unclear | **Yes**/No & Not sure/unclear | **Yes**/No & Not sure/unclear | Yes/No & **Not sure/unclear** | **Yes**/No & Not sure/unclear | **Yes**/No & Not sure/unclear | **Yes**/No & Not sure/unclear | 18 | Low-risk | Included |  |
|  | Reviewer's response | Clear justification, assessing the feasibility, and effectiveness of HIVST for PNS, results consistent with issues. | Clear introduction, methods, results, discussion, organization, language, figures/tables, citations, conclusion, and overall coherence. | A pilot RCT study, structured sampling, interviews, analysis; information source clear, results consistent with objectives, and reproducible methods. | Ethics approval reported | Research question aligns with design, random sampling, surveys and testing. | Bias: Busy healthcare settings, social desirability. Verification: HIVST kit photo evidence. Analysis: Adjusted for baseline and confounders. Unlikely due to chance. | Findings presented clearly; limitations cited. | Confident-Methods are relevant to study objectives and discussion well-grounded with the findings and relevant literature. | Women distributing HIV self-tests Increases men's testing Promising for high-prevalence areas |  |  |  |  |
|  | Reviewer's Score | 2 | 2 | 2 | 2 | 2 | 2 | 2 | 2 | 2 |  |  |  |  |
| Zishiri V et al., 2022, South Africa | **Rating** | **Yes**/No & Not sure/unclear | **Yes**/No & Not sure/unclear | **Yes**/No & Not sure/unclear | **Yes**/No & Not sure/unclear | **Yes**/No & Not sure/unclear | Yes/No & **Not sure/unclear** | **Yes**/No & Not sure/unclear | **Yes**/No & Not sure/unclear | **Yes**/No & Not sure/unclear | 18 | Low-risk | Included |  |
|  | Reviewer's response | Clear justification, addressing low testing rates in men through PNS, results relevant to issues. | The study aim is clear and well described study conduct and overall coherence. | Observational cohort mixed-method study with structured sampling, clear data sources; results align with objectives; reproducible. | Ethics approval reported | Research question aligns with study design.  Analysis accounts for key theoretical factors. | Clear criteria Standardized data collection Statistical control for confounding variables | Transparent conclusions with key finding and imitations. | Confident in methods Grounded discussion Relevant literature | Valuable insights Increased testing rates Improved linkage to care |  |  |  |  |
|  | Reviewer's Score | 2 | 2 | 2 | 2 | 2 | 2 | 2 | 2 | 2 |  |  |  |  |
| Choko AT et al., 2021, Malawi | **Rating** | **Yes**/No & Not sure/unclear | **Yes**/No & Not sure/unclear | **Yes**/No & Not sure/unclear | **Yes**/No & Not sure/unclear | **Yes**/No & Not sure/unclear | Yes/No & **Not sure/unclear** | **Yes**/No & Not sure/unclear | **Yes**/No & Not sure/unclear | **Yes**/No & Not sure/unclear | 18 | Low-risk | Included |  |
|  | Reviewer's response | Justification clear, evaluating HIVST for PNS, improved testing for male partners, and sexual contacts, results relevant to the issues. | The study introduction is clear, and all components described well. | A cluster RCT study, structured sampling, criteria, clear information source, results aligned with objectives, reproducible methods. | Ethics approval reported | Design fits questions; random sampling, surveys, testing; methods align with model; controls included | High cluster variability; reporting bias; HIV test errors; HIVST misclassification; supply issues affect VMMC. | Transparent conclusions with key finding and imitations. | Study methods are relevant to objectives, well-grounded in findings and literature | HIV-positive women distributing HIVST boosts male testing; promising for high-prevalence areas |  |  |  |  |
|  | Reviewer's Score | 2 | 2 | 2 | 2 | 2 | 2 | 2 | 2 | 2 |  |  |  |  |
| Zewdie K et al., 2022, Kenya | **Rating** | **Yes**/No & Not sure/unclear | **Yes**/No & Not sure/unclear | **Yes**/No & Not sure/unclear | **Yes**/No & Not sure/unclear | **Yes**/No & Not sure/unclear | Yes/No & **Not sure/unclear** | **Yes**/No & Not sure/unclear | **Yes**/No & Not sure/unclear | **Yes**/No & Not sure/unclear | 18 | Low-risk | Included |  |
|  | Reviewer's response | Justification clear, assessing feasibility of HIVST for PNS, results consistent with issues.   Design: Observational cohort, comparing testing approaches. | The study aim is clear and well described study conduct and overall coherence. | Observational cohort, structured sampling, criteria, clear information source, results aligned with objectives, reproducible methods. | Ethics approval reported | Research question congruent with the study design, consecutive sampling, survey. And testing. | Measurement: Reliability, validity, social desirability bias. Outcome: Short follow-up. External Validity: Generalization challenge. | Results conclusive within its objectives. | Study methods are relevant to objectives, well-grounded in findings and relevant literature. | HIV self-testing in Kenya increases partner testing, vital for public health |  |  |  |  |
|  | Reviewer's Score | 2 | 2 | 2 | 2 | 2 | 2 | 2 | 2 | 2 |  |  |  |  |
| Luo MY et al., 2020, China | **Rating** | **Yes**/No & Not sure/unclear | **Yes**/No & Not sure/unclear | **Yes**/No & Not sure/unclear | **Yes**/No & Not sure/unclear | **Yes**/No & Not sure/unclear | Yes/No & **Not sure/unclear** | **Yes**/No & Not sure/unclear | **Yes**/No & Not sure/unclear | **Yes**/No & Not sure/unclear | 16 | Low-risk | Included |  |
|  | Reviewer's response | Justification clear, HIV testing uptake and yield among sexual partners of HIV-positive MSM, results relevant to issues. | Rationale clear, conduct straightforward, design cross-sectional. All pertinent data included, reproducible. | A cross-sectional pilot study, structured sampling, criteria, clear information source, results aligned with objectives, reproducible methods. | Ethical approval (Zhejiang CDC); written consent obtained. | Design fits question; lacks conceptual model and control; random sampling, surveys, and testing conducted | Self-report data used, potentially biased. Participant selection bias by settlement and education. Limited enrolment of newly diagnosed HIV-positive MSM and partners. | Transparent conclusions with key finding and imitations. | Methods align with study objectives, grounded in relevant literature, instilling confidence in the findings. | Study offers new insights for HIV testing strategies among newly diagnosed MSM partners. |  |  |  |  |
|  | Reviewer's Score | 2 | 2 | 2 | 2 | 1 | 1 | 2 | 2 | 2 |  |  |  |  |
| Agot K et al., 2020, Kenya | **Rating** | **Yes**/No & Not sure/unclear | **Yes**/No & Not sure/unclear | **Yes**/No & Not sure/unclear | **Yes**/No & Not sure/unclear | **Yes**/No & Not sure/unclear | Yes/No & **Not sure/unclear** | **Yes**/No & Not sure/unclear | **Yes**/No & Not sure/unclear | **Yes**/No & Not sure/unclear | 16 | Low-risk | Included |  |
|  | Reviewer's response | Justification clear, exploring the experiences of women when distributing HIVST for PNS, results consistent with issues. | The study introduction, results, discussion, and overall coherence. | Sub study within an RCT, Qualitative/mixed-methods, structured sampling, criteria, clear information source, results aligned with objectives, reproducible methods. | Informed consent mentioned | Design fits questions; lacks theory model, inter-coder reliability, and details; random sampling and survey used. | Limited discussion on bias sources and mitigation. Some limitations acknowledged. | Transparent conclusions with key finding and imitations. | Methods are relevant to study objectives and discussion well-grounded with the findings and relevant literature. | Study yields insights into HIV testing strategies for partners of newly diagnosed MSM. |  |  |  |  |
|  | Reviewer's Score | 2 | 2 | 2 | 2 | 1 | 1 | 2 | 2 | 2 |  |  |  |  |
| Mujugira A et al., 2023, Uganda | **Rating** | **Yes**/No & Not sure/unclear | **Yes**/No & Not sure/unclear | **Yes**/No & Not sure/unclear | **Yes**/No & Not sure/unclear | **Yes**/No & Not sure/unclear | Yes/No & **Not sure/unclear** | **Yes**/No & Not sure/unclear | **Yes**/No & Not sure/unclear | **Yes**/No & Not sure/unclear | 17 | Low-risk | Included |  |
|  | Reviewer's response | Justification clear, evaluate the benefits and risks of secondary distribution of HIVST from PWLHIV, results consistent with objectives. | Introduction clear, well-described study conduct. | A RCT study, structured sampling, criteria, clear information source, results aligned with objectives, reproducible methods. | Ethics approval reported | Research question consistent with design, random sampling, HIVST kit distribution, surveys, and appropriate control included. | Bias sources mentioned, mitigation lacking. Sample size limit generalizability. Potential underestimation in self-testing arm. Resource limitations affect outcomes. | Transparent conclusions with key finding and imitations. | methods aligned with objectives. Discussion grounded in findings and literature. | HIV self-testing by pregnant women didn't increase partner testing. |  |  |  |  |
|  | Reviewer's Score | 2 | 2 | 2 | 2 | 2 | 1 | 2 | 2 | 2 |  |  |  |  |
| Ye ZH et al., 2023, China | **Rating** | **Yes**/No & Not sure/unclear | **Yes**/No & Not sure/unclear | **Yes**/No & Not sure/unclear | **Yes**/No & Not sure/unclear | **Yes**/No & Not sure/unclear | Yes/No & **Not sure/unclear** | **Yes**/No & Not sure/unclear | **Yes**/No & Not sure/unclear | **Yes**/No & Not sure/unclear | 17 | Low-risk | Included |  |
|  | Reviewer's response | Justification clear, results applicable, population and setting similar, RCT design appropriate. | Clear introduction, well-described conduct, relevant results, conflict of interest disclosed. | A multi-centric RCT study, structured sampling, criteria, clear information source, results aligned with objectives, reproducible methods. | Ethics approval reported | Design fits question; random sampling, testing, surveys, controls included lacks theoretical linkage. | Self-reported data bias, randomization bias, blinding bias, sample size bias, mitigation measures not specified. | Transparent conclusions with key finding and imitations. | Relevant methods, grounded discussion, relevant literature. | aPS expansion, social harm, staff training, biases, transparent interpretation. |  |  |  |  |
|  | Reviewer's Score | 2 | 2 | 2 | 2 | 2 | 1 | 2 | 2 | 2 |  |  |  |  |
| **Chen et al., 2019, Malawi** | **Rating** | **Yes**/No & Not sure/unclear | **Yes**/No & Not sure/unclear | **Yes**/No & Not sure/unclear | **Yes**/No & Not sure/unclear | **Yes**/No & Not sure/unclear | Yes/No & **Not sure/unclear** | **Yes**/No & Not sure/unclear | **Yes**/No & Not sure/unclear | **Yes**/No & Not sure/unclear | 18 | Low-risk | Included |  |
|  | Reviewer's response | Justification clear, study aimed to determine the impact of network-based testing and PNS, results relevant to issues. | The study introduction, results, discussion, and overall coherence. | A RCT study, structured sampling, criteria, clear information source, results aligned with objectives, reproducible methods. | Ethics approval reported | Design fits question; RCT data; consecutive sampling; relevant controls, surveys, and testing included | Appropriate design, participant selection, and analysis. Reliable measurement and data sources. Comprehensive factors considered. Consistent results, ruling out chance findings. | Transparent conclusions with key finding and imitations. | Findings are reliable; methodological flaws are addressed, and discrepancies with existing literature are explained. | Public health implications require considering outcomes, applicability, harms, benefits, and stakeholders |  |  |  |  |
|  | Reviewer's Score | 2 | 2 | 2 | 2 | 2 | 2 | 2 | 2 | 2 |  |  |  |  |
| Madsen T et al., 2020, Guinea-Bissau | **Rating** | **Yes**/No & Not sure/unclear | **Yes**/No & Not sure/unclear | **Yes**/No & Not sure/unclear | **Yes**/No & Not sure/unclear | **Yes**/No & Not sure/unclear | Yes/No & **Not sure/unclear** | **Yes**/No & Not sure/unclear | **Yes**/No & Not sure/unclear | **Yes**/No & Not sure/unclear | 17 | Low-risk | Included |  |
|  | Reviewer's response | Justification clear, assessing the effectiveness of index testing, results relevant to issues. | The study introduction, results, discussion, and overall coherence. | Quasi-experimental study, structured sampling, criteria, clear information source, results aligned with objectives, reproducible methods. | Ethics approval reported | Design fits questions; lacks theory model, confounder adjustment, and linkage; convenience sampling; surveys conducted | Selection bias, control group absence, limited generalizability, no mitigation mentioned | Transparent conclusions with key finding and imitations. | Aligned of methods with study objectives and literature. | Results applicable for informing HIV prevention and care strategies in similar healthcare settings. |  |  |  |  |
|  | Reviewer's Score | 2 | 2 | 2 | 2 | 2 | 1 | 2 | 2 | 2 |  |  |  |  |
| Cherutich P et al., 2017, Kenya | **Rating** | **Yes**/No & Not sure/unclear | **Yes**/No & Not sure/unclear | **Yes**/No & Not sure/unclear | **Yes**/No & Not sure/unclear | **Yes**/No & Not sure/unclear | Yes/No & **Not sure/unclear** | **Yes**/No & Not sure/unclear | **Yes**/No & Not sure/unclear | **Yes**/No & Not sure/unclear | 17 | Low-risk | Included |  |
|  | Reviewer's response | Justification clear, assessing whether or not aPNS increases HIV testing/care/treatment, results relevant to issues. | The study introduction, results, discussion, and overall coherence. | A cluster RCT study, structured sampling, criteria, clear information source, results aligned with objectives, reproducible methods. | Ethics approval reported | Research question aligns with design, cluster random sampling, surveys and testing. | Data collection: Potential self-reporting bias. Study bias: Exclusion of high IPV risk participants. | Transparent conclusions with key finding and imitations. | Methods relevant, grounded in objectives and literature. | Assisted partner services increase partner HIV testing; support public health interventions |  |  |  |  |
|  | Reviewer's Score | 2 | 2 | 2 | 2 | 2 | 1 | 2 | 2 | 2 |  |  |  |  |
| Goyette MS et al., 2018, Kenya | **Rating** | **Yes**/No & Not sure/unclear | **Yes**/No & Not sure/unclear | **Yes**/No & Not sure/unclear | **Yes**/No & Not sure/unclear | **Yes**/No & Not sure/unclear | Yes/No & **Not sure/unclear** | **Yes**/No & Not sure/unclear | **Yes**/No & Not sure/unclear | **Yes**/No & Not sure/unclear | 17 | Low-risk | Included |  |
|  | Reviewer's response | Clear justification, assessing the intimate partner violence (IPV) for PNS, results consistent with issues. | Study objectives clear, detailed protocols may be needed. | A RCT study, structured sampling, criteria, clear information source, results aligned with objectives, reproducible methods. | Ethics approval reported | Research question aligns with design, cluster random sampling, surveys and testing. | Sample Size: Few IPV events, limits generalizability. Measurement: Single IPV questions at enrolment. Stats Analysis: Limited by few IPV events. External Validity: Exclusion criteria restrict generalization. | Transparent conclusions with key finding and imitations. | Methods are relevant to study objectives and discussion well-grounded with the findings and relevant literature. | APS boosts partner testing, even with IPV history. Supports APS for HIV care and prevention in like settings. |  |  |  |  |
|  | Reviewer's Score | 2 | 2 | 2 | 2 | 2 | 1 | 2 | 2 | 2 |  |  |  |  |
| Masyuko SJ et al., 2019, Kenya | **Rating** | **Yes**/No & Not sure/unclear | **Yes**/No & Not sure/unclear | **Yes**/No & Not sure/unclear | **Yes**/No & Not sure/unclear | **Yes**/No & Not sure/unclear | Yes/No & **Not sure/unclear** | **Yes**/No & Not sure/unclear | **Yes**/No & Not sure/unclear | **Yes**/No & Not sure/unclear | 17 | Low-risk | Included |  |
|  | Reviewer's response | Clear justification-assessing aPS effectiveness results consistent with objectives.  Methodology: Cluster-randomized trial design. | The study introduction, results, discussion, and overall coherence, replication feasible. | A cluster RCT study, structured sampling, criteria, clear information source, results aligned with objectives, reproducible methods. | Ethics approval reported | Research question aligns with design, cluster random sampling, surveys and testing. |  | Transparent conclusions with key finding and imitations. | Methods aligned with objectives, grounded in findings and literature. | Assisted PN boosts testing, diagnoses, and treatment linkage; informs public health for underserved groups. |  |  |  |  |
|  | Reviewer's Score | 2 | 2 | 2 | 2 | 2 | 1 | 2 | 2 | 2 |  |  |  |  |
| Culbert GJ et al., 2020, Indonesia | **Rating** | **Yes**/No & Not sure/unclear | **Yes**/No & Not sure/unclear | **Yes**/No & Not sure/unclear | **Yes**/No & Not sure/unclear | **Yes**/No & Not sure/unclear | Yes/No & **Not sure/unclear** | **Yes**/No & Not sure/unclear | **Yes**/No & Not sure/unclear | **Yes**/No & Not sure/unclear | 17 | Low-risk | Included |  |
|  | Reviewer's response | Justification clear, assessing aPNS in prisons, results relevant to issues. | The study introduction, results, discussion, and overall coherence, replication feasible. | A RCT study, structured sampling, criteria, clear information source, results aligned with objectives, reproducible methods. | Ethics approval reported | Research question aligns with design, cluster random sampling, surveys and testing. | Control group missing Self-report bias Short follow-up duration Limited generalizability Resource constraints | Authors' conclusions transparently detail findings, limitations, and implications. | Methods align with objectives and literature, grounding discussions effectively. | Shows Impart APN effectiveness; boosts testing and notification; informs prison HIV prevention |  |  |  |  |
|  | Reviewer's Score | 2 | 2 | 2 | 2 | 2 | 1 | 2 | 2 | 2 |  |  |  |  |
| Uma TH et al., 2023, Ethiopia | **Rating** | **Yes**/No & Not sure/unclear | **Yes**/No & Not sure/unclear | **Yes**/No & Not sure/unclear | **Yes**/No & Not sure/unclear | **Yes**/No & Not sure/unclear | Yes/No & **Not sure/unclear** | **Yes**/No & Not sure/unclear | **Yes**/No & Not sure/unclear | **Yes**/No & Not sure/unclear | 17 | Low-risk | Included |  |
|  | Reviewer's response | Justification clear, assessing factors associated with partner and family-based index case, Results relevant to issues. | The study introduction is clear, and all components described well. | A cross-sectional quantitative study, structured sampling, criteria, clear information source, results aligned with objectives, reproducible methods. | Ethics approval reported | Research question aligns with design, systematic random sampling, surveys and testing. | Selection bias from health facility-based sample. Potential social desirability bias in self-reported | Authors' conclusions transparently detail findings, limitations, and implications. | Confident-Methods are relevant to study objectives and discussion well-grounded with the findings and relevant literature. | Inform public health priorities. Target ART support. Enhance counselling services. Facilitate testing and disclosure. |  |  |  |  |
|  | Reviewer's Score | 2 | 2 | 2 | 2 | 2 | 1 | 2 | 2 | 2 |  |  |  |  |
| Tih PM et al., 2019, Cameroon | **Rating** | **Yes**/No & Not sure/unclear | **Yes**/No & Not sure/unclear | **Yes**/No & Not sure/unclear | **Yes**/No & Not sure/unclear | **Yes**/No & Not sure/unclear | Yes/No & **Not sure/unclear** | **Yes**/No & Not sure/unclear | **Yes**/No & Not sure/unclear | **Yes**/No & Not sure/unclear | 17 | Low-risk | Included |  |
|  | Reviewer's response | Justification clear, describing the large-scale implementation of aPNS, results relevant to issues. | The study introduction is clear, and all components described well. | A cross-sectional quantitative study, structured sampling, criteria, clear information source, results aligned with objectives, reproducible methods. | Ethics approval reported | Research question aligns with design, sampling technique not mentioned, structured questionnaire and testing. | Potential biases due to real-world implementation conditions. Resource Limitations: | Authors' conclusions transparently detail findings, limitations, and implications. | Confident-Methods are relevant to study objectives and discussion well-grounded with the findings and relevant literature. | Program success in identifying PLHIV, counselling provision, addressing social harms, public health and policy implications. |  |  |  |  |
|  | Reviewer's Score | 2 | 2 | 2 | 2 | 2 | 1 | 2 | 2 | 2 |  |  |  |  |
| Chelogoi E et al. | **Rating** | **Yes**/No & Not sure/unclear | **Yes**/No & Not sure/unclear | **Yes**/No & Not sure/unclear | **Yes**/No & Not sure/unclear | **Yes**/No & Not sure/unclear | Yes/No & **Not sure/unclear** | **Yes**/No & Not sure/unclear | **Yes**/No & Not sure/unclear | **Yes**/No & Not sure/unclear | 17 | Low-risk | Included |  |
|  | Reviewer's response | Justification clear, assessing the barriers to aPNS, results consistent with objectives. | The study introduction is clear, and all components described well. | A cross-sectional quantitative study, structured sampling, criteria, clear information source, results aligned with tables, reproducible methods. | Ethics approval reported | Research question aligns with design, stratified and simple random sampling, survey and testing. | Measurement and Data Collection: Social desirability impacts accuracy. Statistical and Analytical Issues: Limited depth from quantitative data. Study Bias: Recall bias due to time lag. | Authors' conclusions transparently detail findings, limitations, and implications. | Methods are relevant to study objectives and discussion well-grounded with the findings and relevant literature. | Findings inform public health strategies, enhance HIV testing coverage, target interventions, consider study-identified factors. |  |  |  |  |
|  | Reviewer's Score | 2 | 2 | 2 | 2 | 2 | 1 | 2 | 2 | 2 |  |  |  |  |
| Shamu S et al., 2019, South Africa | **Rating** | **Yes**/No & Not sure/unclear | **Yes**/No & Not sure/unclear | **Yes**/No & Not sure/unclear | **Yes**/No & Not sure/unclear | **Yes**/No & Not sure/unclear | Yes/No & **Not sure/unclear** | **Yes**/No & Not sure/unclear | **Yes**/No & Not sure/unclear | **Yes**/No & Not sure/unclear | 17 | Low-risk | Included |  |
|  | Reviewer's response | Justification clear, assessing the factors obstructing aPNS, results consistent with objectives. | The study introduction is clear, and all components described well. | Cross-sectional quantitative study with structured sampling, clear data sources, proportions, Z-tests, ANOVA, Tukey’s HSD; results align with objectives; reproducible | Ethics approval reported | Research question aligns with design, stratified and simple random sampling, survey and testing. | Lack of detailed data on linkage to care by testing modality. Reliance on programmatic data not tailored for research. Missing individual-level data on potential harm and refusal rates. | Authors' conclusions transparently detail findings, limitations, and implications. | Methods are relevant to study objectives and discussion well-grounded with the findings and relevant literature. | Findings support index testing for HIV; inform public health; broader outcomes and stakeholder views need consideration |  |  |  |  |
|  | Reviewer's Score | 2 | 2 | 2 | 2 | 2 | 1 | 2 | 2 | 2 |  |  |  |  |
| Andriyanto A et al., 2023, Indonesia | **Rating** | **Yes**/No & Not sure/unclear | **Yes**/No & Not sure/unclear | **Yes**/No & Not sure/unclear | **Yes**/No & Not sure/unclear | **Yes**/No & Not sure/unclear | Yes/No & **Not sure/unclear** | **Yes**/No & Not sure/unclear | **Yes**/No & Not sure/unclear | **Yes**/No & Not sure/unclear | 16 | Low-risk | Included |  |
|  | Reviewer's response | Justification clear, presenting the approaches for PNS, results relevant to issues. | The study aim is clear and well described study conduct and overall coherence. | Cross-sectional quantitative study with structured sampling, clear data sources, bivariate and regression analysis; results align with tables; reproducible. | Ethics approval reported | Design matches question; partial theory alignment; surveys and testing conducted. | Reluctance of patients to share partner information. Stigma associated with HIV/AIDS may hinder disclosure and implementations. | Authors' conclusions transparently detail findings, limitations, and implications. | Methods are relevant to study objectives and discussion well-grounded with the findings and relevant literature. | Results inform public health interventions. Shed light on partner notification in HIV care. |  |  |  |  |
|  | Reviewer's Score | 2 | 2 | 1 | 2 | 2 | 1 | 2 | 2 | 2 |  |  |  |  |
| Culbert GJ et al., 2023, Indonesia | **Rating** | **Yes**/No & Not sure/unclear | **Yes**/No & Not sure/unclear | **Yes**/No & Not sure/unclear | **Yes**/No & Not sure/unclear | **Yes**/No & Not sure/unclear | Yes/No & **Not sure/unclear** | **Yes**/No & Not sure/unclear | **Yes**/No & Not sure/unclear | **Yes**/No & Not sure/unclear | 17 | Low-risk | Included |  |
|  | Reviewer's response | Justification clear, exploring the acceptability of HIV PNS, results relevant to issues. | The study aim is clear and well described study conduct and overall coherence. | A cross-sectional quantitative study, structured sampling, criteria, clear information source, results aligned with tables, reproducible methods. | Ethics approval reported | Research question aligns with design, convenience sampling, surveys and testing conducted, Controls implicitly considered. | Not specifically described | Authors' conclusions transparently detail findings, limitations, and implications. | Methods are relevant to study objectives and discussion well-grounded with the findings and relevant literature. | Insights on prison HIV partner notification guide public health; address stigma, tailor interventions. |  |  |  |  |
|  | Reviewer's Score | 2 | 2 | 2 | 2 | 2 | 1 | 2 | 2 | 2 |  |  |  |  |
| Gitige CG et al., 2021, Tanzania | **Rating** | **Yes**/No & Not sure/unclear | **Yes**/No & Not sure/unclear | **Yes**/No & Not sure/unclear | **Yes**/No & Not sure/unclear | **Yes**/No & Not sure/unclear | Yes/No & **Not sure/unclear** | **Yes**/No & Not sure/unclear | **Yes**/No & Not sure/unclear | **Yes**/No & Not sure/unclear | 17 | Low-risk | Included |  |
|  | Reviewer's response | Justification clear, Factors associated with Partners Elicitation during HIV Index client´s testing, results consistent with issues. | The study aim is clear and well described study conduct and overall coherence. | A cross-sectional quantitative study, structured sampling, criteria, clear information source, results aligned with tables, reproducible methods. | Ethics approval reported | Research question aligns with design, stratified random sampling, surveys and testing conducted. | Sample size may limit generalizability. Possible recall bias in data collection. Statistical and Analytical Issues: Efforts to minimize interviewer bias through training. | Authors' conclusions transparently detail findings, limitations, and implications. | Methods are relevant to study objectives and discussion well-grounded with the findings and relevant literature. | Findings inform partner disclosure, shaping HIV testing, treatment, and prevention strategies |  |  |  |  |
|  | Reviewer's Score | 2 | 2 | 2 | 2 | 2 | 1 | 2 | 2 | 2 |  |  |  |  |
| Cibangu K, 2022, Zambia | **Rating** | **Yes**/No & Not sure/unclear | **Yes**/No & Not sure/unclear | **Yes**/No & Not sure/unclear | **Yes**/No & Not sure/unclear | **Yes**/No & Not sure/unclear | Yes/No & **Not sure/unclear** | **Yes**/No & Not sure/unclear | **Yes**/No & Not sure/unclear | **Yes**/No & Not sure/unclear | 17 | Low-risk | Included |  |
|  | Reviewer's response | Rationale clear, describing existing information on index testing and proposing strategies for effective index testing, results relevant to issues. | The study aim is clear and well described study conduct and overall coherence. | A cross-sectional quantitative study, structured sampling, criteria, clear information source, results aligned with tables, reproducible methods. | Ethics approval reported | Research question aligns with design, convenience sampling, PNS registers used for data and testing conducted. | Potential issues with programmatic data completeness and accuracy. Retrospective nature might introduce bias and limit capturing full dynamics. | Authors' conclusions transparently detail findings, limitations, and implications. | Methods are relevant to study objectives and discussion well-grounded with the findings and relevant literature. | Results inform HIV prevention strategies, notably in resource-limited settings like Zambia. |  |  |  |  |
|  | Reviewer's Score | 2 | 2 | 2 | 2 | 2 | 1 | 2 | 2 | 2 |  |  |  |  |
| Mwango LK et al., 2020, Zambia | **Rating** | **Yes**/No & Not sure/unclear | **Yes**/No & Not sure/unclear | **Yes**/No & Not sure/unclear | **Yes**/No & Not sure/unclear | **Yes**/No & Not sure/unclear | Yes/No & **Not sure/unclear** | **Yes**/No & Not sure/unclear | **Yes**/No & Not sure/unclear | **Yes**/No & Not sure/unclear | 16 | Low-risk | Included |  |
|  | Reviewer's response | Rationale clear, assessing the approaches to case finding and linkages to treatment through community-based index testing, results relevant to issues. | The study aim is clear and well described study conduct and overall coherence. | A cross-sectional quantitative study, structured sampling, criteria, clear information source, descriptive stats, Chi-square tests, results aligned with tables, partial reproducible method. | Ethics approval reported | Research question aligns with design, convenience sampling, PNS registers used for data and testing conducted. | Data Sources Bias: Limited to those accessing health services. Analysis Bias: Suitable statistical methods, assuming comprehensive factor consideration. | Authors' conclusions transparently detail findings, limitations, and implications. | Clear methods; consistent findings; limitations acknowledged; large sample with broad coverage. | Study's results on HIV testing and linkage guide public health. Engage hard-to-reach populations, improve HIV outcomes. |  |  |  |  |
|  | Reviewer's Score | 2 | 2 | 1 | 2 | 2 | 1 | 2 | 2 | 2 |  |  |  |  |
| Afe AJ et al., 2021, Nigeria | **Rating** | **Yes**/No & Not sure/unclear | **Yes**/No & Not sure/unclear | **Yes**/No & Not sure/unclear | **Yes**/No & Not sure/unclear | **Yes**/No & Not sure/unclear | Yes/No & **Not sure/unclear** | **Yes**/No & Not sure/unclear | **Yes**/No & Not sure/unclear | **Yes**/No & Not sure/unclear | 11.5 | Moderate Risk | Included |  |
|  | Reviewer's response | Justification clear, assessing the outcome of PNS, results consistent with study objectives. | The study aim is clear and well described study conduct and overall coherence. | Cross-sectional quantitative study with structured sampling, clear data sources, descriptive stats; results align with tables; partially reproducible | Ethics not reported, in the text it was mentioned informed consent obtained | Research question aligns with design, retrospective sampling, PNS registers used for data and testing conducted. | Methodology details missing. | Transparent conclusions with key finding and imitations. | Limited confidence; undisclosed flaws; results align with expectations but lack literature comparison | PNS findings inform public health; improve HIV testing and health outcomes |  |  |  |  |
|  | Reviewer's Score | 2 | 2 |  | 1 | 1 | 1 | 1.5 | 1 | 2 |  |  |  |  |
| Fu X et al., 2016, China | **Rating** | **Yes**/No & Not sure/unclear | **Yes**/No & Not sure/unclear | **Yes**/No & Not sure/unclear | **Yes**/No & Not sure/unclear | **Yes**/No & Not sure/unclear | Yes/No & **Not sure/unclear** | **Yes**/No & Not sure/unclear | **Yes**/No & Not sure/unclear | **Yes**/No & Not sure/unclear | 16 | Low-risk | Included |  |
|  | Reviewer's response | Rationale clear, exploring the feasibility and efficiency of sexual PN and HIV testing among HIV-positive MSM, results relevant to issues. | The study introduction, results, discussion, and overall coherence. | A cross-sectional quantitative study, structured sampling, criteria, clear information source, descriptive stats, results aligned with tables, partial reproducible method. | Ethics approval reported | Research question aligns with design, non-random and convenience sampling, PNS registers , face interview and questionaries and testing. | Does not directly identify sources of bias or mitigation measures but methods are clear. | Authors' conclusions transparently detail findings, limitations, and implications. | Short duration, small sample limit generalizability; limited comparisons; findings support PN and HIV testing for MSM | Results shape tailored interventions, strengthen partnerships. Expand partner notification for MSM, improve HIV testing, care. |  |  |  |  |
|  | Reviewer's Score | 2 | 2 | 2 | 2 | 2 | 1 | 2 | 1 | 2 |  |  |  |  |
| Ugbena ER et al., 2021, Nigeria | **Rating** | **Yes**/No & Not sure/unclear | **Yes**/No & Not sure/unclear | **Yes**/No & Not sure/unclear | **Yes**/No & Not sure/unclear | **Yes**/No & Not sure/unclear | Yes/No & **Not sure/unclear** | **Yes**/No & Not sure/unclear | **Yes**/No & Not sure/unclear | **Yes**/No & Not sure/unclear | 17 | Low-risk | Included |  |
|  | Reviewer's response | Justification clear, describing the level of acceptability and outcome of PNS among HIV-positive KPs, results consistent with study objectives. | The study introduction, results, discussion, and overall coherence. | A cross-sectional quantitative study, structured sampling, criteria, clear information source, descriptive stats, results aligned with tables, partial reproducible method. | Ethics approval reported | Research question aligns with design, non-random and convenience sampling, PNS registers, and testing. | Does not directly identify sources of bias or mitigation measures but methods are clear. | Authors' conclusions transparently detail findings, limitations, and implications. | No apparent flaws; findings align with HIV prevention trends in key populations. | Study's findings on partner notification among Key Populations inform targeted interventions, policies for HIV prevention, treatment. |  |  |  |  |
|  | Reviewer's Score | 2 | 2 | 2 | 2 | 2 | 1 | 2 | 2 | 2 |  |  |  |  |
| Emeh A et al., 2021, Nigeria | **Rating** | **Yes**/No & Not sure/unclear | **Yes**/No & Not sure/unclear | **Yes**/No & Not sure/unclear | **Yes**/No & Not sure/unclear | **Yes**/No & Not sure/unclear | Yes/**No** & Not **sure/unclear** | **Yes**/No & Not sure/unclear | **Yes**/No & Not sure/unclear | **Yes**/No & Not sure/unclear | 14 | Moderate Risk | Included |  |
|  | Reviewer's response | Justification clear, determining the positivity yield and identify factors influencing the yield from index testing strategy, results relevant to issues, results relevant to issues. | The study objective is clear, and all components described well. | Cross-sectional, quantitative study, structured sampling, criteria, clear information source, descriptive stats and chi-square test, results aligned with tables, partial reproducible method. | Unclear, but study conducted with APIN Public Health Initiative in Akure, Ondo State. | Research question aligns with design, non-random and convenience sampling, PNS registers, and testing. Methodology matched theoretical framework. | Text does not explicitly address the identification of bias sources and mitigation measures | Authors' conclusions transparently detail findings, limitations, and implications. | No major flaws; contributes to HIV testing literature; lacks direct comparison; cautious interpretation advised | Insights inform HIV prevention strategies, guide testing, linkage efforts. Direct implications for public health interventions, decision-making. |  |  |  |  |
|  | Reviewer's Score | 2 | 2 | 2 | 2 | 2 | 0 | 1 | 1 | 2 |  |  |  |  |
| Buhikire K et al., 2018, Uganda | **Rating** | **Yes**/No & Not sure/unclear | **Yes**/No & Not sure/unclear | **Yes**/No & Not sure/unclear | **Yes**/No & Not sure/unclear | **Yes**/No & Not sure/unclear | Yes/No & **Not sure/unclear** | **Yes**/No & Not sure/unclear | **Yes**/No & Not sure/unclear | **Yes**/No & Not sure/unclear | 17 | Low-risk | Included |  |
|  | Reviewer's response | Justification clear, pilot PNS describing predictors of successful contact tracings and testing of partners and barriers to contact, results relevant to issues. | The study objective is clear, and all components described well. | Cross-sectional quantitative study, structured sampling, criteria, clear information source, descriptive stats and regression, results aligned with tables, partial reproducible method. | Ethics approval reported | Research question aligns with design, non-random and convenience sampling, PNS registers, and testing. Methodology matched theoretical framework. | Acknowledges partner limit bias without mitigation details. Notes self-report bias, lacks verification strategies. no correction methods specified. lacks mitigation measures. | Authors' conclusions transparently detail findings, limitations, and implications. | Minor biases; no major flaws; results align with literature; high confidence due to clear reporting | The study's findings can guide public health in improving HIV prevention and partner notification strategies. |  |  |  |  |
|  | Reviewer's Score | 2 | 2 | 2 | 2 | 2 | 1 | 2 | 2 | 2 |  |  |  |  |
| Joel JN et al., 2022, Kenya | **Rating** | **Yes**/No & Not sure/unclear | **Yes**/No & Not sure/unclear | **Yes**/No & Not sure/unclear | **Yes**/No & Not sure/unclear | **Yes**/No & Not sure/unclear | Yes/No & **Not sure/unclear** | **Yes**/No & Not sure/unclear | **Yes**/No & Not sure/unclear | **Yes**/No & Not sure/unclear | 18 | Low-risk | Included |  |
|  | Reviewer's response | Justification clear, assessing outcomes along the HIV index testing cascade, results relevant to issues. | The study aim is clear and well described study conduct and overall coherence. | Cross-sectional quantitative study, structured sampling, criteria, clear information source, descriptive stats and chi-square test, results aligned with tables, partial reproducible method. | Ethics approval reported | Research question aligns with design, retrospective sampling, PNS registers, and testing. Methodology matched theoretical framework. | Bias Sources: Lack of comparison groups, aggregate data usage. Mitigation: Study's strength: inclusion of 48 facilities, diverse population coverage. Future research suggested for better understanding of key population testing preferences. | Transparent conclusions with key finding and imitations. | Methodology is robust. Results align with existing literature. | Index testing boosts testing rates, improves access; integration and monitoring optimize strategies |  |  |  |  |
|  | Reviewer's Score | 2 | 2 | 2 | 2 | 2 | 2 | 2 | 2 | 2 |  |  |  |  |
| Mahachi N et al., 2019, Zimbabwe | **Rating** | **Yes**/No & Not sure/unclear | **Yes**/No & Not sure/unclear | **Yes**/No & Not sure/unclear | **Yes**/No & Not sure/unclear | **Yes**/No & Not sure/unclear | Yes/No & **Not sure/unclear** | **Yes**/No & Not sure/unclear | **Yes**/No & Not sure/unclear | **Yes**/No & Not sure/unclear | 17 | Low-risk | Included |  |
|  | Reviewer's response | Rationale clear, describing the implementation of index testing and PNS under the Zimbabwe HIV care and treatment project, results consistent with objectives. | The study aim is clear and well described study conduct and overall coherence. | Methodology clearly described, including setting, target population, procedures, training, data sources, and analysis. Ethical approval noted. Results description limited in excerpt. | Ethics approval reported | Clear rationale and detailed methods per national guidelines; rigorous data collection and analysis; findings logically supported. | Partial identification of limitations and potential bias, but explicit bias identification and mitigation measures are limited or not fully detailed. | conclusions clearly state index testing and PNS effectively identify undiagnosed HIV cases and support epidemic control. | Findings are robust and reliable. | Yes, the results support effective HIV case finding and linkage strategies relevant to public health. |  |  |  |  |
|  | Reviewer's Score | 2 | 2 | 2 | 2 | 2 | 1 | 2 | 2 | 2 |  |  |  |  |
| Kariuki RM et al., 2020, Kenya. | **Rating** | **Yes**/No & Not sure/unclear | **Yes**/No & Not sure/unclear | **Yes**/No & Not sure/unclear | **Yes**/No & Not sure/unclear | **Yes**/No & Not sure/unclear | Yes/No & **Not sure/unclear** | **Yes**/No & Not sure/unclear | **Yes**/No & Not sure/unclear | **Yes**/No & Not sure/unclear | 13 | Moderate Risk | Included |  |
|  | Reviewer's response | Justification clear, enhancing PNS implementation for HIV case identification, results relevant to issues. | The study aim is clear and well described study conduct and overall coherence. | Cross-sectional quantitative study, structured sampling, criteria, clear information source, descriptive stats and chi-square test, results aligned with tables, partial reproducible method. | Informed consent obtained but ethics approval not reported | Research question aligns with design, facility-based sampling and convenience, PNS registers, and testing. | Incomplete data on sexual partners' marital status. Secondary data use may introduce biases. Lack of detail on follow-up procedures. No specified mitigation measures. | Transparent conclusions with key finding and imitations. | Limited confidence due to flaws; partial literature alignment; discrepancies need clearer explanation | Findings inform HIV prevention; secondary data limits; local adaptation needed; further research recommended |  |  |  |  |
|  | Reviewer's Score | 2 | 2 | 2 | 1 | 2 | 1 | 1 | 0 | 2 |  |  |  |  |
| Remera E et al., 2022, Rwanda | **Rating** | **Yes**/No & Not sure/unclear | **Yes**/No & Not sure/unclear | **Yes**/No & Not sure/unclear | **Yes**/No & Not sure/unclear | **Yes**/No & Not sure/unclear | Yes/No & **Not sure/unclear** | **Yes**/No & Not sure/unclear | **Yes**/No & Not sure/unclear | **Yes**/No & Not sure/unclear | 16.5 | Low-risk | Included |  |
|  | Reviewer's response | Justification clear, assessing the effectiveness of voluntary aPNS modalities, results relevant to issues. | The study aim is clear and well described study conduct and overall coherence. | Observational cohort study with structured sampling, clear data sources, descriptive stats and regression; results align with tables; partially reproducible | Ethics approval reported | Research question aligns with design, purposive and convenience sampling, surveys and testing conducted. | The text does not explicitly identify sources of bias or mitigation measures, but the statistical methods used likely account for potential biases through adjustment for confounding factors. | Clear conclusions, detailed discussion, positive outcomes, stated primary and secondary outcomes. | No major flaws; consistency with literature unclear due to limited access | Relevant to HIV prevention; informs testing strategies; supports improved testing and care linkage. |  |  |  |  |
|  | Reviewer's Score | 2 | 2 | 2 | 2 | 2 | 1 | 2 | 1.5 | 2 |  |  |  |  |
| Golden MR et al., 2023, Mozambique | **Rating** | **Yes**/No & Not sure/unclear | **Yes**/No & Not sure/unclear | **Yes**/No & Not sure/unclear | **Yes**/No & Not sure/unclear | **Yes**/No & Not sure/unclear | Yes/No & **Not sure/unclear** | **Yes**/No & Not sure/unclear | **Yes**/No & Not sure/unclear | **Yes**/No & Not sure/unclear | 17 | Low-risk | Included |  |
|  | Reviewer's response | Justification clear, assessing the effectiveness and safety of APS in a large APS program, results relevant to issues. | The study aim is clear and well described study conduct and overall coherence. | Observational cohort study with structured sampling, clear data sources, descriptive stats and regression; results align with tables; partially reproducible. | Ethics approval reported | Research question aligns with design, purposive and convenience sampling, surveys and testing conducted. | Identification of bias and mitigation measures are not provided | Clear conclusions, detailed discussion, positive outcomes, stated primary and secondary outcomes. | No major flaws; detailed methods support credibility; bias mitigation not discussed. | Findings inform public health, but caution due to context and limited population inclusion |  |  |  |  |
|  | Reviewer's Score | 2 | 2 | 2 | 2 | 2 | 1 | 2 | 2 | 2 |  |  |  |  |
| Opeyemi A et al., 2021, Nigeria | **Rating** | **Yes**/No & Not sure/unclear | **Yes**/No & Not sure/unclear | **Yes**/No & Not sure/unclear | **Yes**/No & Not sure/unclear | **Yes**/No & Not sure/unclear | Yes/No & **Not sure/unclear** | **Yes**/No & Not sure/unclear | **Yes**/No & Not sure/unclear | **Yes**/No & Not sure/unclear | 17 | Low-risk | Included |  |
|  | Reviewer's response | Justification clear, evaluating the case-finding effectiveness of HIV PNS, results relevant to issues. | The study aim is clear and well described study conduct and overall coherence. | Observational cohort, quantitative study, structured sampling, criteria, clear information source, descriptive stats, results aligned with tables, partial reproducible method. | Ethics approval reported | Research question aligns with design, purposive and convenience sampling, surveys and testing conducted. | Limitations are highlighted but no source of information for bias and measures highlighted | Clear conclusions, detailed discussion, positive outcomes, stated primary and secondary outcomes. | No major methodological flaws identified. Detailed methodology supports credibility. Explicit bias mitigation discussion lacking. | Findings support PNS for HIV case identification and care linkage in resource-limited settings like Nigeria |  |  |  |  |
|  | Reviewer's Score | 2 | 2 | 2 | 2 | 2 | 1 | 2 | 2 | 2 |  |  |  |  |
| Katbi M et al., 2018, Nigeria | **Rating** | **Yes**/No & Not sure/unclear | **Yes**/No & Not sure/unclear | **Yes**/No & Not sure/unclear | **Yes**/No & Not sure/unclear | **Yes**/No & Not sure/unclear | Yes/No & **Not sure/unclear** | **Yes**/No & Not sure/unclear | **Yes**/No & Not sure/unclear | **Yes**/No & Not sure/unclear | 18 | Low-risk | Included |  |
|  | Reviewer's response | Justification clear, evaluating the impact of index testing through PNS, results relevant to issues. | Clear introduction, methods, results, discussion, organization, language, figures/tables, citations, conclusion, and overall coherence. | Quasi-experimental study, structured sampling, criteria, clear information source, descriptive stats and chi-square test, results aligned with tables, partial reproducible method. | Ethics approval reported | Research question aligns with design, purposive and convenience sampling, PNS registers and testing conducted. | Bias from self-reporting, Mitigation: Anonymous data. Testing barriers bias outcomes, Mitigation: Targeted interventions. | Transparent conclusions with key finding and imitations. | No major flaws; findings valid; consistent with literature on HIV prevalence among PLHIV partner | Results inform targeted HIV prevention efforts. Guides strategies to reduce transmission. Enhances public health interventions. |  |  |  |  |
|  | Reviewer's Score | 2 | 2 | 2 | 2 | 2 | 2 | 2 | 2 | 2 |  |  |  |  |
| Sharma M et al., 2021, Kenya | **Rating** | **Yes**/No & Not sure/unclear | **Yes**/No & Not sure/unclear | **Yes**/No & Not sure/unclear | **Yes**/No & Not sure/unclear | **Yes**/No & Not sure/unclear | Yes/No & **Not sure/unclear** | **Yes**/No & Not sure/unclear | **Yes**/No & Not sure/unclear | **Yes**/No & Not sure/unclear | 17 | Low-risk | Included |  |
|  | Reviewer's response | Justification clear, assessing the acceptability of PNS among HIV positive females, results relevant to issues. | Clear introduction, methods, results, discussion, organization, language, figures/tables, citations, conclusion, and overall coherence. | Prospective cohort study, structured sampling, criteria, clear information source, Multivariate regression, results aligned with tables, partial reproducible method. | Ethics approval not mentioned but informed consent obtained | Research question aligns with design, purposive sampling, survey and testing conducted. | Some limitations are highlighted but not detailed source of bias and mitigation measures. | Transparent conclusions with key finding and imitations. | No major methodological flaws are evident. Results align with existing literature on HIV testing. Any differences in results are explained | The findings inform policy and interventions, optimize resource allocation, and strengthen healthcare systems for HIV prevention and care. |  |  |  |  |
|  | Reviewer's Score | 2 | 2 | 2 | 2 | 2 | 1 | 2 | 2 | 2 |  |  |  |  |
| Kiene SM et al., 2017, Uganda | **Rating** | **Yes**/No & Not sure/unclear | **Yes**/No & Not sure/unclear | **Yes**/No & Not sure/unclear | **Yes**/No & Not sure/unclear | **Yes**/No & Not sure/unclear | Yes/No & **Not sure/unclear** | **Yes**/No & Not sure/unclear | **Yes**/No & Not sure/unclear | **Yes**/No & Not sure/unclear | 17 | Low-risk | Included |  |
|  | Reviewer's response | Justification clear, assessing the perceived feasibility and acceptability of index partner using HIVST, results relevant to issues. | Clear introduction, methods, results, discussion, organization, language, figures/tables, citations, conclusion, and overall coherence. | Prospective cohort study, structured sampling, criteria, clear information source, descriptive and logistic regression, results aligned with tables, partial reproducible method. | Ethics approval reported | Research question aligns with design, non-random convenience sampling, interview surveys and HIV testing conducted. | Non-random sampling, limited eligibility. Potential underpowered sample size. Reliance on self-reporting, potential bias. Lack of partner HIV testing report verification. | conclusions are clear and transparent, derived directly from the results with acknowledgment of potential discrepancies and limitations. | No major methodological flaws identified. Detailed methodology supports credibility. Explicit bias mitigation discussion lacking. | The results can be applied within the scope of public health. |  |  |  |  |
|  | Reviewer's Score | 2 | 2 | 2 | 2 | 2 | 1 | 2 | 2 | 2 |  |  |  |  |
| Semple SJ et al., 2018, Mexico | **Rating** | **Yes**/No & Not sure/unclear | **Yes**/No & Not sure/unclear | **Yes**/No & Not sure/unclear | **Yes**/No & Not sure/unclear | **Yes**/No & Not sure/unclear | Yes/No & **Not sure/unclear** | **Yes**/No & Not sure/unclear | **Yes**/No & Not sure/unclear | **Yes**/No & Not sure/unclear | 17 | Low-risk | Included |  |
|  | Reviewer's response | Justification clear, assessing the uptake and outcomes of a partner notification among MSM and TG, Results relevant to issues. | The study introduction is clear, and all components described well. | Observational cohort study, structured sampling, criteria, clear information source, descriptive and chi-square, results aligned with tables, partial reproducible method. | Ethics approval reported | Research question aligns with design, non-random convenience and purposeful sampling, interview surveys and HIV testing conducted. Analysis accounts for important theoretical factors. | Social desirability bias Misclassification of outcomes | Transparent conclusions with key finding and imitations. | No major methodological flaws identified. Detailed methodology supports credibility. Explicit bias mitigation discussion lacking. | The results can be applied within the scope of public health. |  |  |  |  |
|  | Reviewer's Score | 2 | 2 | 2 | 2 | 2 | 1 | 2 | 2 | 2 |  |  |  |  |
| Offorjebe OA et al., 2020, Malawi | **Rating** | **Yes**/No & Not sure/unclear | **Yes**/No & Not sure/unclear | **Yes**/No & Not sure/unclear | **Yes**/No & Not sure/unclear | **Yes**/No & Not sure/unclear | Yes/No & **Not sure/unclear** | **Yes**/No & Not sure/unclear | **Yes**/No & Not sure/unclear | **Yes**/No & Not sure/unclear | 17 | Low-risk | Included |  |
|  | Reviewer's response | Rationale clear, assessing the acceptability of index partner HIV self-testing, results relevant to issues. | The study aim is clear and well described study conduct and overall coherence. | Cross-sectional study, structured sampling, criteria, clear information source, descriptive, results aligned with tables, partial reproducible method. | Ethics approval reported | Design fits question; cluster and purposive sampling; interviews and HIV testing; methods align with framework | Identified biases with proposed solutions: robust design, clear criteria, reliable data, rigorous analysis, diverse inclusion, transparent reporting, and sensitivity analyses. | Transparent conclusions with key finding and imitations. | Moderate confidence; no major flaws; compares with literature; hypothetical scenario limits confidence. | Results applicable to public health, especially for enhancing HIV testing among male partners. |  |  |  |  |
|  | Reviewer's Score | 2 | 2 | 2 | 2 | 2 | 2 | 2 | 1 | 2 |  |  |  |  |
| Edosa M et al., 2022, Ethiopia | **Rating** | **Yes**/No & Not sure/unclear | **Yes**/No & Not sure/unclear | **Yes**/No & Not sure/unclear | **Yes**/No & Not sure/unclear | **Yes**/No & Not sure/unclear | **Yes**/No & Not sure/unclear | **Yes**/No & Not sure/unclear | **Yes**/No & Not sure/unclear | **Yes**/No & Not sure/unclear | 18 | Low-risk | Included |  |
|  | Reviewer's response | Justification clear, assessing magnitude and factors associated with PNS among HIV cases, results relevant to issues. | The study aim is clear and well described study conduct and overall coherence. | Cross-sectional study, structured sampling, criteria, clear information source, descriptive and logistic regression, results aligned with tables, partial reproducible method. | Ethics approval reported | Research question aligns with design, systematic random sampling, survey and secondary data sued and testing. | Identify biases like social desirability and recall, mitigated by training data collectors and pre-testing the questionnaire. | Transparent conclusions with key finding and imitations. | The study uses appropriate methodology, addresses potential biases, and provides clear results, increasing confidence in the reliability of the finding | The results are applicable to public health, informing strategies for HIV testing and partner notification services. |  |  |  |  |
|  | Reviewer's Score | 2 | 2 | 2 | 2 | 2 | 2 | 2 | 2 | 2 |  |  |  |  |
| Grande M et al., 2021, Botswana | **Rating** | **Yes**/No & Not sure/unclear | **Yes**/No & Not sure/unclear | **Yes**/No & Not sure/unclear | **Yes**/No & Not sure/unclear | **Yes**/No & Not sure/unclear | **Yes**/No & Not sure/unclear | **Yes**/No & Not sure/unclear | **Yes**/No & Not sure/unclear | **Yes**/No & Not sure/unclear | 17 | Low-risk | Included |  |
|  | Reviewer's response | Justification clear, Evaluating Botswana's aPNS for reach, and effectiveness, results relevant to issues. | The study aim is clear and well described study conduct and overall coherence. | Observational cohort study, structured sampling, criteria, clear information source, descriptive and proportion, results aligned with tables, partial reproducible method. | Ethics approval reported | Research question aligns with design, convenience sampling, structured interview records, electronic databases, paper registries, and testing. | Biases: Selection, recall, publication. Mitigation: Adjusting for confounders, standardizing data collection, sensitivity analyses. | Transparent conclusions with key finding and imitations. | The findings given the clear presentation of methodology, results, identification of biases, and transparent conclusions provided by the authors. | Results applicable to public health due to the study's relevance, clear methodology, and potential implications for practice or policy. |  |  |  |  |
|  | Reviewer's Score | 2 | 2 | 2 | 2 | 2 | 1 | 2 | 2 | 2 |  |  |  |  |
| Selvaraj K et al., 2017, India | **Rating** | **Yes**/No & Not sure/unclear | **Yes**/No & Not sure/unclear | **Yes**/No & Not sure/unclear | **Yes**/No & Not sure/unclear | **Yes**/No & Not sure/unclear | Yes/No & **Not sure/unclear** | **Yes**/No & Not sure/unclear | **Yes**/No & Not sure/unclear | **Yes**/No & Not sure/unclear | 16 | Low-risk | Included |  |
|  | Reviewer's response | Justification clear, Assessing PLHIV with untested partners, analyze profiles, understand healthcare provider perspectives, suggest testing improvements. | The study introduction, results, discussion, and overall coherence. | Exploratory mixed method study, structured sampling, criteria, clear information source, descriptive and proportion, results aligned with tables, partial reproducible method. | Ethics approval reported | Research question aligns with design, convenience sampling, electronic data-base and testing. | The study notes data quality and missing data issues but doesn't specify bias sources or mitigation. Thus, no discussion on bias and mitigation is included. | Transparent conclusions with key finding and imitations. | Findings reliable despite limitations; no major flaws; valuable insights into partner testing in Gujarat | Findings inform public health strategies in Gujarat by addressing partner testing barriers and improving care |  |  |  |  |
|  | Reviewer's Score | 2 | 2 | 1 | 2 | 2 | 1 | 2 | 2 | 2 |  |  |  |  |
| Rahmalia A et al., 2022, Indonesia | **Rating** | **Yes**/No & Not sure/unclear | **Yes**/No & Not sure/unclear | **Yes**/No & Not sure/unclear | **Yes**/No & Not sure/unclear | **Yes**/No & Not sure/unclear | Yes/**No** & Not sure/unclear | **Yes**/No & Not sure/unclear | **Yes**/No & Not sure/unclear | **Yes**/No & Not sure/unclear | 16 | Low-risk | Included |  |
|  | Reviewer's response | Justification clear, assessing facilitators and barriers to status disclosure and partner testing of HIV positive women, results consistent with issues. | The study aim is clear and well described study conduct and overall coherence. | Exploratory mixed method study, structured sampling, criteria, clear information source, descriptive and proportion, results aligned with tables, partial reproducible method. | Ethics approval reported | Research question aligns with design, convenience sampling, survey and testing conducted. | The text doesn't address bias sources or mitigation measures. It mainly discusses study design and data collection challenges. | Transparent conclusions with key finding and imitations. | No major methodological flaws, and the results are consistent with existing literature on the topic. Therefore, the findings are reliable. | Findings inform HIV prevention and care interventions for women to reduce transmission. |  |  |  |  |
|  | Reviewer's Score | 2 | 2 | 2 | 2 | 2 | 0 | 2 | 2 | 2 |  |  |  |  |
| Wamuti B et al., 2023, Kenya | **Rating** | **Yes**/No & Not sure/unclear | **Yes**/No & Not sure/unclear | **Yes**/No & Not sure/unclear | **Yes**/No & Not sure/unclear | **Yes**/No & Not sure/unclear | **Yes**/No & Not sure/unclear | **Yes**/No & Not sure/unclear | **Yes**/No & Not sure/unclear | **Yes**/No & Not sure/unclear | 18 | Low-risk | Included |  |
|  | Reviewer's response | Justification clear, assessing factors affecting implementation fidelity to aPNS, results are relevant to issues. | The study aim is clear and well described study conduct and overall coherence. | Exploratory mixed method study, structured sampling, criteria, clear information source, descriptive and proportion, results aligned with tables, partial reproducible method. | Ethics approval reported | Research question aligns with design, convenience sampling, survey and in-depth-interviews. | The text doesn't address bias sources or mitigation measures. It mainly discusses study design and data collection challenges. | Clear conclusions, detailed discussion, positive outcomes, stated primary and secondary outcomes. | Mixed methods strengthen findings; flaws addressed; differences explained; results align with objectives | Confidence in findings justified by robust methods, addressed flaws, transparent explanation of differences, and alignment with objectives. |  |  |  |  |
|  | Reviewer's Score | 2 | 2 | 2 | 2 | 2 | 2 | 2 | 2 | 2 |  |  |  |  |
| Vermandere H et al., 2021, Mexico | **Rating** | **Yes**/No & Not sure/unclear | **Yes**/No & Not sure/unclear | **Yes**/No & Not sure/unclear | **Yes**/No & Not sure/unclear | **Yes**/No & Not sure/unclear | Yes/No & **Not sure/unclear** | **Yes**/No & Not sure/unclear | **Yes**/No & Not sure/unclear | **Yes**/No & Not sure/unclear | 17 | Low-risk | Included |  |
|  | Reviewer's response | Rationale clear, exploring the awareness of and need for HIV PNS, results are relevant to issues. | The study aim is clear and well described study conduct and overall coherence. | Exploratory mixed method study, structured sampling, criteria, clear information source, descriptive and proportion, results aligned with tables, partial reproducible method. | Ethics approval reported | Research question aligns with design, purposeful sampling, semi-structured interviews. | The study acknowledges potential biases through its limitations but does not detail specific mitigation measures. | Clear conclusions, detailed discussion, positive outcomes, stated primary and secondary outcomes. | Minor methodological limitations; broadly consistent with existing literature; findings reasonably reliable within context | Within public health scope, especially in HIV prevention and care strategies, acknowledging context-specific adaptations. |  |  |  |  |
|  | Reviewer's Score | 2 | 2 | 2 | 2 | 2 | 1 | 2 | 2 | 2 |  |  |  |  |
| Yan XM et al., 2022, China | **Rating** | **Yes**/No & Not sure/unclear | **Yes**/No & Not sure/unclear | **Yes**/No & Not sure/unclear | **Yes**/No & Not sure/unclear | **Yes**/No & Not sure/unclear | Yes/No & **Not sure/unclear** | **Yes**/No & Not sure/unclear | **Yes**/No & Not sure/unclear | **Yes**/No & Not sure/unclear | 17 | Low-risk | Included |  |
|  | Reviewer's response | Justification clear, assessing facilitators and barriers of HIV PNS among MSM, results consistent with study objectives. | Clear introduction, methods, results, discussion, organization, language, figures/tables, citations, conclusion, and overall coherence. | Exploratory mixed method study, structured sampling, criteria, clear information source, descriptive and proportion, results aligned with tables, partial reproducible method. | Ethics approval reported | Research question aligns with design, convenience sampling, data collected- crowdsourcing platform. | Limitations were reported but they do not explicitly identify sources of bias or mitigation measures. | Clear conclusions, detailed discussion, positive outcomes, stated primary and secondary outcomes. | Trustworthy findings; sound methods; consistent with MSM HIV partner services literature; limitations discussed. | The results can be applied within the scope of public health, particularly in addressing HIV partner services and stigma associated with HIV. |  |  |  |  |
|  | Reviewer's Score | 2 | 2 | 2 | 2 | 2 | 1 | 2 | 2 | 2 |  |  |  |  |
| Quinn C et al., 2018, Uganda | **Rating** | **Yes**/No & Not sure/unclear | **Yes**/No & Not sure/unclear | **Yes**/No & Not sure/unclear | **Yes**/No & Not sure/unclear | **Yes**/No & Not sure/unclear | Yes/No & **Not sure/unclear** | **Yes**/No & Not sure/unclear | **Yes**/No & Not sure/unclear | **Yes**/No & Not sure/unclear | 17 | Low-risk | Included |  |
|  | Reviewer's response | Justification clear, assessing community reactions to both passive and assisted partner notification approaches, results relevant to issues. | Clear intro, methods, results, discussion, organization, language, visuals, citations, and coherence | Exploratory mixed method study, structured sampling, criteria, clear information source, descriptive and proportion, results aligned with tables, partial reproducible method. | Ethics approval reported | Research question aligns with design, purposeful sampling, IDIs and FGDs. | Limitations are highlighted but it does not explicitly identify sources of bias or mitigation measures. | The authors’ conclusions are explicit and transparent. | No major methodological flaws, and the results are consistent with existing literature on the topic. Therefore, the findings are reliable. | Findings inform HIV partner notification programs, addressing identified barriers and facilitators |  |  |  |  |
|  | Reviewer's Score | 2 | 2 | 2 | 2 | 2 | 1 | 2 | 2 | 2 |  |  |  |  |
| Sanga E et al., 2023, Tanzania | **Rating** | **Yes**/No & Not sure/unclear | **Yes**/No & Not sure/unclear | **Yes**/No & Not sure/unclear | **Yes**/No & Not sure/unclear | **Yes**/No & Not sure/unclear | Yes/No & **Not sure/unclear** | **Yes**/No & Not sure/unclear | **Yes**/No & Not sure/unclear | **Yes**/No & Not sure/unclear | 17 | Low-risk | Included |  |
|  | Reviewer's response | Justification clear, exploring decision-making around disclosure to sexual partners among PLHIV, results relevant to issues. | Clear structure, methods, results, discussion, visuals, citations, and overall coherence. | Exploratory mixed method study, structured sampling, criteria, clear information source, descriptive and proportion, results aligned with tables, partial reproducible method. | Ethics approval reported | Research question aligns with design, purposeful sampling, IDIs and FGDs. | Limitations are mentioned but it does not contain the source of bias and mitigation measure. | the authors’ conclusions are explicit and transparent. | No major methodological flaws, and the results are consistent with existing literature on the topic. Therefore, the findings are reliable. | Findings inform HIV disclosure, care engagement, and prevention strategies for PLHIV on ART. |  |  |  |  |
|  | Reviewer's Score | 2 | 2 | 2 | 2 | 2 | 1 | 2 | 2 | 2 |  |  |  |  |
| Liu W et al., 2022, Kenya | **Rating** | **Yes**/No & Not sure/unclear | **Yes**/No & Not sure/unclear | **Yes**/No & Not sure/unclear | **Yes**/No & Not sure/unclear | **Yes**/No & Not sure/unclear | Yes/No & **Not sure/unclear** | **Yes**/No & Not sure/unclear | **Yes**/No & Not sure/unclear | **Yes**/No & Not sure/unclear | 14 | Moderate Risk | Included |  |
|  | Reviewer's response | Justification clear, evaluating provider acceptability of HIV aPNS, results consistent with study objectives. | The study aim is clear and well described study conduct and overall coherence. | Exploratory mixed method study, structured sampling, criteria, clear information source, descriptive and proportion, results aligned with tables, partial reproducible method. | Ethics approval reported | Research question aligns with design, purposive sampling, IDIs and FGDs. | The text lacks explicit mention of bias sources or mitigation measures. It highlights data collection challenges and a small sample size as limitations. | Clear conclusions, detailed discussion, positive outcomes, stated primary and secondary outcomes. | No major methodological flaws, and the results are consistent with existing literature on the topic. Therefore, the findings are reliable. | Public health implications are substantial, particularly for enhancing HIV testing and care linkage strategies in similar settings. |  |  |  |  |
|  | Reviewer's Score | 2 | 1 | 1 | 2 | 2 | 1 | 2 | 1 | 2 |  |  |  |  |
| Aliza MW et al., 2019, Kenya | **Rating** | **Yes**/No & Not sure/unclear | **Yes**/No & Not sure/unclear | **Yes**/No & Not sure/unclear | **Yes**/No & Not sure/unclear | **Yes**/No & Not sure/unclear | Yes/No & **Not sure/unclear** | **Yes**/No & Not sure/unclear | **Yes**/No & Not sure/unclear | **Yes**/No & Not sure/unclear | 17 | Low-risk | Included |  |
|  | Reviewer's response | Justification clear, assessing barriers and opportunities for scaling up PNS, results relevant to issues. | The study aim is clear and well described study conduct and overall coherence. | Exploratory mixed method study, structured sampling, criteria, clear information source, descriptive and proportion, results aligned with tables, partial reproducible method. | Ethics approval reported | Research question aligns with design, non-random purposive sampling, IDIs and FGDs. | Some limitations are highlighted but not detailed source of bias and mitigation measures. | Clear conclusions, detailed discussion, positive outcomes, stated primary and secondary outcomes. | No major methodological flaws, and the results are consistent with existing literature on the topic. Therefore, the findings are reliable. | The study's findings are likely applicable in public health, particularly for informing HIV testing and partner notification strategies. |  |  |  |  |
|  | Reviewer's Score | 2 | 2 | 2 | 2 | 2 | 1 | 2 | 2 | 2 |  |  |  |  |
| Zhang K et al. | **Rating** | **Yes**/No & Not sure/unclear | **Yes**/No & Not sure/unclear | **Yes**/No & Not sure/unclear | **Yes**/No & Not sure/unclear | **Yes**/No & Not sure/unclear | Yes/No & **Not sure/unclear** | **Yes**/No & Not sure/unclear | **Yes**/No & Not sure/unclear | **Yes**/No & Not sure/unclear | 17 | Low-risk | Included |  |
|  | Reviewer's response | Justification clear, assessing perceived facilitators and barriers regarding PNS among PLHIV, results relevant to issues. | The study aim is clear and well described study conduct and overall coherence. | Exploratory mixed method study, structured sampling, criteria, clear information source, descriptive and proportion, results aligned with tables, partial reproducible method. | Ethics approval reported | Research question aligns with design, non-random purposive sampling, IDIs and FGDs. | Limitations mention data collection constraints and cultural factors, but don't address bias sources or mitigation. | Clear conclusions, detailed discussion, positive outcomes, stated primary and secondary outcomes. | No major flaws; consistent with literature; findings reliable | Findings inform partner notification interventions to support HIV prevention. |  |  |  |  |
|  | Reviewer's Score | 2 | 2 | 2 | 2 | 2 | 1 | 2 | 2 | 2 |  |  |  |  |
| Wirawan GB et al., 2021, Indonesia | **Rating** | **Yes**/No & Not sure/unclear | **Yes**/No & Not sure/unclear | **Yes**/No & Not sure/unclear | **Yes**/No & Not sure/unclear | **Yes**/No & Not sure/unclear | Yes/No & **Not sure/unclear** | **Yes**/No & Not sure/unclear | **Yes**/No & Not sure/unclear | **Yes**/No & Not sure/unclear | 18 | Low-risk | Included | Seti |
|  | Reviewer's response | Focus on HIV partner notification in Indonesia | Clear background, aims, methods, and findings | Methods and results well described with quotes | Ethics approval and consent described | Qualitative design fits research questions | Triangulation and inductive coding reduce bias | Conclusions clearly linked to data and limitations | Saturation and triangulation support confidence | Directly informs tailored aPN implementation |  |  |  |  |
|  | Reviewer's Score | 2 | 2 | 2 | 2 | 2 | 2 | 2 | 2 | 2 |  |  |  |  |
| Goyette M et al., 2016, Kenya | **Rating** | **Yes**/No & Not sure/unclear | **Yes**/No & Not sure/unclear | **Yes**/No & Not sure/unclear | **Yes**/No & Not sure/unclear | **Yes**/No & Not sure/unclear | Yes/No & **Not sure/unclear** | **Yes**/No & Not sure/unclear | **Yes**/No & Not sure/unclear | **Yes**/No & Not sure/unclear | 17 | Low-risk | Included |  |
|  | Reviewer's response | Justification clear, assessing barriers to scaling up HIV Assisted Partner Services in Kenya, results relevant to issues. | The study aim is clear and well described study conduct and overall coherence. | Exploratory mixed-method study with structured sampling, clear data sources, descriptive stats; results align with tables; partially reproducible | Ethics approval reported | Research question aligns with design, purposive and quota sampling, IDIs and FGDs. | Limitations are mentioned but it does not contain the source of bias and mitigation measure. | Clear conclusions, detailed discussion, positive outcomes, stated primary and secondary outcomes. | No major methodological flaws, and the results are consistent with existing literature on the topic. Therefore, the findings are reliable. | Small sample and non-random sampling, but findings provide useful public health insights. |  |  |  |  |
|  | Reviewer's Score | 2 | 2 | 2 | 2 | 2 | 1 | 2 | 2 | 2 |  |  |  |  |
| Benemariya N et al., 2023, Rwanda | **Rating** | **Yes**/No & Not sure/unclear | **Yes**/No & Not sure/unclear | **Yes**/No & Not sure/unclear | **Yes**/No & Not sure/unclear | **Yes**/No & Not sure/unclear | Yes/No & **Not sure/unclear** | **Yes**/No & Not sure/unclear | **Yes**/No & Not sure/unclear | **Yes**/No & Not sure/unclear | 17 | Low-risk | Included |  |
|  | Reviewer's response | Justification clear, assessing factors associated with PNS among PLHIV, results consistent with issues. | The study aim is clear and well described study conduct and overall coherence. | Case-control study with structured sampling, clear data sources, logistic regression; results align with tables; partially reproducible | Ethics approval reported | Research question aligns with design, consecutive sampling, surveys and testing done. | Limitations are mentioned but it does not contain the source of bias and mitigation measure. | The authors’ conclusions are explicit and transparent. | No major flaws; findings consistent with literature; results reliable | Small sample and non-random sampling, but findings offer valuable public health insights. |  |  |  |  |
|  | Reviewer's Score | 2 | 2 | 2 | 2 | 2 | 1 | 2 | 2 | 2 |  |  |  |  |
| Sharma M et al., 2018, Kenya | **Rating** | **Yes**/No & Not sure/unclear | **Yes**/No & Not sure/unclear | **Yes**/No & Not sure/unclear | **Yes**/No & Not sure/unclear | **Yes**/No & Not sure/unclear | Yes/No & **Not sure/unclear** | **Yes**/No & Not sure/unclear | **Yes**/No & Not sure/unclear | **Yes**/No & Not sure/unclear | 18 | Low-risk | Included |  |
|  | Reviewer's response | Justification clear, evaluating aPNS effectiveness and cost-effectiveness analysis, results consistent with study objectives. | The study aim is clear and well described study conduct and overall coherence. | Cluster RCT with modeling, structured sampling, clear data sources, sensitivity analysis; results align with tables; partially reproducible | Ethics approval reported | Design fits questions; cluster sampling; micro-costing study; uses KAIS dataset and literature | Identifies biases (selection, self-reporting) and suggests mitigation (randomization, validated surveys) with sensitivity analyses for uncertainties. | The authors’ conclusions are explicit and transparent. | you can be confident in the findings as there are no major methodological flaws, and results align with existing literature. | The results offer valuable insights applicable to public health, informing policies and programs for HIV prevention and treatment interventions. |  |  |  |  |
|  | Reviewer's Score | 2 | 2 | 2 | 2 | 2 | 2 | 2 | 2 | 2 |  |  |  |  |
| Wamuti B et al., 2022, Kenya | **Rating** | **Yes**/No & Not sure/unclear | **Yes**/No & Not sure/unclear | **Yes**/No & Not sure/unclear | **Yes**/No & Not sure/unclear | **Yes**/No & Not sure/unclear | Yes/No & **Not sure/unclear** | **Yes**/No & Not sure/unclear | **Yes**/No & Not sure/unclear | **Yes**/No & Not sure/unclear | 17 | Low-risk | Included |  |
|  | Reviewer's response | Justification clear, assessing the cost for integrating PNS in HIV services, results relevant to issues. | The study introduction, results, discussion, and overall coherence. | Micro-costing study with structured sampling, clear data sources, descriptive stats; results align with tables; partially reproducible | Ethics approval reported | Research question aligns with design, purposive sampling, Micro-costing method, semi-structured interviews, and HTS facility registers. | No sources of bias and mitigation measures in relation to the reported limitations. | The authors’ conclusions are explicit and transparent. | Findings reliable; no major flaws; consistent with literature; discrepancies explained | Findings relevant for public health, especially for integrating assisted partner services in resource-limited settings |  |  |  |  |
|  | Reviewer's Score | 2 | 2 | 2 | 2 | 2 | 1 | 2 | 2 | 2 |  |  |  |  |
| Songane M et al., 2023, Mozambique | **Rating** | **Yes**/No & Not sure/unclear | **Yes**/No & Not sure/unclear | **Yes**/No & Not sure/unclear | **Yes**/No & Not sure/unclear | **Yes**/No & Not sure/unclear | Yes/No & **Not sure/unclear** | **Yes**/No & Not sure/unclear | **Yes**/No & Not sure/unclear | **Yes**/No & Not sure/unclear | 17 | Low-risk | Included |  |
|  | Reviewer's response | Justification clear, estimating the cost-efficiency and effectiveness of community index testing  and compare the HIV testing outputs with facility-based testing. | The study introduction, results, discussion, and overall coherence. | Cost analysis with structured sampling, clear data sources, descriptive stats; results align with tables; partially reproducible | Ethics approval reported | Research question aligns with design, purposive sampling, MRS database, Routine program data. | No sources of bias and mitigation measures in relation to the reported limitations. | The authors’ conclusions are explicit and transparent. | Addresses methods and literature gaps; provides insights into the cost-effectiveness of community index testing. | The results have implications for public health planning and resource allocation in HIV testing programs. |  |  |  |  |
|  | Reviewer's Score | 2 | 2 | 2 | 2 | 2 | 1 | 2 | 2 | 2 |  |  |  |  |
| Cherutich P et al., 2018, Kenya | **Rating** | **Yes**/No & Not sure/unclear | **Yes**/No & Not sure/unclear | **Yes**/No & Not sure/unclear | **Yes**/No & Not sure/unclear | **Yes**/No & Not sure/unclear | Yes/No & **Not sure/unclear** | **Yes**/No & Not sure/unclear | **Yes**/No & Not sure/unclear | **Yes**/No & Not sure/unclear | 17 | Low-risk | Included |  |
|  | Reviewer's response | Justification clear, a cost and budget impact analysis of PNS, results relevant to issues. | The study introduction, results, discussion, and overall coherence. | Budget impact analysis with structured sampling, clear data sources, cost analysis; results align with tables; partially reproducible. | Ethics approval reported | Design fits research questions; purposive sampling; uses program data, time-motion study, and routine data. | text does not mention the identification of sources of bias or mitigation measures. | The authors’ conclusions are explicit and transparent. | No major flaws; findings align with literature; confidence in results strengthened | The results have practical applications in public health, potentially influencing health policies and interventions. |  |  |  |  |
|  | Reviewer's Score | 2 | 2 | 2 | 2 | 2 | 1 | 2 | 2 | 2 |  |  |  |  |
| Hu QH et al., 2021, China | **Rating** | **Yes**/No & Not sure/unclear | **Yes**/No & Not sure/unclear | **Yes**/No & Not sure/unclear | **Yes**/No & Not sure/unclear | **Yes**/No & Not sure/unclear | Yes/No & **Not sure/unclear** | **Yes**/No & Not sure/unclear | **Yes**/No & Not sure/unclear | **Yes**/No & Not sure/unclear | 16 | Low-risk | Included |  |
|  | Reviewer's response | Assisted PN for MSM HIV testing; key population; China context. | Clear study design and presentation | RCT with well-described methods | Ethical approval reported | Appropriate RCT methodology | Identifies disclosure, Hawthorne, ascertainment biases; Discusses mitigation strategies | Conclusions clear and transparent | Overall findings reliable but context-specific | Applicable to MSM testing; supports assisted PN & HIVST scale-up; informs key population policy. |  |  |  |  |
|  | Reviewer's Score | 2 | 2 | 2 | 2 | 2 | 2 | 1 | 1 | 2 |  |  |  |  |
| Goyette MS et al., 2018, Kenya | **Rating** | **Yes**/No & Not sure/unclear | **Yes**/No & Not sure/unclear | **Yes**/No & Not sure/unclear | **Yes**/No & Not sure/unclear | **Yes**/No & Not sure/unclear | Yes/No & **Not sure/unclear** | **Yes**/No & Not sure/unclear | **Yes**/No & Not sure/unclear | **Yes**/No & Not sure/unclear | 18 | Low-risk | Included |  |
|  | Reviewer's response | HIV partner notification, IPV impact, vulnerable populations | Well-structured, clear sections | Detailed RCT design, transparent analysis | IRB approvals, informed consent clearly stated | Cluster RCT suitable for research aim | Excluded recent IPV, IPV monitoring, bias acknowledged | Clear conclusion, reflective of results and limitations | Yes, Large sample, rigorous design, consistent results | Relevant to HIV partner notification in similar settings |  |  |  |  |
|  | Reviewer's Score | 2 | 2 | 2 | 2 | 2 | 2 | 2 | 2 | 2 |  |  |  |  |
| Maierhofer CN et al., 2023, Malawi | **Rating** | **Yes**/No & Not sure/unclear | **Yes**/No & Not sure/unclear | **Yes**/No & Not sure/unclear | **Yes**/No & Not sure/unclear | **Yes**/No & Not sure/unclear | Yes/No & **Not sure/unclear** | **Yes**/No & Not sure/unclear | **Yes**/No & Not sure/unclear | **Yes**/No & Not sure/unclear | 18 | Low-risk | Included |  |
|  | Reviewer's response | High HIV burden in Malawi; focus on partner notification, acute HIV detection, and key populations | Clear introduction, methods, results, and discussion sections | Well-described RCT, intervention vs. control arms, detailed outcomes | Ethical approvals from local and UNC IRBs; informed consent detailed | Cluster RCT suitable for intervention effect evaluation | Randomization, inclusion/exclusion criteria clear; some limitations discussed | Transparent conclusions with consideration of limitations | Large sample, rigorous statistical analysis, consistent results | Relevant for HIV partner services in resource-limited, high HIV prevalence settings |  |  |  |  |
|  | Reviewer's Score | 2 | 2 | 2 | 2 | 2 | 2 | 2 | 2 | 2 |  |  |  |  |
| Onovo A et al., 2021, Nigeria | **Rating** | **Yes**/No & Not sure/unclear | **Yes**/No & Not sure/unclear | **Yes**/No & Not sure/unclear | **Yes**/No & Not sure/unclear | **Yes**/No & Not sure/unclear | Yes/No & **Not sure/unclear** | **Yes**/No & Not sure/unclear | **Yes**/No & Not sure/unclear | **Yes**/No & Not sure/unclear | 17 | Low-risk | Included |  |
|  | Reviewer's response | Focus on index case testing and partner notification among key populations (FSW, MSM, PWID) in Nigeria, addressing HIV testing gaps | Clear description of background, methods, results, and discussion | Describes program data analysis, partner notification models, testing uptake, seropositivity rates, and linkage outcomes | Ethical approval obtained; routine program data analyzed | Use of large-scale program data; appropriate for feasibility and implementation assessment | Limitations acknowledged (lack of demographic/qualitative data); peer outreach model as strength | Conclusions clear, cautious, and consistent with data presented | Large sample size and disaggregated KP data supports findings; some data gaps limit deeper analysis | Highly applicable to HIV programs targeting key populations in resource-limited settings |  |  |  |  |
|  | Reviewer's Score | 2 | 2 | 2 | 2 | 2 | 1 | 2 | 2 | 2 |  |  |  |  |
| Remera E et al., 2022, Rwanda | **Rating** | **Yes**/No & Not sure/unclear | **Yes**/No & Not sure/unclear | **Yes**/No & Not sure/unclear | **Yes**/No & Not sure/unclear | **Yes**/No & Not sure/unclear | Yes/No & **Not sure/unclear** | **Yes**/No & Not sure/unclear | **Yes**/No & Not sure/unclear | **Yes**/No & Not sure/unclear | 15 | Low-risk | Included |  |
|  | Reviewer's response | Index testing to find older undiagnosed HIV+ in Rwanda | Clear abstract with objectives, methods, and findings | Logistic regression: partner notification data analysed | Ethics approval and informed consent reported | Appropriate for program data analysis | Limited info on bias; adjusted for key variables | Conclusions supported by data; focused on high-risk subgroups | Large sample; limited to abstract; generalizability unclear | Relevant for HIV programs targeting older adults in SSA |  |  |  |  |
|  | Reviewer's Score | 2 | 2 | 1 | 2 | 1 | 2 | 2 | 1 | 2 |  |  |  |  |
| Masters SH et al., 2016, Malawi | **Rating** | **Yes**/No & Not sure/unclear | **Yes**/No & Not sure/unclear | **Yes**/No & Not sure/unclear | **Yes**/No & Not sure/unclear | **Yes**/No & Not sure/unclear | Yes/No & **Not sure/unclear** | **Yes**/No & Not sure/unclear | **Yes**/No & Not sure/unclear | **Yes**/No & Not sure/unclear | 16 | Low-risk | Included |  |
|  | Reviewer's response | Index partner HIVST feasibility; male testing gap | Clear mixed-methods design and reporting | Well-described surveys, interviews, and analyses | Ethics approvals; informed consent | Appropriate mixed methods | Limitations noted; demonstration to reduce bias | Data-supported, cautious conclusions | Good sample; some limits on generalizability | Relevant to SSA HIV programs; addresses male barriers |  |  |  |  |
|  | Reviewer's Score | 2 | 2 | 2 | 2 | 2 | 1 | 2 | 1 | 2 |  |  |  |  |
