# Supplementary material for: HIV self-testing and partner notification strategies for key populations in low- to upper-middle-income countries: A mixed-methods systematic review
Source: PLoS One. 2025 Dec 29;20(12):e0338639. doi: 10.1371/journal.pone.0338639 (PMC12747344; doi:10.1371/journal.pone.0338639)
Supplement: S7 Table — (DOCX) [file pone.0338639.s007.docx]

**S7 Table: Article search results for Web Science Database**

| **#** | **Search Query** | **Database** | **Results** | **Date Run** |
| --- | --- | --- | --- | --- |
| 1 | TS=("HIV" OR "Human Immunodeficiency Virus") | Web of Science Core Collection | 463246 | Thu Oct 05, 2023, 17:59:28 GMT+0800 (Australian Western Standard Time) |
| 2 | TS=("AIDS" OR "acquired immunodeficiency syndrome") | Web of Science Core Collection | 238340 | Thu Oct 05, 2023, 19:00:42 GMT+0800 (Australian Western Standard Time) |
| 3 | #1 OR #2 | Web of Science Core Collection | 583020 | Thu Oct 05, 2023, 19:26:42 GMT+0800 (Australian Western Standard Time) |
| 4 | TS="Test*" | Web of Science Core Collection | 7135042 | Thu Oct 05, 2023, 19:28:04 GMT+0800 (Australian Western Standard Time) |
| 5 | TS="Self-test*" | Web of Science Core Collection | 8083 | Thu Oct 05, 2023, 19:28:43 GMT+0800 (Australian Western Standard Time) |
| 6 | TS="Self-administered test*" | Web of Science Core Collection | 112 | Thu Oct 05, 2023, 19:29:30 GMT+0800 (Australian Western Standard Time) |
| 7 | TS="Private test*" | Web of Science Core Collection | 122 | Thu Oct 05, 2023, 19:29:57 GMT+0800 (Australian Western Standard Time) |
| 8 | TS="At-home test*” | Web of Science Core Collection | 143 | Thu Oct 05, 2023, 19:30:19 GMT+0800 (Australian Western Standard Time) |
| 9 | TS=“Personal test*” | Web of Science Core Collection | 497 | Thu Oct 05, 2023, 19:30:37 GMT+0800 (Australian Western Standard Time) |
| 10 | TS=“Autonomy test*” | Web of Science Core Collection | 28 | Thu Oct 05, 2023, 19:30:51 GMT+0800 (Australian Western Standard Time) |
| 11 | TS=“Self-conducted test*” | Web of Science Core Collection | 3 | Thu Oct 05, 2023, 19:31:05 GMT+0800 (Australian Western Standard Time) |
| 12 | #4 OR #5 OR #6 OR #7 OR #8 OR #9 OR #10 OR #11 | Web of Science Core Collection | 7135042 | Thu Oct 05, 2023, 19:31:34 GMT+0800 (Australian Western Standard Time) |
| 13 | TS=“Partner notification" | Web of Science Core Collection | 1329 | Thu Oct 05, 2023, 19:32:02 GMT+0800 (Australian Western Standard Time) |
| 14 | TS=“contact trac*" | Web of Science Core Collection | 5526 | Thu Oct 05, 2023, 19:32:17 GMT+0800 (Australian Western Standard Time) |
| 15 | TS="partner disclosure" | Web of Science Core Collection | 104 | Thu Oct 05, 2023, 19:32:35 GMT+0800 (Australian Western Standard Time) |
| 16 | TS="Index test*" | Web of Science Core Collection | 2909 | Thu Oct 05, 2023, 19:32:51 GMT+0800 (Australian Western Standard Time) |
| 17 | TS="partner delivered" | Web of Science Core Collection | 19 | Thu Oct 05, 2023, 19:33:05 GMT+0800 (Australian Western Standard Time) |
| 18 | TS="partner test*" | Web of Science Core Collection | 218 | Thu Oct 05, 2023, 19:33:25 GMT+0800 (Australian Western Standard Time) |
| 19 | TS="Test* partner" | Web of Science Core Collection | 29 | Thu Oct 05, 2023, 19:33:39 GMT+0800 (Australian Western Standard Time) |
| 20 | TS="Partner service" | Web of Science Core Collection | 37 | Thu Oct 05, 2023, 19:33:56 GMT+0800 (Australian Western Standard Time) |
| 21 | TS=“partner referral” | Web of Science Core Collection | 69 | Thu Oct 05, 2023, 19:34:08 GMT+0800 (Australian Western Standard Time) |
| 22 | TS=“partner elicitation” | Web of Science Core Collection | 9 | Thu Oct 05, 2023, 19:34:22 GMT+0800 (Australian Western Standard Time) |
| 23 | TS=“sexual contacts” | Web of Science Core Collection | 885 | Thu Oct 05, 2023, 19:34:38 GMT+0800 (Australian Western Standard Time) |
| 24 | TS=“tracing sexual contacts” | Web of Science Core Collection | 3 | Thu Oct 05, 2023, 19:34:52 GMT+0800 (Australian Western Standard Time) |
| 25 | TS=“identification of contacts” | Web of Science Core Collection | 29 | Thu Oct 05, 2023, 19:35:21 GMT+0800 (Australian Western Standard Time) |
| 26 | TS=“tracing exposed partners” | Web of Science Core Collection | 0 | Thu Oct 05, 2023, 19:35:34 GMT+0800 (Australian Western Standard Time) |
| 27 | #13 OR #14 OR #15 OR #16 OR #17 OR #18 OR #19 OR #20 OR #21 OR #22 OR #23 OR #24 OR #25 OR #26 | Web of Science Core Collection | 10822 | Thu Oct 05, 2023, 19:36:41 GMT+0800 (Australian Western Standard Time) |
| 28 | #3 AND #12 AND #27 | Web of Science Core Collection | 939 | Thu Oct 05, 2023, 19:37:54 GMT+0800 (Australian Western Standard Time) |
| 29 | #3 AND #12 AND #27 Timespan: 2016-01-01 to 2023-12-30 | Web of Science Core Collection | **561** | Thu Oct 05, 2023, 19:41:41 GMT+0800 (Australian Western Standard Time) |
