# Supplementary material for: HIV self-testing and partner notification strategies for key populations in low- to upper-middle-income countries: A mixed-methods systematic review
Source: PLoS One. 2025 Dec 29;20(12):e0338639. doi: 10.1371/journal.pone.0338639 (PMC12747344; doi:10.1371/journal.pone.0338639)
Supplement: Attachment — Submitted filename: Response to Reviewer.docx [file pone.0338639.s008.docx]

**Response to Reviewers**

Dear Chief Editor,

Thank you for considering our manuscript for publication. We are grateful for your detailed editorial feedback and guidance, and the reviewer’s comments, which have helped to strengthen the scope and quality of our paper. Please see our response below.

**A: Response to Editor’s comment**

| **S/N** | **Editorial comments** | **Author response** |
| --- | --- | --- |
| 1. | Please ensure that your manuscript meets PLOS ONE's style requirements, including those for file naming. | Thank you for the reminder. We have reviewed and updated the manuscript and all associated files to ensure they meet PLOS ONE's style and file naming requirements. |
| 2. | We note that the grant information you provided in the ‘Funding Information’ and ‘Financial Disclosure’ sections does not match.  When you submit, please ensure that you provide the correct grant numbers for the awards you received for your study in the ‘Funding Information’ section. | Thank you for pointing out the inconsistency. We have reviewed and updated the grant information to ensure that the details in the ‘Funding Information’ and ‘Financial Disclosure’ sections now match.  Concerning the grant number, the PhD scholarship has no grant number. |
| 3. | Thank you for stating the following financial disclosure: [PhD Scholarship].  Please state what role the funders took in the study.  If the funders had no role, please state: "The funders had no role in study design, data collection and analysis, decision to publish, or preparation of the manuscript.""  If this statement is not correct you must amend it as needed.  Please include this amended Role of Funder statement in your cover letter; we will change the online submission form on your behalf. | Thank you for the comment. We have removed all funding references from the manuscript and included the revised Funding Statement and Role of the Funder information in the cover letter as requested.  **The text in the cover letter reads as.**  **………***The scholarship funder has no role in the overall study design, data collection and analysis, including the decision to publish and prepare the manuscript……* |
| 4 | We note that you have provided funding information that is not currently declared in your Funding Statement. However, funding information should not appear in the Acknowledgements section or other areas of your manuscript. We will only publish funding information present in the Funding Statement section of the online submission form.  Please remove any funding-related text from the manuscript and let us know how you would like to update your Funding Statement. Currently, your Funding Statement reads as follows:  [PhD Scholarship].  Please include your amended statements within your cover letter; we will change the online submission form on your behalf. | Thank you for your guidance. As requested, we have removed all funding-related text from the Acknowledgements section and the main body of the manuscript.  The amended statements within the cover letter read as follows.  *"….This study was supported by a PhD scholarship awarded to Lekey Khandu by the Australian Government Research Training Program (RTP) through Curtin University..."* |
| 5 | As required by our policy on Data Availability, please ensure your manuscript or supplementary information includes the following: All data extracted from each study for the reported systematic review and/or meta-analysis that would be needed to replicate your analyses. | Thank you for raising this. The manuscript has been edited and supplementary tables added to address this. |
| 5a | A numbered table of all studies identified in the literature search, including those that were excluded from the analyses. | Thank you for your comment. In response, we have developed and included a separate numbered table listing all full-text articles assessed for eligibility during the systematic review process. This table indicates which studies were included or excluded, and the reason. This information is presented in the supplementary table “**S4 Table. Full-text studies with inclusion and exclusion reasons**”  Accordingly, we have also updated the text under the heading **“Study Inclusion”** in the manuscript. The revised text reads as follows.  *……“Inclusion and exclusion reasons for full-text articles are detailed in Supplementary S4 Table.*  *[Please refer to, track change Line # [206-207].* |
| 5b | A table of all data extracted from the primary research sources for the systematic review and/or meta-analysis. The table must include the following information for each study:  Name of data extractors and date of data extraction  Confirmation that the study was eligible to be included in the review. | Thank you for your comment. The existing data extraction table initially submitted now includes the following parameters as follows;   a. Extractor names, b. Dates of extraction, and c. Eligibility confirmation for each included study.   All changes are highlighted in columns five to seven within the data extraction table, and this information is presented in the supplementary **“S5 Table. Characteristics of included studies”** |
| 5d | If applicable for your analysis, a table shows the completed risk of bias and quality/certainty assessments for each study or outcome.  Please ensure this is provided for each domain or parameter assessed. For example, if you used the Cochrane risk-of-bias tool for randomized trials, provide answers to each of the signalling questions for each study. If you used GRADE to assess the certainty of evidence, provide judgements about each of the quality of evidence factors. This should be provided for each outcome.  An explanation of how missing data were handled. | As described in the methods, we used the specific public health tools MetaQAT to evaluate each included study’s relevance, reliability, validity, and applicability. We assessed the methodological quality and risk of bias for each included study. This information was initially presented as as supplementary file. **“S6 Table. Quality appraisal and risk of bias (ROB)”**  However, as suggested we have updated the MetaQAT table with an additional “Risk of Bias” (ROB) column categorizing each study into low, moderate and high-risk categories.  The updated text under the heading **“Methodological quality appraisal”** in the Methodology of the manuscript now reads as follows;  *………”Each Study’s risk of bias (ROB) was assessed using the MetaQAT tool, scoring nine criteria from 0 to 2 (maximum 18). Studies were classified as low (15–18), moderate (11–14), or high risk of bias (≤10), consistent with similar reviews [27]. ROB ratings guided sensitivity analyses and evidence interpretation” ……*  *[Please refer to, track change Line # [169-172].*  Also, the subsequent updates were made for the heading **“Methodological quality and risk of bias”** in the result section of the manuscript and now reads as follows.  *….“Most studies (n = 72; 95%) were rated as low risk, indicating high methodological quality, with clearly defined objectives, appropriate study designs, and transparent reporting, which support the reliability and consistency of the findings for public health interpretation. A small number assessed as having moderate risk (n = 4; 5%). Studies with moderate-risk typically exhibited limitations of self-reporting or selection bias and incomplete bias mitigation descriptions. Accordingly, all studies were retained for data extraction to support a comprehensive comparison of methods”….*  *[Please refer to, track change Line # [234-240].* |

**B: Response to reviewer**

| **Reviewer comments** | **Author response** |
| --- | --- |
| 1.1. Title: The current title is excessively long. I suggest revising it to: "Acceptability and feasibility of HIV self-testing for partner notification in low- to upper-middle-income countries: A mixed-methods systematic review. | Thank you for your helpful suggestion. We agree that a more concise title would improve clarity. At the same time, we aim to retain the study’s core focus on key populations and index case partners, as reflected in our inclusion criteria and research questions.  Accordingly, we have revised the study title to:  “*HIV self-testing and partner notification strategies for key populations in low- to upper-middle-income countries: A mixed-methods systematic review*.”  *[Please refer to, track change Line # [1-3].* |
| Line 101: The connection between question 2 and HIV self-testing (HIVST), as announced in the title, is unclear. This link should be clarified, or the objective should be omitted. The overall objective, as stated in the discussion (e.g., lines 419 or 530–531), focuses on assessing the acceptability and feasibility of HIVST for partner notification (PN). Including question 2 dilutes the focus of the paper. | Thank you for this observation. To address this, based on the updated study title as indicated above, we have clarified the overall aim of the study as shown below.    *“Overall aim: This mixed-methods systematic review aims to assess the acceptability and feasibility of index HIVST and other partner notification strategies for reaching sexual and drug-injecting partners of index cases and other undiagnosed key populations in low- to upper-middle-income countries”*  *[Please refer to, track change Line # [102-105].* |
| • Line 263: Some results suggest that pPR (partner-provided referral) is also related to HIVST. Why dedicate one question solely to assisted partner notification (aPN) without addressing pPR? This inconsistency should be resolved. See my previous comment. | Thank you for this important observation. We acknowledge that the original phrasing of RQ2 focused exclusively on aPN and its approaches. At the same time, the results section includes evidence on multiple partner notification strategies/approaches involving HIVST, including pPR and aPN, contact referral, and information-assisted models. This has created an unintended inconsistency between the scope of our RQ2, and the breadth of evidence synthesised.  To address this, we have adjusted RQ2 as shown below.  *“What is the effectiveness of aPN and other partner notification strategies with and without HIVST, among index cases and undiagnosed MSM, TG persons, FSWs, and PWID?”*  *[Please refer to, track change Line #.[110-112].* |
| • Introduction: The overall objective of the paper should be explicitly stated in the introduction for clarity. | The overall aim has been clarified as requested (please see above response for *Line # [102-105]).* |
| 1.3. Previous Literature: • Line 114-116: Be specific about what the novel is in your review. Other systematic reviews have already covered some aspects, including the acceptability and use of HIVST (e.g., link 1, link 2) and feasibility (e.g., link 3). Highlight explicitly how your work differs or complements these studies. | Thank you for this important clarification. We acknowledge that previous systematic reviews have examined the general acceptability and feasibility of HIVST, particularly regarding individual-level use and community-based delivery models.  However, our review differs in both focus and scope as it uniquely examines the use of HIVST specifically for partner notification, particularly in the context of index testing among people diagnosed with HIV, and for secondary distribution by undiagnosed key populations.  The revised sentence in the manuscript under the heading **“Inclusion criteria”** now reads as follows:  *“….While prior reviews explored general HIVST acceptability and few have assessed HIVST and aPN separately [Ref], none have systematically compared HIVST and aPN as index testing strategies for partner notification using a mixed-methods approach across diverse populations in low- to upper-middle-income countries...”*  *[Please refer to, track change Line # [134-137].* |
| 1.4. Study Methods: • Line 122-125: o Consider mentioning how you treated mixed-method studies explicitly. o The statement "The qualitative components examined barriers and facilitators for index testing for PNS" raises questions. Is this focus on qualitative research due to a lack of quantitative studies? The JBI mixed-method systematic review manual (e.g., JBI guide) shows that barriers and enablers can also be addressed quantitatively. The authors should explain the divide between qualitative and quantitative approaches for each component of the research. | Thank you for this helpful comment. We have now clarified how mixed-methods studies were treated in the analysis.  Accordingly, we revised the text under the heading **“Eligibility Criteria”**, which read as follows.  *”….Both the quantitative and qualitative components examined barriers, facilitators and social harms for index testing for PNS. Furthermore, qualitative and quantitative data from mixed methods studies were extracted and analysed separately following the JBI convergent segregated approach.”*  *[Please refer to, track change Line #[144-147].* |
| Line 129: The term "secondary distribution" is commonly used in studies on key populations for partner testing. Excluding this term from the search may have caused you to miss relevant publications, such as those from the ATLAS project (link to ATLAS publications), which focus on distribution to partners of both diagnosed and undiagnosed key populations. | Thank you for this helpful observation. This may indeed have limited the retrieval of certain relevant studies, including those related to the ATLAS project.  We have now updated the ***limitations section*** of the manuscript to reflect this oversight and to acknowledge the potential for missed studies due to search term variability. The revised sentence in the limitation section reads as follows:  *“….Furthermore, search did not explicitly include the term “secondary distribution” which may have resulted in the exclusion of studies related to HIVST distribution by key populations to their partners….”*  *[Please refer to, track change Line # [620-622].* |
| 1.6. Heterogeneity: • Line 161: Clarify the sources of heterogeneity identified. | Thank you for this helpful suggestion. We have revised the manuscript to explicitly state the sources of heterogeneity that led to the decision not to conduct a meta-analysis.  We have added text under the heading **“Data Synthesis and Integration”,** and it reads as follows:  *“….Sources of heterogeneity included differences in study design, population groups (e.g., PLHIV, MSM, FSW, PWID), HIVST distribution strategies (e.g., aPN, partner-provided referral), measured outcomes (e.g., uptake, positivity rate, linkage), and geographic or health system contexts across low- to upper-middle-income countries.”…….*  *[Please refer to, track change Line # [187-191].* |
| 1.6. Heterogeneity:  • Line 217: Author states that no sensitivity analyses or investigations into heterogeneity | We agree that clarification is needed. Since a meta-analysis was not conducted due to the above-described heterogeneity, no statistical investigation of heterogeneity (e.g., sensitivity analysis or subgroup analysis) was applicable.  We have revised the text under the heading **“Methodological Quality and Risk of Bias”**  *“….Given the clinical and methodological heterogeneity across studies, such as differences in populations, study designs, and HIVST distribution approaches, sensitivity analysis and statistical assessment of the heterogeneity were not conducted.”*  *[Please refer to, track change Line # [242-244].* |
| 1.7. Subsection Relevance: • Line 273: The relevance of the section “Methods for index testing to PN” is unclear. Given Question 1, studies comparing HIVST to facility-based methods should be analyzed together. | Thank you for this observation. This concern is being addressed with the revision of study title, overall aim and RQ2.  We have also rephrased this section heading to align with the revised title, overall aim and RQ2. It now reads as follows.  ***“Partner notification strategies in index testing without HIVST”.***  *[Please refer to, track change Line # [334].* |
| 1.7. Subsection Relevance:  • Line 307-316: This section’s relevance to the research questions is unclear and could be omitted. | Thank you for this observation. We respectfully clarify that this section (Lines 307–316) is relevant to the overall study though not specifically linked any cost effectiveness RQ.  The included findings on cost-effectiveness provide important comparative insights for decision-makers considering resource allocation in partner notification strategies. Therefore, we have retained this section to support a more complete analysis of economic implications as a part of barriers and facilitators for implementing partner notification services. This provides a holistic picture of the feasibility of index HIVST and aPN. |
| 1.8. Table 2: The table should be restructured to align with the three research questions of the paper. | Thank you very much for the comments. The table provides a key summary of the overall findings of the review. It is follows the sequences of our result section that are now aligned with revised title, aim and RQ2.  *[Please refer to, track change Line # [480-481].* |
| 1.9. Language and Statements: • Line 527: The authors state that "non-English languages were considered," but only English terms were used in the search. This statement is misleading and should be removed. | Thank you for this important clarification. To address this, we have added a sentence under the limitations section under the heading **“Discussion”**. The updated text reads:  *“….This review was limited to studies published in English, and while no non-English studies were encountered during screening, relevant studies in other languages may have been missed….”*  *[Please refer to, track change Line # [618-620].* |
| • Line 528-529: Clarify how this aspect of the methodology was implemented. | Thank you for this observation. We confirm that all eligible studies were included regardless of quality rating to ensure a comprehensive synthesis. However, studies with stronger methodological designs, such as RCTs, quasi-experimental and large-scale observational studies, were given greater interpretive weight during synthesis, particularly when findings across studies differ. We have used the methodological quality appraisal outcomes to help explain the effectiveness of the results. |
| 2. Minor Comments 2.1. Line 75-77: Add a transition sentence to improve the connection between the two paragraphs. | Thank you for the suggestion. We have added a transition sentence at the beginning of the second paragraph to improve flow and link the discussion of HIVST uptake to the focus on partner notification and index testing approaches. The revised second paragraph under the heading **“Introduction”** now starts with.  *“Given these developments, integrating HIVST into PN strategies represents a promising yet underexplored opportunity to enhance testing coverage and linkage to care. Building on the growing role of HIVST among key populations, it is essential to explore how HIVST can be integrated into partner notification strategies through index testing approaches………….”*  *[Please refer to, track change Line # [78-81].* |
| 2.2. Abbreviations: Reduce the number of abbreviations to improve readability, keeping only those that are frequently used in the text. | Thank you for this helpful suggestion. We have reviewed the manuscript and reduced the number of abbreviations by retaining only frequently used ones. |
| Authorship Details: In the methods section, it is unusual to specify which author performed which tasks. Instead, mention the number of authors involved in each task when relevant, without using initials. | Thank you for the suggestion. As suggested, we have revised the methods section to remove author initials and instead described the number of reviewers involved in each task.  *[Please refer to, track change Line # [159, 166, 167 & 180].* |
| 2.4. Line 242: This sentence is unclear and should be rewritten for better comprehension. | Thank you for your observation. We have revised the sentence and now it reads as follows;  *“Twenty-one studies [Ref] reported on the effectiveness of aPN in increasing partner testing uptake, new diagnosis, and linkages to care”….*  *[Please refer to, track change Line # [268-269].* |
| 2.5. Line 433: The term "ART cases" is ambiguous. Define "ART" at its first use and clarify its meaning in this context. | Thank you for pointing this out. We have now clarified the term “ART” (antiretroviral therapy) at its first mention in the manuscript.  *[Please refer to, track change Line # [498].* |
| 2.6. Line 520: The phrase "the fidelity of aPN" is unclear and should be rephrased or elaborated. | Thank you for this helpful observation. We have revised the sentence for clarity under the heading “Discussion”. The revised sentence now reads as follows:  *“….This review found that passive partner referral (pPR) and interactions with health facilities may influence the consistency and quality of assisted partner notification (aPN) implementation, as well as partner-delivered HIVST….”*  *[Please refer to, track change Line # [599-601].* |
| 2.7. Line 522: The "Strengths and Limitations" subsection appears to be the only subsection within the discussion. Consider removing the subsection title and integrating the content into the main discussion. | Thank you for this suggestion. We have removed the “Strengths and Limitations” subheading and integrated its content into the main discussion.  *[Please refer to, track change Line # [604].* |
| 2.8. Line 525: The statement about "inadequate information among non-married and undiagnosed key populations" is vague. If this is a gap in literature rather than a limitation of the current study, it should not be listed as a limitation of the paper. | Thank you for this important clarification. We agree that the lack of data concerning non-married and undiagnosed key populations reflects a broader gap in the literature rather than a methodological limitation of this review. We have removed the statement from the limitations section and integrated the observation within the main discussion to frame it as a literature gap.  The revised text under the heading “Discussion” read as follows;  *..“In addition, the review identified a significant gap in the literature regarding unmarried individuals and undiagnosed key populations, particularly those on ART who have not disclosed their HIV status. These groups are often underrepresented in studies exploring the feasibility and acceptability of index HIVST for partner notification. Future research should prioritize understanding the unique barriers and needs of these populations to ensure inclusive and effective partner services”..*  *[Please refer to, track change Line # [507-512].* |
