# Supplementary material for: HIV self-testing and partner notification strategies for key populations in low- to upper-middle-income countries: A mixed-methods systematic review
Source: PLoS One. 2025 Dec 29;20(12):e0338639. doi: 10.1371/journal.pone.0338639 (PMC12747344; doi:10.1371/journal.pone.0338639)
Supplement: Attachment — Submitted filename: Comments PONE-D-24-43591R1.docx [file pone.0338639.s009.docx]

On Introduction

**1. Clear Problem Statement**

The introduction outlines obstacles to HIV testing and presents HIVST, but it does not explicitly specify the issue this review aims to address.

Missing: a clearer assertion such as “Despite evidence supporting HIVST effectiveness, there is limited knowledge about how it is integrated into partner notification services in LMICs, especially among key populations.”

**2. Justification for Mixed-Methods Approach**

You highlight the importance of HIVST and PN individually, but you do not clarify why a mixed-methods systematic review is the most suitable approach.

Missing: a justification explaining that quantitative studies offer data on uptake and effectiveness, whereas qualitative studies identify barriers, facilitators, and contextual details.

**3. Knowledge Gaps**

The introduction mentions a lack of clarity regarding acceptability and feasibility, but it does not explicitly specify what is missing in the existing literature.

- There are few studies focusing on unmarried people living with HIV.
- Limited evidence from Asia is noted later in the discussion, but not initially.
- Limited understanding of policy and legal environments in LMICs.
- Limited examination of risks such as IPV, stigma, and disclosure safety.

**4. Significance and Policy Relevance**

The introduction fails to highlight why this is important at this moment.

- HIVST and PN are priority strategies in WHO’s 95-95-95 targets.
- Many LMICs are scaling up HIVST, but integration into PN remains inconsistent.

Missing a clear connection to global health goals (UNAIDS, WHO) to emphasize relevance.

**5. Conceptual Framework**

PN approaches (passive, assisted, dual referral) are described, but the introduction lacks mention of an analytic framework (such as RE-AIM, CFIR, or the socio-ecological model) that will inform the interpretation of barriers and facilitators.

Without this, the research questions seem more descriptive and less focused on theory.

**6. Specificity of Research Questions**

Current questions are pretty broad:

- Uptake of HIVST vs. facility testing.
- Effectiveness of aPN.
- Barriers/facilitators.

Missing: clarification on which outcomes (acceptability, feasibility, safety, linkage to care, cost-effectiveness). Aligning the questions with your stated objectives would refine the scope.

**On Methods**

**1. Protocol & Registration**

Lacking: The text does not mention PROSPERO registration or the availability of the protocol.

Recommendation: Indicate whether the review was registered prospectively, including the registration ID, or, if not, provide an explanation. Journals increasingly require this information.

**2. Search Strategy Transparency**

Lacking: Only PubMed, Embase, CINAHL, and Web of Science are listed. Regional LMIC databases such as LILACS, African Index Medicus, IndMed, trial registries, and grey literature sources like WHO, UNAIDS, and PEPFAR reports are absent.

Recommendation: Broaden your source selection and include a justification. Add the full electronic search strategy (for at least one database) in an appendix, in accordance with PRISMA-S guidelines.

**3. Timeframe & Language**

It is unclear whether there were any date or language restrictions, which reviewers could see as a bias risk. To improve clarity, clearly state the start and end years and describe how non-English studies were handled—either by translation or exclusion.

**4. Screening & Selection Process**

Unclear how many reviewers screened the studies and how disagreements were addressed. Recommend stating that two independent reviewers carried out screening and data extraction, with a third reviewer resolving any disagreements. Also, mention if a pilot calibration step was included.

**5. Data Extraction & Management**

Missing: No mention of extraction tools or software, verification procedures, or how to handle multiple reports of the same study.

Recommendation: Specify data management tools like Covidence, Rayyan, or Excel, detail piloting extraction forms, and explain the process for resolving discrepancies.

**6. Risk of Bias / Quality Assessment**

Lacking: It seems only CASP was applied to qualitative studies, but it is not clear for quantitative ones (RCTs, observational).

Recommendation:

Use RoB 2 for RCTs, ROBINS-I for non-randomized studies, and CASP or JBI for qualitative research. For mixed-methods studies, MMAT is suitable.State whether appraisal was done independently by ≥2 reviewers.

**7. Data Synthesis Approach**

Missing: Overall narrative or thematic description, but it's unclear how quantitative and qualitative data were combined—was it parallel, convergent, or explanatory sequential?

Recommendation:

For quantitative data, clarify the criteria for pooling, such as using a random-effects meta-analysis if outcomes are comparable; otherwise, consider a narrative synthesis following SWiM guidelines.

For qualitative: specify method (thematic synthesis vs. meta-aggregation).

For integration: name the approach (e.g., convergent segregated design) and whether joint displays were used.

**8. Outcome Definitions**

Lacking: Acceptability and feasibility are mentioned but not clearly defined, which could lead to inconsistency during data extraction.

Recommendation: Clearly specify outcomes beforehand, such as defining acceptability as willingness or uptake, and feasibility as the proportion completing the test, linkage success, or logistical considerations. Also, describe how adverse events like IPV and stigma were managed.

**9. Equity and Context**

Lacking: No clear plan for analyzing results across population subgroups (MSM, PWID, sex workers, adolescents) or by country income and policy environment.

Recommendation: Clearly specify subgroup or sensitivity analyses at the beginning and include discussion of the policy/legal context as part of feasibility considerations.

**10. Certainty of Evidence**

Missing: No reference to GRADE (quantitative) or GRADE-CERQual (qualitative). Recommendation: Develop a clear plan to assess confidence in the findings, as this is now a requirement for many journals.

**On Study Results**

**1. Clarity and Structure**

The section is lengthy and detailed, but the results are not consistently organized according to your stated objectives (acceptability, feasibility, effectiveness, barriers/facilitators).

Recommendation: Reorganize the results under subheadings that align with the research questions to improve clarity and help readers follow the discussion more easily.

**2. Study Characteristics Summary**

You mention country distribution and study designs, but lack a concise comparative summary, such as a table contrasting sample sizes, populations, interventions, and outcomes.

Recommendation: Include a summary table or figure that highlights study designs, populations (e.g., MSM, PWID, FSWs), notification approaches, and HIVST delivery models.

**3. Equity and Subgroup Analyses**

While key populations such as MSM, FSW, TG, and PWID are acknowledged, the synthesis lacks detailed analysis of results differentiated by group, gender, relationship type, or region.

Recommendation: Clearly specify if acceptability or feasibility varied among different populations, settings, or income levels.

**4. Integration of Quantitative and Qualitative Findings**

Quantitative results and qualitative themes are presented separately, with limited integration.

Recommendation: Use joint displays or combined narratives to illustrate how qualitative data clarifies quantitative outcomes, such as reasons for higher uptake with self-testing or existing contextual barriers.

**5. Operationalization of Outcomes**

The terms "acceptability" and “feasibility” are mentioned, but the studies do not consistently explain how these outcomes were defined or measured.

Recommendation: Standardize reporting by clearly defining these terms, for example, acceptability as willingness or uptake, and feasibility in terms of implementation outcomes, such as linkage, costs, or fidelity.

**6. Assessment of Heterogeneity and Bias**

Recommendation: Even if a meta-analysis isn't conducted, utilize SWiM guidelines for narrative synthesis and address differences in populations, interventions, and outcomes.

**7. Certainty of Evidence**

No GRADE or CERQual assessment has been provided. Without this, readers cannot determine confidence in the findings.

Recommendation: Use GRADE for quantitative outcomes (such as uptake, linkage, IPV events) and CERQual for qualitative themes.

**8. Link to Policy/Practice**

Results are provided in detail, but policy implications, such as safety concerns and regulatory challenges, are reserved for the discussion section.

Recommendation: Briefly outline in the results section how the findings relate to implementation challenges, such as legal issues, social harms, and stigma.

**On the Discussions**

**1. Deeper Critical Interpretation**

Much of the discussion reiterates the results instead of providing critical analysis. Missing is an exploration of why specific findings occurred, such as cultural norms influencing women’s preference for pPR versus men’s reluctance to visit facilities.

**2. Equity and Key Populations**

While key populations (MSM, FSWs, PWID) are mentioned in the results, the discussion does not thoroughly examine differences in acceptability or feasibility based on gender, risk group, or relationship dynamics. It also lacks consideration of intersectionality aspects, such as stigma among MSM, safety risks faced by sex workers, and legal barriers encountered by PWID.

**3. Integration of Quantitative and Qualitative Evidence**

Findings are discussed separately, but there is no combined synthesis demonstrating how qualitative data clarifies quantitative results.

Missing: explicit integration using frameworks or joint interpretation (for example, uptake is higher in RCTs, but qualitative data shows the influence of trust, confidentiality, and gender dynamics).

**4. Policy and Implementation Context**

The text references cost and provider perspectives but lacks analysis of legal and policy environments, such as criminalization, consent laws, and WHO/UNAIDS guidelines. It also overlooks the explicit consideration of how policy contexts in LMICs influence feasibility and scalability.

**5. Comparison to Other Reviews/Guidelines**

The discussion does not compare its findings with existing systematic reviews, meta-analyses, or WHO guidelines. It also lacks a clear statement on how this review advances knowledge or differs from previous evidence syntheses.

**6. Strengths and Limitations**

A brief strengths/limitations note exists, but it underplays key issues:

- Lack of non-English studies.
- Exclusion of unpublished or ongoing trials.
- No sensitivity analyses or heterogeneity exploration.
- Absence of GRADE/CERQual certainty assessments.

**7. Implications for Future Research and Practice**

The section highlights the need for additional research in Asia but remains vague about future directions. It lacks concrete recommendations such as conducting trials with unmarried partners, developing interventions to address IPV risk, conducting implementation research in criminalized settings, and assessing cost-effectiveness in real-world programs.

**On Conclusions**

**1. Overly Descriptive, Not Interpretive**

The conclusion summaries the findings but lacks a strong final interpretation of what the evidence implies for policy, practice, and research. It also misses an interpretive “so what?” statement that clarifies the review's significance.

**2. No Explicit Policy or Program Implications**

The conclusion does not detail how the findings can inform HIV programs in LMICs, such as by integrating them into national HIV testing strategies, partner notification protocols, or assessing their cost-effectiveness for scaling up. It also omits practical advice for policymakers and implementers.

**3. Equity and Key Populations**

Although gaps are recognized for unmarried index cases and undiagnosed KPs, the conclusion does not emphasize the equity issues related to gender, stigma, criminalization, and marginalization. It also does not explicitly state that interventions should be designed with sensitivity to these factors.

**4. No Link to Global Targets**

The conclusion does not connect the findings to the UNAIDS 95-95-95 targets or WHO guidelines, reducing its global health relevance. It also fails to link the presented evidence with ongoing global HIV elimination initiatives.

**5. Future Research Agenda**

Although further research is acknowledged as necessary, the conclusion does not specify which particular study types are most needed—such as RCTs involving unmarried partners, IPV monitoring, implementation trials in Asia, or cost-effectiveness analyses of community-led programs. It also lacks clear, actionable priorities for future research.

**6. Strengths and Limitations Integration**

The conclusion does not address the review’s own limitations, such as regional bias towards Africa, the absence of a meta-analysis, and the exclusion of grey or non-English studies. It lacks a balanced summary that discusses how these limitations influence the interpretation of the findings.
