# Supplementary material for: HIV self-testing and partner notification strategies for key populations in low- to upper-middle-income countries: A mixed-methods systematic review
Source: PLoS One. 2025 Dec 29;20(12):e0338639. doi: 10.1371/journal.pone.0338639 (PMC12747344; doi:10.1371/journal.pone.0338639)
Supplement: Attachment — Submitted filename: Response to Reviewer_11_18_2025.docx [file pone.0338639.s010.docx]

**Response to the reviewer**

Dear Chief Editor,

Thank you for considering our manuscript for publication. We are grateful for the reviewers’ comments, which have helped strengthen the scope and quality of our paper. Please see our responses below.

| **S/N** | **Reviewer comment** | **Author response** |
| --- | --- | --- |
| 1. | **Clear Problem Statement**  The introduction outlines obstacles to HIV testing and presents HIVST, but it does not explicitly specify the issue this review aims to address.  Missing: a clearer assertion such as “Despite evidence supporting HIVST effectiveness, there is limited knowledge about how it is integrated into partner notification services in LMICs, especially among key populations.” | Thank you for your observation. We have added a sentence to meet the requirements as stated below.  *“…However, even with the evidence supporting HIVST effectiveness, there is limited understanding about how HIVST is integrated into partner notification services in lower to upper-middle-income countries, particularly among key populations…”*  ***[Please refer to track change lines #96-99]*** |
| 2. | **Justification for Mixed-Methods Approach**  You highlight the importance of HIVST and PN individually but do not clarify why a mixed-methods systematic review is the most suitable approach.  Missing: a justification explaining that quantitative studies offer data on uptake and effectiveness, whereas qualitative studies identify barriers, facilitators, and contextual details. | Thank you for this observation. The justification for using mixed methods is provided in the methods. Please see below.  *“…This mixed-methods systematic review study intends to use quantitative findings to assess the uptake and effectiveness,* whereas qualitative studies identify barriers, facilitators, and contextual details*…”*  ***[Please refer to track change lines #125-127]*** |
| 3. | **Knowledge Gaps**  The introduction mentions a lack of clarity regarding acceptability and feasibility, but it does not explicitly specify what is missing in the existing literature.   - There are a few studies focusing on unmarried people living with HIV. - Limited evidence from Asia is noted later in the discussion, but not initially. - Limited understanding of policy and legal environments in LMICs. - Limited examination of risks such as IPV, stigma, and disclosure safety. | Thank you for your valuable feedback. For clarification, we initially conducted a broad literature review related to the feasibility and acceptability of HIVST for index testing and partner notification to establish the context and rationale that informed the aims and objectives of the systematic review. Following the review, we identified the specific gaps and which are presented in the results and discussion section.  However, your concerns are also addressed in response # 4 below (**Significance and Policy Relevance).** |
| 4. | **Significance and Policy Relevance**  The introduction fails to highlight why this is important at this moment.   - HIVST and PN are priority strategies in WHO’s 95-95-95 targets. - Many LMICs are scaling up HIVST, but integration into PN remains inconsistent.   Missing a clear connection to global health goals (UNAIDS, WHO) to emphasize relevance. | Thank you for your observation. We have added the global target as stated below (edits highlighted in red).    *“…Despite partner notification’s long history in HIV management, integrating HIVST within partner notification services (PNS) may strengthen efforts to achieve the global 95-95-95 targets to end the HIV/AIDS epidemic. Evidence shows a high likelihood of HIV infection among sexual and drug-injecting partners of diagnosed PLHIV [15,16]…”*  ***[Please refer to track change lines #90-93]***  Thank you for your suggestion. We have added the limited scaling of HIVST and PN in LMICs to the revised manuscript, as stated below.  *“…Although many low and middle-income countries (LMICs) are scaling up HIVST, integration into PN remains inconsistent and limited [10,19]. However, even with the evidence supporting HIVST effectiveness, there is limited understanding about how HIVST is integrated into partner notification services in lower to upper-middle-income countries, particularly among key populations. Moreover, to date, there is no mixed-methods systematic review that has comprehensively covered strategic information on both the index HIVST and traditional facility-based PNS…”*  ***[Please refer to the track change line #94-101].*** |
| 5. | **Conceptual Framework**  PN approaches (passive, assisted, dual referral) are described, but the introduction lacks an analytic framework (such as RE-AIM, CFIR, or the socio-ecological model) that will inform the interpretation of barriers and facilitators.  Without this, the research questions seem more descriptive and less focused on theory. | Thank you for the comments. We have interpreted our review findings and clarified this under the Methods section. We used the JBI convergent segregated approach to interpret our review findings, as outlined below.  *“...Findings were interpreted across service delivery and implementation factors influencing the uptake of HIVST and aPN across different populations, relationship types and settings. Appropriate comparisons were made to understand the effectiveness of each partner notification model and its feasibility in terms of technical efficiency and cost for future adaptation to scale up HIV testing, and timely care linkages among key populations...”*  ***[Please refer to track change lines # 200-205]*** |
| 6. | **Specificity of Research Questions**  Current questions are pretty broad:   - Uptake of HIVST vs. facility testing. - Effectiveness of aPN. - Barriers/facilitators.   Missing: clarification on which outcomes (acceptability, feasibility, safety, linkage to care, cost-effectiveness). Aligning the questions with your stated objectives would refine the scope. | Thank you for your valuable feedback. The research questions are refined for clarity without deviating from their original concept to include the specific outcomes as stated below (edits highlighted in red).   1. What is the acceptability, feasibility, and level of uptake of HIVST for partner notification compared to facility-based testing? 2. What is the effectiveness of aPN and other partner notification strategies with and without HIVST in increasing partner testing, HIV case identification, and linkages to care among index cases and undiagnosed key populations? 3. What factors, including barriers and facilitators, influence the feasibility and safety of implementing HIVST and aPN within partner notification services, among index cases and undiagnosed key populations?   ***[Please refer to track change lines # 106-115]*** |
|  | **On Methods** |  |
| 1. | **Protocol & Registration**  Lacking: The text does not mention PROSPERO registration or the availability of the protocol.  Recommendation: Indicate whether the review was registered prospectively, including the registration ID, or, if not, provide an explanation. Journals increasingly require this information. | Thank you for your observation. We have included “***prospectively”*** in the revised manuscript as stated below;  *“…This review was registered prospectively with PROSPERO (CRD42023475417) [21] to ensure rigor and transparency…”*  ***[Please refer to track change line#121]*** |
| 2. | **Search Strategy Transparency**  Lacking: Only PubMed, Embase, CINAHL, and Web of Science are listed. Regional LMIC databases such as LILACS, African Index Medicus, IndMed, trial registries, and grey literature sources like WHO, UNAIDS, and PEPFAR reports are absent.  Recommendation: Broaden your source selection and include a justification. Add the full electronic search strategy (for at least one database) in an appendix, in accordance with PRISMA-S guidelines. | Thank you for your feedback. We understand the importance of the broader literature, including grey literature; however, we purposely used peer-reviewed journal articles to ensure scientific rigour, reliability and quality assurance. Therefore, we have retained our current research strategy and have acknowledged the limitations as stated below.  *….“The search strategy was limited to four databases, which may have missed some relevant studies from LMICs published in sources such as LILACS or African Index Medicus. No grey literature was reviewed, and we may have missed studies reported outside of the peer-reviewed literature. However, the reference lists of the selected articles were examined for additional eligible articles…”.*  ***[Please refer to track change lines# 666-670].***  As suggested, we have also included **one** full electronic search strategy as a supplementary file (S7 Table: Article search results for Web Science Database). |
| 3. | **Timeframe & Language**  It is unclear whether there were any date or language restrictions, which reviewers could see as a bias risk. To improve clarity, clearly state the start and end years and describe how non-English studies were handled—either by translation or exclusion. | Thank you. We have added the specific dates and language in the revised manuscript as stated below (edits highlighted in yellow).  *“Studies published in English between 1 January 2016 and 30 December 2023 were included to align with WHO guidelines on HIVST and partner notification.”*  ***[Please refer to track change line #137]***  We did not locate any **non-English articles.** The limitations section states that the review did not locate any non-English articles ***[see track change lines# 664-666]*** |
| 4. | **Screening & Selection Process**  Unclear how many reviewers screened the studies and how disagreements were addressed.  Recommend stating that two independent reviewers carried out screening and data extraction, with a third reviewer resolving any disagreements. Also, mention if a pilot calibration step was included. | Thank you. This information is already included in our manuscript under “Study selection”.  ***Please refer to track change lines #164-167***. The current lines state, *“After a pilot test, the first author (LK) screened titles and abstracts. The first author conducted a full-text review for all studies, and the second review was distributed randomly to other research team members. Disagreements were resolved through group discussion”.* |
| 5. | **Data Extraction & Management**  Missing: No mention of extraction tools or software, verification procedures, or how to handle multiple reports of the same study.  Recommendation: Specify data management tools like Covidence, Rayyan, or Excel, detail piloting extraction forms, and explain the process for resolving discrepancies. | Thank you for highlighting this. Our manuscript describes using Rayyan as a data extraction tool and Microsoft Excel for creating a charting table under the heading “Data extraction/collection”.  ***[Please refer to track change lines #180-184].*** |
| 6. | **Risk of Bias/Quality Assessment**  Lacking: It seems that only CASP was applied to qualitative studies, but it is unclear for quantitative ones (RCTs, observational studies).  Recommendation:  Use RoB 2 for RCTs, ROBINS-I for non-randomized studies, and CASP or JBI for qualitative research. For mixed-methods studies, MMAT is suitable. State whether the appraisal was done independently by ≥2 reviewers. | Thank you for your feedback. There appears to be some confusion, as CASP was not used in this study. Rather, we used the MetaQAT as a tool for quality appraisal for all the selected studies. This has been previously used by the authors in other published systematic reviews.  Concerning the methodological appraisal, our manuscript states *“…first author assessed all selected studies and* ***ten per cent*** *of articles were randomly selected by two other authors and assessed independently…”.*  This information can be found under “Methodological quality appraisal”  ***[Please refer to track change lines #172-173]*** |
| 7. | **Data Synthesis Approach**  Missing: Overall narrative or thematic description, but it's unclear how quantitative and qualitative data were combined—was it parallel, convergent, or explanatory sequential?  Recommendation:  For quantitative data, clarify the criteria for pooling, such as using a random-effects meta-analysis if outcomes are comparable; otherwise, consider a narrative synthesis following SWiM guidelines.  For qualitative: specify method (thematic synthesis vs. meta-aggregation).  For integration: name the approach (e.g., convergent segregated design) and whether joint displays were used. | We would like to clarify that our manuscript describes data synthesis following the JBI convergent segregated approach. We have presented an excerpt from the manuscript below for your reference*.* ***[Please see track change lines # 187-193].***  *“Following the JBI convergent segregated approach [33], qualitative data were synthesized independently before integration. Quantitative data were synthesized narratively using descriptive and inferential statistics, without meta-analysis due to heterogeneity. Sources of heterogeneity included differences in study design, population groups (e.g., PLHIV, MSM, FSW, PWID), HIVST distribution strategies (e.g., aPN, partner-provided referral), measured outcomes (e.g., uptake, positivity rate, linkage), and geographic or health system contexts across low- to upper-middle-income countries”*  Therefore, no changes have been made to the manuscript. |
| 8. | **Outcome Definitions**  Lacking: Acceptability and feasibility are mentioned but not clearly defined, which could lead to inconsistency during data extraction.  Recommendation: Clearly specify outcomes beforehand, such as defining acceptability as willingness or uptake, and feasibility as the proportion completing the test, linkage success, or logistical considerations. Also, describe how adverse events like IPV and stigma were managed. | We appreciate your observations. The outcome definition is now included after the eligibility criteria under a new heading, **“Data outcome definition”,** as stated below.  *“Aligned with the review questions, the outcomes were operationally defined according to the existing literature [29]. Acceptability was defined as the proportion of individuals who self-tested among those offered testing [29]. At the same time, uptake was computed as acceptability and the actual HIVST use percentage. Feasibility was defined as the completion of HIVST and counselling as a result of ease of use, correct interpretation of results, and links to care [29]. The effectiveness is considered a key outcome of partner notification, including partner testing uptake, new diagnosis and linkage to care among the index and their partners.”*  ***[Please refer to track change lines #145-153]*** |
| 9. | **Equity and Context**  Lacking: No clear plan for analyzing results across population subgroups (MSM, PWID, sex workers, adolescents) or by country income and policy environment.  Recommendation: Clearly specify subgroup or sensitivity analyses at the beginning and include a discussion of the policy/legal context as part of feasibility considerations. | Thank you for the comments. We have added a small paragraph under “Data synthesis and integration” as per below.  *“...The findings related to subgroup populations (MSM, FSW, PWID, transgender persons, and index clients) were analyzed narratively to identify characteristic differences, while the regional context was considered to understand the equity-related differences in accessibility and feasibility...”*  ***[Please refer to track change lines #206-209].*** |
| 10. | **Certainty of Evidence**  Missing: No reference to GRADE (quantitative) or GRADE-CERQual (qualitative).  Recommendation: Develop a clear plan to assess confidence in the findings, as this is now a requirement for many journals. | Thank you for the comment. We used the MetaQAT tool to assess methodological quality of the included studies, as stated below.  The current text states *“…Methodological quality was assessed using questions under each domain (relevance, reliability, validity, and applicability). Each study’s risk of bias (ROB) was assessed using the MetaQAT tool, scoring nine criteria from 0 to 2 (maximum 18). Studies were classified as low (15–18), moderate (11–14), or high risk of bias (≤10), consistent with similar reviews [31]…”*  *Therefore, no changes have been made to the manuscript.*  ***[Please refer to track change lines #173-177]*** |
|  | **On Study Results** |  |
| 1. | **Clarity and Structure**  The section is lengthy and detailed, but the results are not consistently organized according to your stated objectives (acceptability, feasibility, effectiveness, barriers/facilitators).  Recommendation: Reorganize the results under subheadings that align with the research questions to improve clarity and help readers follow the discussion more easily. | Thank you for your suggestions. We have made edits to the following subheading of the quantitative results to align with the research questions as stated below.   1. Acceptability, feasibility and uptake of HIVST for partner notification compared with facility-based testing. ***[Please see track change lines 256-257]***   2. Effectiveness of aPN services. ***[Please refer to line 278]***   3. Partner notification strategies with HIVST. ***[Please refer to track change lines #308]*** 4. Partner notification strategies without HIVST. ***[Please refer to track change lines #345]***   5. Barriers, facilitators, and social harms associated with HIVST for partner testing. ***[Please refer to track change lines # 368-369]*** |
| 2. | **Study Characteristics Summary**  You mention country distribution and study designs, but lack a concise comparative summary, such as a table contrasting sample sizes, populations, interventions, and outcomes.  Recommendation: Include a summary table or figure that highlights study designs, populations (e.g., MSM, PWID, FSWs), notification approaches, and HIVST delivery models. | Please see the study characteristics summary table provided (refer to file **S5 Table. Characteristics of included studies**)  ***[Please refer to track change line # 223]*** |
| 3. | **Equity and Subgroup Analyses**  While key populations such as MSM, FSW, TG, and PWID are acknowledged, the synthesis lacks detailed analysis of results differentiated by group, gender, relationship type, or region.  Recommendation: Clearly specify if acceptability or feasibility varied among different populations, settings, or income levels. | Thank you. We appreciate your valuable feedback. We have included a new paragraph in the *discussion* section as per below.  *“…Differences in acceptability and feasibility vary by population, gender, and type of relationship. This shows the intersections of stigma, safety, and the legal environment among MSM and FSW, hindering disclosure and partner notification. For the women living with HIV, privacy and safety shaped their preferences for primary versus casual sexual partners. For PWID, legal and policy barriers inhibited their participation in index testing and partner notification. This highlights the importance of implementing rights-sensitive and context-specific HIVST and partner notification services, particularly in LMICs…”*  ***[Please refer to track change lines #643-650].*** |
| 4. | **Integration of Quantitative and Qualitative Findings**  Quantitative results and qualitative themes are presented separately, with limited integration.  Recommendation: Use joint displays or combined narratives to illustrate how qualitative data clarifies quantitative outcomes, such as reasons for higher uptake with self-testing or existing contextual barriers. | Thank you for the suggestion. We kept the data integration and synthesis section brief by highlighting key quantitative and qualitative findings to avoid repetition in the discussion section. In addition, the summary Table 2 highlights the combined interpretation of both types of data, providing a clear overview of how quantitative data clarifies qualitative outcomes such as higher HIVST uptake, contextual barriers and implementation feasibility. This aligns with the JBI convergent-segregated approach for the mixed-methods review. We have left the current structure to ensure JBI methodological consistency.  However, to ensure greater consistency with the text, we revised the title of the table as stated below: “Table 2. Summary of integration of quantitative and qualitative systematic review findings”  ***[Please refer to track change lines #500-501]*** |
| 5 | **Operationalization of Outcomes**  The terms "acceptability" and “feasibility” are mentioned, but the studies do not consistently explain how these outcomes were defined or measured.  Recommendation: Standardize reporting by clearly defining these terms, for example, acceptability as willingness or uptake, and feasibility in terms of implementation outcomes, such as linkage, costs, or fidelity. | Thanks. We have addressed these comments earlier under the Methods section and provided definitions  ***[Please refer to track change lines #145-153]*** |
| 6 | **Assessment of Heterogeneity and Bias**  Recommendation: Even if a meta-analysis isn't conducted, utilise SWiM guidelines for narrative synthesis and address differences in populations, interventions, and outcomes. | Thank you for your recommendation we would like to highlight that we followed JBI mixed-methods, MetaQAT, and meta-aggregation frameworks to ensure transparent assessment and synthesis consistent with guidelines such as SWiM Therefore, we have left the manuscript unchanged. |
| 7 | **Certainty of Evidence**  No GRADE or CERQual assessment has been provided. Without this, readers cannot determine confidence in the findings.  Recommendation: Use GRADE for quantitative outcomes (such as uptake, linkage, IPV events) and CERQual for qualitative themes. | Thank you for your feedback. We appreciate the importance of the tools that you suggested. However, as explained earlier, our study used a well-established JBI mixed-methods design following a convergent-segregated approach. We also used the MetaQAT to conduct a quality appraisal of all the included studies **[please refer to track change lines #*169-178]*.** Furthermore, the credibility of qualitative findings is assessed using meta-aggregation methods, labelling the results into three categories (univocal, equivocal, and unsupported) ***[please refer to track change lines 198-199].*** These approaches provided a comprehensive and transparent appraisal of the evidence, consistent with the domains of GRADE and CERQual. |
| 8 | **Link to Policy/Practice**  Results are provided in detail, but policy implications, such as safety concerns and regulatory challenges, are reserved for the discussion section.  Recommendation: Briefly outline in the results section how the findings relate to implementation challenges, such as legal issues, social harms, and stigma. | Thank you. We appreciate your observation. We have signposted these findings at the end of the qualitative section, providing a full interpretation and implications in the *Discussion* section.  *“The findings related to stigma, intimate partner violence (IPV), gender norms, and provider constraints indicate the presence of contextual and policy-related factors influencing implementation”*  ***[Please refer to track change lines #481-483]*** |
|  | **On the Discussions** |  |
| 1. | **Deeper Critical Interpretation**  Much of the discussion reiterates the results instead of providing critical analysis. Missing is an exploration of why specific findings occurred, such as cultural norms influencing women’s preference for pPR versus men’s reluctance to visit facilities. | Thank you for this comment. We believe our discussion provides an interpretation linking the observed differences to factors such as stigma/discrimination, gender power relationships, provider attitudes, relational dynamics, cost efficiency, strategies for partner notification with and without HIVST, and including the structural barriers. However, as suggested, we have included a paragraph highlighting the socio-cultural norms as stated below.  *“…Prior studies also revealed that stigma and discrimination are the key barriers for men and women in accessing HIV testing and care. However, owing to sociocultural norms, such barriers appear to occur differently across genders. For example, women prioritise dyadic harmony and personal safety in HIV disclosure to mitigate risks of violence and discrimination [126,127]. While the masculinity expectations and privacy concerns of the men discourage them from seeking facility-based testing in many low- and middle-income settings [125–127]…”*  ***[Please refer to track change lines #613-619]*** |
| 2. | **Equity and Key Populations**  While key populations (MSM, FSWs, PWID) are mentioned in the results, the discussion does not thoroughly examine differences in acceptability or feasibility based on gender, risk group, or relationship dynamics. It also lacks consideration of intersectionality aspects, such as stigma among MSM, safety risks faced by sex workers, and legal barriers encountered by PWID. | We addressed these comments earlier under your **Equity and Subgroup Analyses** feedback#3 under the Result section.  We have included a paragraph in the discussions section as stated below  *“…Differences in acceptability and feasibility vary by population, gender, and type of relationship. This shows the intersections of stigma, safety, and the legal environment among MSM and FSW, hindering disclosure and partner notification. For the women living with HIV, privacy and safety shaped their preferences for primary versus casual sexual partners. For PWID, legal and policy barriers inhibited their participation in index testing and partner notification. This highlights the importance of implementing rights-sensitive and context-specific HIVST and partner notification services, particularly in LMICs…”*  ***[Please refer to track change lines #643-650***]. |
| 3. | **Integration of Quantitative and Qualitative Evidence**  Findings are discussed separately, but there is no combined synthesis demonstrating how qualitative data clarifies quantitative results.  Missing: explicit integration using frameworks or joint interpretation (for example, uptake is higher in RCTs, but qualitative data shows the influence of trust, confidentiality, and gender dynamics). | Thank you for highlighting this important point. The same comment was raised earlier under the Result section, feedback #4. See a summary below.  The combined interpretation of both types of data in Table 2 provides a clear overview of how quantitative data clarifies qualitative outcomes. This aligns with the JBI convergent segregated approach for mixed-methods reviews.  ***[Please refer to track change lines #500-501]*** |
| 4. | **Policy and Implementation Context**  The text references cost and provider perspectives but lacks analysis of legal and policy environments, such as criminalization, consent laws, and WHO/UNAIDS guidelines. It also overlooks the explicit consideration of how policy contexts in LMICs influence feasibility and scalability. | We appreciate this valuable comment. Our Discussion highlights policy and legal considerations related to provider referral, confidentiality, and the need for uniform protocols. To make this more explicit, we have added a sentence linking these issues to broader WHO and UNAIDS policy guidelines as stated below:  *“The WHO and UNAIDS policy guidelines also highlight that differences in national legal frameworks, informed consent requirements, and the criminalization of key populations in many LMICs continue to undermine the HIV response, including feasibility, scalability, and integration of aPN and HIVST into existing partner notification services”*  ***[Please refer to track change lines #632-635].*** |
| 5. | **Comparison to Other Reviews/Guidelines**  The discussion does not compare its findings with existing systematic reviews, meta-analyses, or WHO guidelines. It also lacks a clear statement on how this review advances knowledge or differs from previous evidence syntheses. | Thank you for your comments. In our revised manuscript, we have added supporting evidence and included the WHO/UNAIDS guidelines, as shown below.  *“…Further, a previous review indicated that discussions on sexual risk and prevention were facilitated by HIVST, leading to partner testing among FSWs [111]…”.* ***[Please see track change lines #555-557]***  *“…The previous review also stressed the importance balanced and context-specific approach to improve HIV partner notification among key populations in LMICs by harnessing facilitators alongside addressing barriers [111]…”* ***[Please refer to track change lines #570-572].***  *“…A previous meta-analysis found that HIVST is a safe and effective intervention among the general population with a range of delivery models, identifying and linking additional people with HIV to care…”* ***[Please refer to track change lines #542-544].***  *“…Similarly, a meta-analysis also revealed that assisted HIVST or secondary distribution resulted in higher linkage to confirmatory testing and ART initiation [115]...”* ***[Please see to track change lines #520-522].***  *“…Overall, these review findings are consistent with the recommendations current WHO and UNAIDS guidelines, underlining the importance of integrating HIVST for index testing within partner notification services to enhance case diagnosis and linkage to treatment.…”* ***[Please refer to track change lines #650-652].***  Our discussion aligns with the recommendations of the WHO and UNAIDS. Additionally, it highlights the novelty as the first mixed-methods synthesis comparing HIVST and aPN from uptake to linkage and care, including strategies across LMICs. |
| 6. | **Strengths and Limitations**  A brief strengths/limitations note exists, but it underplays key issues:   - Lack of non-English studies. - Exclusion of unpublished or ongoing trials. - No sensitivity analyses or heterogeneity exploration. - Absence of GRADE/CERQual certainty assessments. | Thank you for this helpful suggestion. We have added the following text in the revised manuscript to make the study limitations more transparent.  *“…Unpublished or ongoing trials were not included, and while GRADE or CERQual assessments were not applied, methodological quality and bias were rigorously appraised using the MetaQAT framework. …”*  ***[Please refer to track change lines# 662-664]***  *The search strategy was limited to four databases, which may have missed some relevant studies from LMICs published in sources such as LILACS or African Index Medicus. No grey literature was reviewed, and we may have missed studies reported outside of the peer-reviewed literature. However, the reference lists of the selected articles were examined for additional eligible articles”*  ***[Please refer to track change lines# 666-670].*** |
| 7. | **Implications for Future Research and Practice**  The section highlights the need for additional research in Asia but remains vague about future directions. It lacks concrete recommendations, such as conducting trials with unmarried partners, developing interventions to address IPV risk, conducting implementation research in criminalized settings, and assessing cost-effectiveness in real-world programs. | Thank you for this insightful comment. The discussion highlights the key evidence gaps in the South and East Asia region, including the need for studies among unmarried populations and key populations, as well as cost-effectiveness analyses. We have intentionally not detailed future study methodologies, as our primary aim was to synthesise existing evidence on the feasibility and acceptability of HIVST for index testing and partner notification services. The discussion highlights the importance of future research to understand the unique barriers and needs of diverse populations, thereby ensuring inclusive and effective partner services [Please refer to track change lines ***527-529].***  However, as suggested, we have refined the sentence to specify the importance of contextual study methods as stated below (editions highlighted in yellow)  “…Future research should prioritize understanding the unique barriers and needs of these populations to ensure inclusive and effective partner services through contextually appropriate methods in LMICs..”  ***[Please refer to track change line #529].*** |
|  | **On Conclusions** |  |
| 1. | **Overly Descriptive, Not Interpretive**  The conclusion summaries the findings but lacks a strong final interpretation of what the evidence implies for policy, practice, and research. It also misses an interpretive “so what?” statement that clarifies the review's significance. | Thank you for your feedback. We would like to clarify that the conclusion clearly summarizes by identifying *why* HIVST and aPN are feasible (privacy, convenience, policy support) and what hinders them (stigma, gender norms, mistrust). |
| 2. | **No Explicit Policy or Program Implications**  The conclusion does not detail how the findings can inform HIV programs in LMICs, such as by integrating them into national HIV testing strategies, partner notification protocols, or assessing their cost-effectiveness for scaling up. It also omits practical advice for policymakers and implementers. | Thank you for the valuable feedback. We would like to clarify that our conclusion already references policy and implementation barriers **(see track change lines #683-684)** and cost-effectiveness **(see track change line #687).** |
| 3. | **Equity and Key Populations**  Although gaps are recognised for unmarried index cases and undiagnosed KPs, the conclusion does not emphasise the equity issues related to gender, stigma, criminalisation, and marginalisation. It also does not explicitly state that interventions should be designed with sensitivity to these factors. | Thank you for re-emphasizing this feedback. We have added a sentence in the conclusion as stated below.  *“…This review also reveals the intersection of stigma, safety and legal environment among MSM, FSW and PWID populations hindering disclosure and partner notification…”*  ***[Please refer to track change lines #695-697].*** |
| 4. | **No Link to Global Targets**  The conclusion does not connect the findings to the UNAIDS 95-95-95 targets or WHO guidelines, reducing its global health relevance. It also fails to link the presented evidence with ongoing global HIV elimination initiatives. | Thank you for this helpful comment. We have added a sentence to the conclusion as stated below;  *“…Finally, this review demonstrates that integrating HIVST within partner notification services presents an effective alternative testing option to reach the untested and undiagnosed individuals, thereby contributing to achieving the UNAIDS 95-95-95 in LMIC countries...”.*    ***[Please refer to track change lines #702-705]*** |
| 5. | **Future Research Agenda**  Although further research is acknowledged as necessary, the conclusion does not specify which particular study types are most needed—such as RCTs involving unmarried partners, IPV monitoring, implementation trials in Asia, or cost-effectiveness analyses of community-led programs. It also lacks clear, actionable priorities for future research. | As highlighted in your feedback #7 for the discussion, we have included a sentence to reflect the importance of contextualising future study methods, as stated below.  *“…Future research should prioritise contextually appropriate methods in LMICs to overcome the sociocultural and structural barriers, including relational complexities among varied populations…”*  ***[Please refer to track change lines #693-695]*** |
| 6. | **Strengths and Limitations Integration**  The conclusion does not address the review’s own limitations, such as regional bias towards Africa, the absence of a meta-analysis, and the exclusion of grey or non-English studies. It lacks a balanced summary that discusses how these limitations influence the interpretation of the findings. | We appreciate this comment. The review’s strengths and limitations are addressed in the Discussion ***[Please refer to tracked change lines #654-676].*** To avoid redundancy, we have not included them again here, but have written a summary sentence as stated below;  *“…This review's strength lies in its robust search strategy, use of the JBI convergent segregated method, and meta-aggregation. However, the study heterogeneity limited the meta-analysis, and restricting to English may have excluded relevant non-English studies...”*  ***[Please refer to track change lines # 700-702].*** |
